# Bioactivity and structure-activity relationship of cinnamic acid esters and their derivatives as potential antifungal agents for plant protection

**DOI:** 10.1371/journal.pone.0176189

**Published:** 2017-04-19

**Authors:** Kun Zhou, Dongdong Chen, Bin Li, Bingyu Zhang, Fang Miao, Le Zhou

**Affiliations:** 1 College of Chemistry & Pharmacy, Northwest A&F University, Yangling, Shaanxi, People’s Republic of China; 2 College of Life Science, Northwest A&F University, Yangling, Shaanxi, People’s Republic of China; Korea University, REPUBLIC OF KOREA

## Abstract

A series of cinnamic acid esters and their derivatives were synthesized and evaluated for antifungal activities in vitro against four plant pathogenic fungi by using the mycelium growth rate method. Structure−activity relationship was derived also. Almost all of the compounds showed some inhibition activity on each of the fungi at 0.5 mM. Eight compounds showed the higher average activity with average EC_50_ values of 17.4–28.6 μg/mL for the fungi than kresoxim-methyl, a commercial fungicide standard, and ten compounds were much more active than commercial fungicide standards carbendazim against *P*. *grisea* or kresoxim-methyl against both *P*. *grisea* and *Valsa mali*. Compounds **C1** and **C2** showed the higher activity with average EC_50_ values of 17.4 and 18.5 μg/mL and great potential for development of new plant antifungal agents. The structure−activity relationship analysis showed that both the substitution pattern of the phenyl ring and the alkyl group in the alcohol moiety significantly influences the activity. There exists complexly comprehensive effect between the substituents on the phenyl ring and the alkyl group in the alcohol moiety on the activity. Thus, cinnamic acid esters showed great potential the development of new antifungal agents for plant protection due to high activity, natural compounds or natural compound framework, simple structure, easy preparation, low-cost and environmentally friendly.

## Introduction

Plant mycosis is an important problem of agricultural production worldwide [1] and often results in severe yield losses and quality decrease of agricultural products. Furthermore, many of the fungi can harm animal and human health due to their mycotoxins [2]. Therefore, various fungicides have been extensively used to control fungal plant diseases in current agriculture. However, the prolonged usage of some antifungal agents can lead to drug resistance, environmental problems and residue toxicity [3]. Thus, it is necessary to develop environmentally friendly plant fungicides. In the past decades, researchers have already put attention to natural product-based or -derived plant protectants due to lower environmental and mammalian toxicity of natural products [4].

Cinnamic acid esters and their derivatives are widely distributed in plants including cereals, legumes, oilseeds, fruits, vegetables and tea or coffee beverages[5]. Due to the common occurrence in plants and the low toxicity for humans, animals and environment [6,7], cinnamic acid derivatives have attracted much attention of many pharmacologists. In the past decades, cinnamic acid derivatives including natural and non-natural compounds had been proved to possess diverse pharmacological actions such as antimicrobial [8–11], anti-*Mycobactrium tuberculosis* [12], anticancer [13], anti-inflammatory [14], anti-human immunodeficiency virus (HIV) [15], antiparasitic [16,17], inducing neural progenitor cell proliferation [18] and so on. Athough cinnamic acid itself and its derivative cinnaldehyde had been found to have inhibition activity against some plant pathogenic fungi [19–21], few reports have been found on systematic investigation on the activity of cinnamic acid esters against plant pathogenic fungi. The purpose of the present research is to explore the bioactivity of a series of cinnamic acid esters and their derivatives against phyto-pathogenic fungi and structure-activity relationship (SAR), and meanwhile discover new potent antifungal compounds.

This study involves three series of the target compounds: ethyl cinnamates with various substituents on the phenyl ring (**A**), cinnamic acid esters with various alkyl groups in the alcohol moiety (**B**) and *t*-butyl or *t*-amyl cinnamates with various substituents on the phenyl ring (**C**).

## Materials and methods

### Chemicals

Kresoxim-methyl (>99%) and carbendazim (>99%) were purchased from Aladdin Co. Ltd. (Shanghai, China). Dimethyl sulfoxide (DMSO) was purchased from J&K Chemical Ltd. (Beijing, China). Other reagents and solvents were obtained locally and of analytical grade or purified according to standard methods before use. The water used was redistilled and ion-free.

### Fungi

Plant pathogenic fungi, *Fusarium solani*, *Pyricularia grisea*, *Valsa mali* and *Botryosphaeria dothidea*, were provided by the Center of Pesticide Research, Northwest A&F University, China. The fungi were grown on potato dextrose agar (PDA) plates at 25°C and maintained at 4°C with periodic subculturing.

### Instruments

Melting points were determined on an XT-4 micro-melting point apparatus. Nuclear magnetic resonance spectra (NMR) were performed on an Avance III 500 MHz instrument (Bruker, Karlsruhe, Germany). Chemical shifts (*δ* values) and coupling constants (*J* values) are given in ppm and Hz, respectively. High resolution mass spectra (HR-MS) were carried out with a microTOF-QⅡ instrument (Bruker, Karlsruhe, Germany).

### Synthesis

**Fig 1:**
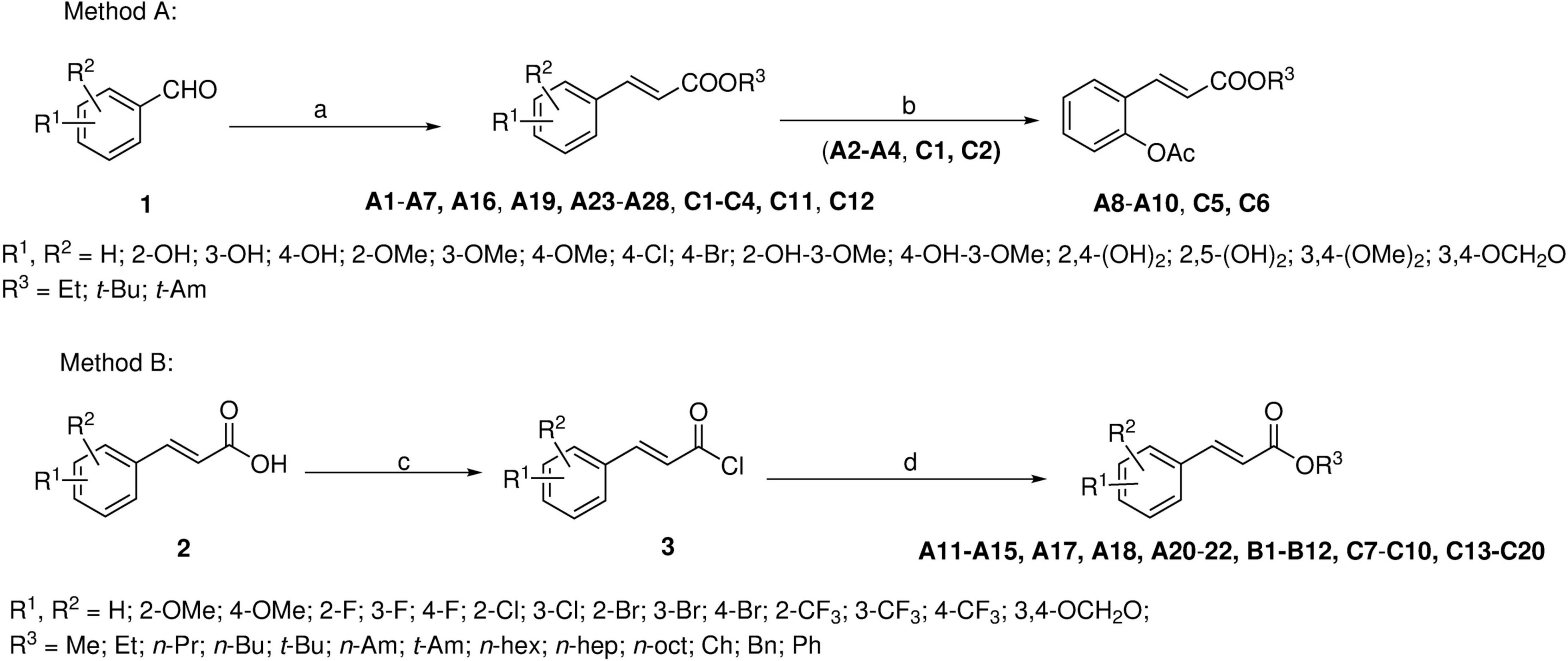
**Synthetic routes of compounds A, B and C. Reagents and conditions**. (a) Ph_3_P = CHCO_2_R^3^, EtOH or toluene, reflux, 1−4 h; (b) acetic anhydride, Et_3_N, r.t., 1 h; (c) SOCl_2_, reflux, 2 h; (d) R′OH, DCM, 0°C, 1 h.

Compounds **A1**−**A7**, **A16**, **A19**, **A23**−**A28**, **C1**−**C4**, **C11** and **C12** were prepared by our previously reported methods (method A in Fig 1) [16]. The general procedure is as follows. In brief, the solution of triphenylphosphanylidene acetate (Ph_3_P = CHCO_2_R, R = ethyl, *t*-butyl or *t*-amyl) (12 mmol) and aromatic aldehyde (10 mmol) in 50 mL toluene was refluxed for 1–4 h. After removal of the solvent, the resulting residue was subjected to a silica gel column chromatography (*ϕ* 40 mm×*L* 40 cm) using petroleum ether–ethyl acetate as eluent to yield the desired compounds.

#### Compounds A1−A7, A16, A19, A25−A28

The NMR data of the compounds were consistent with those previously reported by us [16].

#### Compounds A23, C1 and C3

Ethyl 2,4-dihydroxycinnamate (**A23**) [22], *t*-butyl 2-hydroxycinnamate (**C1**) [23] and *t*-butyl 4-hydroxycinnamate (**C3**) [24] were confirmed by comparison of NMR data with those in literature.

#### Ethyl 2,5-dihydroxycinnamate (A24)

Yield: 54% yield; a pale brown crystal; mp: 118–120°C; ^1^H NMR (500 MHz, CD_3_OD): *δ* 7.91 (1H, d, *J* = 16.0 Hz), 6.90 (1H, s), 6.69 (2H, s), 6.46 (1H, d, *J =* 16.0 Hz), 4.20 (2H, q, *J =* 7.2 Hz), 1.30 (3H, t, *J =* 7.2 Hz); ^13^C NMR (125 MHz, CD_3_OD): *δ* 169.5, 151.5, 151.3, 141.9, 122.9, 120.2, 118.1, 117.9, 114.6, 61.5, 14.6; Negative ESI-MS *m*/*z*: 206.9 [M-H]^-^.

#### t-Amyl 2-hydroxycinnamate (C2)

Yield: 60%; a yellow oil; ^1^H NMR (500 MHz, CDCl_3_): *δ* 7.99 (1H, d, *J* = 16.1 Hz), 7.45 (1H, dd, *J* = 7.6, 1.2 Hz), 7.30 (1H, s, OH), 7.22 (1H, 2×t, *J* = 7.9, 1.4 Hz), 6.90 (2H, q, *J* = 7.6 Hz), 6.59 (1H, d, *J* = 16.1 Hz), 1.90 (2H, q, *J* = 7.4 Hz), 1.52 (6H, s), 0.95 (3H, t, *J* = 7.4 Hz); ^13^C NMR (125 MHz, CDCl_3_): *δ* 167.9, 155.6, 139.7, 131.1, 129.0, 121.9, 120.4, 118.3, 116.3, 83.2, 33.6, 25.7, 8.2; HR-ESI-MS [M+Na]^+^ calcd for C_14_H_18_NaO_3_^+^, 257.1148, found 257.1157.

#### t-Amyl 4-hydroxycinnamate (C4)

Yield: 64%; a yellow oil; ^1^H NMR (500 MHz, CDCl_3_): *δ* 7.54 (1H, d, *J* = 15.9 Hz), 7.39 (2H, d, *J* = 8.6 Hz), 6.85 (2H, d, *J* = 8.5 Hz), 6.60 (1H, s, OH), 6.24 (1H, d, *J* = 15.9 Hz), 1.88 (2H, q, *J* = 7.4 Hz), 1.50 (6H, s), 0.94 (3H, t, *J* = 7.4 Hz); ^13^C NMR (125 MHz, CDCl_3_): *δ* 167.2, 158.0, 143.6, 129.8, 127.0, 115.8, 114.9, 83.1, 33.5, 25.7, 8.2; HR-ESI-MS [M+Na]^+^ calcd for C_14_H_18_NaO_3_^+^, 257.1148, found 257.1159.

#### t-Butyl 2-hydroxy-3-methoxycinnamate (C11)

Yield: 79%; a yellow powder; mp: 76–77°C; ^1^H NMR (500 MHz, CDCl_3_): *δ* 7.87 (1H, d, *J* = 16.1 Hz), 7.07 (1H, dd, *J* = 6.9, 2.3 Hz), 6.82–6.83 (2H, m), 6.53 (1H, d, *J* = 16.1 Hz), 6.18 (1H, s, OH), 3.89 (3H, s), 1.53 (9H, s); ^13^C NMR (125 MHz, CDCl_3_): *δ* 166.8, 146.8, 145.1, 138.4, 121.1, 121.0, 120.8, 119.5, 111.4, 80.2, 56.1, 28.2; HR-ESI-MS [M+Na]^+^ calcd for C_14_H_18_NaO_4_^+^, 273.1097, found 273.1132.

#### t-Amyl 2-hydroxy-3-methoxycinnamate (C12)

Yield: 63%; a white powder; mp: 76.2–77°C; ^1^H NMR (500 MHz, CDCl_3_): *δ* 7.87 (1H, d, *J* = 16.1 Hz), 7.08 (1H, dd, *J* = 7.1, 2.1 Hz), 6.82–6.85 (2H, m), 6.53 (1H, d, *J* = 16.1 Hz), 6.15 (1H, s, OH), 3.90 (3H, s), 1.88 (2H, q, *J* = 7.4 Hz), 1.50 (6H, s), 0.94 (3H, t, *J* = 7.4 Hz); ^13^C NMR (125 MHz, CDCl_3_): *δ* 166.7, 146.8, 145.1, 138.3, 121.16, 121.11, 120.8, 119.5, 111.4, 82.6, 56.1, 33.5, 25.7, 8.2; HR-ESI-MS [M+Na]^+^ calcd for C_15_H_20_NaO_4_^+^, 287.1254, found 287.1254.

**Compounds A11**−**A15, A17, A18, A20**−**A22, B1**−**B12, C7**−**C10** and **C13**−**C20** were prepared according to method B in Fig 1 by reaction of the corresponding acyl chloride and the corresponding alcohol. The general procedure was as follows. The mixture of cinnamic acid or cinnamic acids with substituents on the phenyl ring (0.10 mol) and 30 mL thionyl chloride was refluxed at 75°C for 2 h. The excess thionyl chloride was removed under reduced pressure. After the residue was dissolved in 10 mL DCM, the corresponding alcohol (10 mmol) was added at 0°C. The solution was stirred at 0°C for 1 h, and then washed with water (3 × 30 mL) followed by 5% Na_2_CO_3_ aqueous solution, and dried over anhydrous sodium sulfate. After filtration, the solvent was removed under reduced pressure. The residue was purified by silica gel column chromatography (*ϕ* 40 mm × *L* 40 cm) to afford the desired product.

#### Compounds A11-A15, A17, A18, A20-A22, B1-B5, B7-B12, C7, C9, C13, C15, C17 and C19

Compounds ethyl ethyl 2-fluorocinnamate (**A11**) [25], ethyl 3-fluorocinnamate (**A12**) [26], ethyl 4-fluorocinnamate (**A13**) [27], ethyl 2-chlorocinnamate (**A14**) [28], ethyl 3-chlorocinnamate (**A15**) [29], 2-bromocinnamate (**A17**) [25], ethyl 3-Bromocinnamate (**A18**) [30], ethyl 2-trifluoromethylcinnamate (**A20**) [25], ethyl 3-trifluoromethylcinnamate (**A21**) [26], ethyl 4-trifluoromethylcinnamate (**A22**) [27], methyl cinnamate (**B1**) [25], *n*-propyl cinnamate (**B2**) [31], *n*-butyl cinnamate (**B3**) [31], *t*-butyl cinnamate (**B4**) [32], *n*-amyl cinnamate (**B5**) [33], *n*-hexyl cinnamate (**B7**) [34], *n*-heptyl cinnamate (**B8**) [35], *n*-octyl cinnamate (**B9**) [36], cyclohexyl cinnamate (**B10**) [32], benzyl cinnamate (**B11**) [37], phenyl cinnamate (**B12**) [37], *t*-butyl 2-methoxycinnamate (**C7**) [38], *t*-butyl 4-methoxycinnamate (**C9**) [38], *t*-butyl 4-fluorocinnamate (**C13**) [39], *t*-butyl 4-bromocinnamate (**C15**) [39], *t*-butyl 4-trifluoromethylcinnamate (**C17**) [39] and *t*-butyl 3,4-methylenedioxy cinnamate (**C19**) [40] were confirmed by comparison of NMR data with those in literature.

#### t-Amyl cinnamate (B6)

Yield: 72%; a colorless oil; ^1^H NMR (500 MHz, CDCl_3_): *δ* 7.59 (1H, d, *J* = 16.0 Hz), 7.50–7.52 (2H, m), 7.36–7.37 (3H, m), 6.38 (1H, d, *J* = 16.0 Hz), 1.86 (2H, q, *J* = 7.5 Hz), 1.50 (6H, s), 0.93 (3H, t, *J* = 7.5 Hz); ^13^C NMR (125 MHz, CDCl_3_): *δ* 166.3, 143.5, 134.7, 130.0, 128.8, 128.0, 120.2, 83.0, 33.6, 25.7, 8.3.

#### t-Amyl 2-methoxycinnamate (C8)

Yield: 76%; a yellow oil; ^1^H NMR (500 MHz, DMSO-*d*_6_): *δ* 7.82 (1H, d, *J* = 16.1 Hz), 7.68 (1H, dd, *J* = 7.7, 1.3 Hz), 7.38–7.42 (1H, m), 7.09 (1H, d, *J* = 8.3 Hz), 6.99 (1H, t, *J* = 7.4 Hz), 6.51 (1H, d, *J* = 16.1 Hz), 3.86 (3H, s), 1.82 (2H, q, *J* = 7.5 Hz), 1.44 (6H, s), 0.89 (3H, t, *J* = 7.4 Hz); ^13^C NMR (125 MHz, DMSO-*d*_6_): *δ* 166.2, 158.2, 138.7, 132.2, 128.9, 122.8, 121.1, 120.3, 112.1, 82.5, 56.1, 33.2, 25.8, 8.5; HR-ESI-MS [M+Na]^+^ calcd for C_15_H_20_NaO_3_^+^, 271.1305; found 271.1312.

#### t-Amyl 4-methoxycinnamate (C10)

Yield: 87%; a yellow oil; ^1^H NMR (500 MHz, CDCl_3_): *δ* 7.54 (1H, d, *J* = 16.0 Hz), 7.45 (2H, dt, *J* = 8.8, 1.9 Hz), 6.88 (2H, d, *J* = 8.8 Hz), 6.25 (1H, d, *J* = 16.0 Hz), 3.82 (3H, s), 1.86 (2H, q, *J* = 7.5 Hz), 1.49 (6H, s), 0.93 (3H, t, *J* = 7.5 Hz); ^13^C NMR (125 MHz, CDCl_3_): *δ* 166.6, 161.4, 143.2, 129.6, 127.4, 117.7, 114.3, 82.7, 55.3, 33.6, 25.7, 8.3; HR-ESI-MS [M+Na]^+^ calcd for C_15_H_20_NaO_3_^+^, 271.1305, found 271.1310. [M+K]^+^ calcd for C_15_H_20_KO_3_^+^, 287.1044; found 287.1048.

#### t-Amyl 4-fluorocinnamate (C14)

Yield: 93%; yellow oil; ^1^H NMR (500 MHz, DMSO-*d*_6_): *δ* 7.77–7.78 (2H, m), 7.58 (1H, d, *J* = 16.0 Hz), 7.22–7.25 (2H, m), 6.51 (1H, d, *J* = 16.0 Hz), 1.83 (2H, q, *J* = 7.4 Hz), 1.45 (6H, s), 0.90 (3H, t, *J* = 7.4); ^13^C NMR (125 MHz, CDCl_3_): *δ* 166.1, 163.7 (d, *J* = 250.6 Hz), 142.2, 130.9 (d, *J* = 3.5 Hz), 129.8 (d, *J* = 8.3), 120.0 (d, *J* = 2.2. Hz), 116.0 (d, *J* = 21.9 Hz), 83.1, 33.5, 25.7, 8.3; HR-ESI-MS [M+Na]^+^ calcd for C_14_H_17_FNaO_2_^+^, 259.1105; found 259.1113.

#### t-Amyl 4-bromocinnamate (C16)

Yield: 95%; a yellow oil; ^1^H NMR (500 MHz, DMSO-*d*_6_): *δ* 7.59–7.66 (4H, m), 7.55 (1H, d, *J* = 16.0 Hz), 6.58 (1H, d, *J* = 16.0 Hz), 1.83 (2H, q, *J* = 7.5 Hz), 1.45 (6H, s), 0.90 (3H, t, *J* = 7.5 Hz); ^13^C NMR (125 MHz, DMSO-*d*_6_): *δ* 165.7, 142.6, 133.9, 132.2, 130.5, 124.0, 121.1, 82.8, 33.2, 25.8, 8.5; HR-ESI-MS [M+Na]^+^ calcd for C_14_H_17_^79^BrNaO_2_^+^, 319.0304, found 319.0302, calcd for C_14_H_17_^81^BrNaO_2_^+^, 321.0284; found 321.0280.

#### t-Amyl 4-trifluoromethylcinnamate (C18)

Yield: 92%; a yellow oil; ^1^H NMR (500 MHz, DMSO-*d*_6_): *δ* 7.93 (2H, d, *J* = 8.2 Hz), 7.76 (2H, d, *J* = 8.3 Hz), 7.65 (1H, d, *J* = 16.0 Hz), 6.70 (1H, d, *J* = 16.0 Hz), 1.86 (2H, q, *J* = 7.5 Hz), 1.46 (6H, s), 0.91 (3H, t, *J* = 7.5 Hz); ^13^C NMR (125 MHz, DMSO-d_6_): δ 165.5, 142.1, 138.6, 130.3 (q, *J* = 31.9 Hz), 129.3, 126.1 (q, *J* = 3.6 Hz), 124.5 (q, *J* = 271.8 Hz), 123.2, 83.1, 33.2, 25.8, 8.5; HR-ESI-MS [M+Na]^+^ calcd for C_15_H_17_F_3_NaO_2_^+^, 309.1073; found 309.1068.

#### t-Amyl 3,4-methylenedioxy cinnamate (C20)

Yield: 85%; a white powder; mp: 47–48°C; ^1^H NMR (500 MHz, CDCl_3_): *δ* 7.49 (1H, d, *J* = 15.9 Hz), 7.01 (1H, d, *J* = 1.6 Hz), 6.97 (1H, dd, *J* = 8.0, 1.6 Hz), 6.79 (1H, d, *J* = 8.0 Hz), 6.20 (1H, d, *J* = 15.9 Hz), 5.99 (2H, s), 1.85 (2H, q, *J* = 7.5 Hz), 1.49 (6H, s), 0.92 (3H, t, *J* = 7.5 Hz); ^13^C NMR (125 MHz, CDCl_3_): *δ* 166.5, 149.3, 148.3, 143.2, 129.1, 124.1, 118.2, 108.5, 106.5, 101.5, 82.8, 33.5, 25.7, 8.3; HR-ESI-MS [M+Na]^+^ calcd for C_15_H_18_NaO_4_^+^, 285.1097, found 285.1099; [M+K]^+^ calcd for C_15_H_18_KO_4_^+^, 301.0837; found 301.0841.

#### Synthesis of compounds A8-A10, C5 and C6

According to our previously reported method [16], compounds **A8**−**A10**, **C5** and **C6** were prepared by acetylation reaction of the corresponding phenols. Briefly, the solution of compounds **A2**, **A3**, **A4**, **C1** or **C2** (2.1 mmol) and acetic anhydride (0.6 g, 6.2 mmol) in 20 mL triethylamine was stirred for 1 h at room temperature to provide the desired compounds.

#### Compounds A8-A10 and C5

The NMR and MS data of compounds **A8-A10** [26] and **C5** [41] were consistent with those in the literature.

#### t-Amyl 2-acetoxyl cinnamate (C6)

Yield: 98%; a yellow oil; ^1^H NMR (500 MHz, CDCl_3_): *δ* 7.67 (2H, m), 7.39 (1H, 2×t, *J* = 8.0, 1.6 Hz), 7.24 (1H, d, *J* = 7.7 Hz), 7.11 (1H, dd, *J* = 8.1, 0.9 Hz), 6.40 (1H, d, *J* = 16.0 Hz), 2.36 (3H, s), 1.86 (2H, q, *J* = 7.5 Hz), 1.49 (6H, s), 0.94 (3H, t, *J* = 7.4 Hz); ^13^C NMR (125 MHz, CDCl_3_): *δ* 169.1, 165.9, 149.2, 136.6, 130.8, 127.4, 127.3, 126.3, 123.0, 122.1, 83.1, 33.6, 25.6, 20.9, 8.2; HR-ESI-MS [M+Na]^+^ calcd for C_16_H_20_NaO_4_^+^, 299.1254; found 299.1260.

### Antifungal assay

The mycelium linear growth rate method was used to screen the antifungal activities in vitro of compounds against four strains of plant pathogenic fungi [42]. Commercial fungicide standards kresoxim-methyl or carbendazim were used as positive controls.

Briefly, the solution of the test compound (50 μmol) in 0.5 mL of DMSO was added to 9.5 mL of sterile water. The resulting solution was added to 90 mL of melted PDA agar at 50°C and quickly and completely mixed to provide the mixture containing 0.5 mM of the compounds and 0.5% (v/v) DMSO. The mixture was poured into a sterilized Petri dish (ca. 15 mL each plate) in a laminar flow chamber. The culture medium containing only 0.5% DMSO was used as a blank without observable effect on the growth of the fungi. When the medium in the plate was partially solidified, a fungus disk (*d* = 5 mm) cut beforehand from subcultured Petri dishes was placed at the center of the medium. The dishes were kept in an incubator at 25°C for 72 h. Each experiment was carried out in triplicate. The diameters (in mm) of a fungal colony were measured in three different directions, and the growth inhibition rates were calculated according to the method reported previously [42].

The compounds with higher initial activities were further assayed for median effective concentrations (EC_50_) according to the method described above. A set of stock solutions of test compounds in 5% DMSO aqueous solution was prepared by double-fold dilution method. Each stock solution (10 mL) was mixed with autoclaved liquid PDA medium (190 mL) to provide a series of mediums with various concentrations of the test compound. The culture medium containing only 0.5% DMSO was used as a blank control. Each test was performed in triplicate. The probit value of the average inhibition rate for each test concentration and the corresponding lg[concentration (*μ*g/mL)] were used to establish antifungal toxicity regression equation by the linear least-square fitting method. The EC_50_ value of each compound and its confidence intervals at 95% probability were calculated from the corresponding toxicity regression equation by using PRISM software ver. 5.0 (GraphPad Software Inc., San Diego, CA,USA) [42].

Duncan multiple comparison test was used to evaluate significant difference between the average inhibition rates or EC_50_ values of various compounds by using PRISM software ver. 5.0 [42].

## Results

### Design of compounds

In order to know the effect of substitution patterns of the phenyl ring and the type of alkyl groups in the alcohol moiety on the activity, we firstly designed both **A** and **B** series of target compounds. **A** series consisting of 28 compounds is a class of ethyl cinnamate derivatives containing various substituents on the phenyl ring. **B** series including 12 compounds is a class of cinnamic acid esters formed by cinnamic acid and various alcohols or phenol. Next, under the guidance of SAR of compounds **A** and **B**, we further designed **C** series including 20 compounds by combination of preponderant active groups in order to further explore the comprehensive effect of the substituents on the phenyl ring and the alkyl groups in the alcohol moiety on the activity and find more potential compounds. Compounds **C** are a class of *t*-butyl or *t*-amyl cinnamates with various substituents on the phenyl ring (Fig 1).

### Synthesis of compounds

Compounds **A1**–**A7**, **A16**, **A19**, **A23**–**A28**, **C1**-**C4**, **C11** and **C12** were prepared by Wittig reaction of triphenylphosphanylidene acetate [Ph_3_P = CHCO_2_R′] and aromatic aldehyde in ethanol or toluene. Compounds **A2**–**A4**, **C1** and **C2** were acetylated with acetic anhydride to afford compounds **A8**–**A10**, **C5** and **C6**, respectively. Substituted cinnamoyl chlorides reacted with the corresponding alcohols to obtain compounds **A11**–**A15, A17, A18, A20**–**22, B1**–**12, C7**–**C10** and **C13**–**C20.** The synthetic route of compounds **A**, **B** and **C** is outlined in Fig 1. The substitution patterns of the compounds are listed in Tables 1, 2 and 3.

**Table 1:** Substitution patterns of compounds A1-A28 and initial antifungal activity at 0.5 mM (72 h).

Compound		Average inhibition rate (%) (around 100 μg/mL, 72 h)^*a*^				
No.	R^1^, R^2^	*F*. *solani*	*P*. *grisea*	*V*. *mali*	*B*. *dothidea*	Mean
**A1**	H	8.9 ± 2.6t	14.4 ± 0.7n	16.8 ± 1.8q	40.4 ± 1.2lm	20.1
**A2**	2-OH	84.7 ± 0.1d	80.1 ± 0.9a	82.1 ± 0.1e	91.4 ± 1.3b	84.6
**A3**	3-OH	32.9 ± 0.5no	47.9 ± 4.4j	48.8 ± 0.0jk	42.8 ± 0.9l	43.1
**A4**	4-OH	88.2 ± 1.5c	67.2 ± 1.1cde	54.4 ± 0.7i	65.8 ± 2.2g	68.9
**A5**	2-OMe	94.4 ± 2.5ab	53.9 ± 1.3h	97.6 ± 1.2a	77.0 ± 3.5e	80.7
**A6**	3-OMe	38.1 ± 1.0m	49.1 ± 0.9ij	40.6 ± 3.5lm	46.8 ± 1.2k	43.7
**A7**	4-OMe	60.4 ± 0.8j	53.6 ± 0.5h	53.3 ± 3.1i	73.6 ± 1.2f	60.2
**A8**	2-OAc	78.3 ± 2.0e	72.6 ± 0.0b	66.0 ± 2.2g	88.5 ± 1.2c	76.4
**A9**	3-OAc	28.5 ± 2.7p	70.5 ± 1.2bc	25.9 ± 4.0o	48.3 ± 1.0k	43.3
**A10**	4-OAc	35.3 ± 2.5n	55.1 ± 1.8h	38.1 ± 1.3m	70.6 ± 1.1f	49.8
**A11**	2-F	66.1 ± 3.0i	38.0 ± 1.3k	25.0 ± 1.3o	28.0 ± 1.5o	39.3
**A12**	3-F	68.2 ± 1.4hi	51.7 ± 1.7hi	42.2 ± 2.6l	56.9 ± 1.6i	54.8
**A13**	4-F	83.1 ± 1.5d	33.3 ± 1.2l	74.2 ± 2.0f	65.0 ± 1.9gh	63.9
**A14**	2-Cl	60.4 ± 0.8j	34.4 ± 3.0l	23.3 ± 1.7op	62.5 ± 0.3h	45.2
**A15**	3-Cl	18.0 ± 0.8r	17.4 ± 0.7n	50.0 ± 1.3jk	38.1 ± 2.0mn	30.8
**A16**	4-Cl	45.8 ± 2.2l	48.8 ± 4.1ij	85.0 ± 1.3d	58.3 ± 2.5i	59.5
**A17**	2-Br	32.0 ± 0.8o	62.3 ± 1.0fg	60.7 ± 1.4h	73.2 ± 1.0f	57.1
**A18**	3-Br	12.8 ± 3.5s	29.4 ± 0.5m	50.6 ± 0.6j	36.3 ± 2.6n	32.3
**A19**	4-Br	71.2 ± 1.6g	68.9 ± 1.1cd	84.8 ± 1.3de	53.3 ± 4.0j	69.6
**A20**	2-CF_3_	77.1 ± 1.2ef	32.2 ± 1.1lm	30.2 ± 1.3n	52.3 ± 0.9j	48.0
**A21**	3-CF_3_	69.7 ± 2.3gh	64.4 ± 1.1ef	47.4 ± 1.3k	46.5 ± 1.9k	57.0
**A22**	4-CF_3_	75.3 ± 2.4f	79.7 ± 0.6a	85.7 ± 0.7d	56.3 ± 0.8i	74.3
**A23**	2,4-(OH)_2_	52.5 ± 0.6k	54.2 ± 0.8h	23.8 ± 0.3op	40.9 ± 1.4lm	42.9
**A24**	2,5-(OH)_2_	21.2 ± 0.8q	35.3 ± 1.0kl	21.7 ± 0.4p	21.1 ± 1.0p	24.8
**A25**	2-OH-3-OMe	91.9 ± 0.5b	68.5 ± 2.9cd	88.5 ± 0.6c	83.3 ± 2.1d	83.1
**A26**	4-OH-3-OMe	18.5 ± 0.8qr	78.2 ± 1.6a	24.6 ± 0.4o	49.1 ± 2.3k	42.6
**A27**	3,4-(OMe)_2_	44.7 ± 1.1l	60.1 ± 6.3g	42.6 ± 2.1l	49.1 ± 1.2k	49.1
**A28**	3,4-OCH_2_O	40.1 ± 0.8m	73.2 ± 0.6b	74.8 ± 1.0f	62.4 ± 1.2h	62.6
Kresoxim-methyl		71.3 ± 0.1g	66.8 ± 1.1de	54.3 ± 0.7i	88.2 ± 1.5c	70.2
Carbendazim		96.1 ± 0.1a	5.3 ± 0.8o	93.9 ± 0.7b	94.4 ± 0.1a	72.4

^*a*^ The difference between the data with the different lowercase letters within a column is significant (*P <* 0.05).

**Table 2:** Substitution patterns of compounds B1−B12 and initial antifungal activity at 0.5 mM.

Compound		Average inhibition rate (%) (around 100 μg/mL, 72 h)^*a*^				
No.	R^3^	*F*. *solani*	*P*. *grisea*	*V*. *mali*	*B*. *dothidea*	Mean
**B1**	Methyl	32.6 ± 0.7g	20.6 ± 2.4i	24.6 ± 2.5h	3.6 ± 0.8l	20.4
**A1**	Ethyl	8.9 ± 2.6k	14.4 ± 0.7j	16.8 ± 1.8j	40.4 ± 1.2f	20.1
**B2**	*n*-Propyl	61.2 ± 1.1d	46.0 ± 1.4f	20.9 ± 0.9i	29.3 ± 0.5h	39.4
**B3**	*n*-Butyl	30.4 ± 0.9gh	55.1 ± 1.4d	60.0 ± 0.5c	41.5 ± 1.3f	46.8
**B4**	*t*-Butyl	82.6 ± 1.3b	79.3 ± 0.7a	30.2 ± 1.3f	100.0 ± 0.0a	73.0
**B5**	*n*-Amyl	28.6 ± 1.7h	54.0 ± 0.4d	47.7 ± 1.4e	11.3 ± 0.8k	35.4
**B6**	*t*-Amyl	73.2 ± 1.2c	80.5 ± 2.2a	78.6 ± 1.4b	91.9 ± 0.1c	81.1
**B7**	*n*-hexyl	35.8 ± 1.7f	50.8 ± 0.8e	46.0 ± 1.3e	10.5 ± 1.6k	35.8
**B8**	*n*-Heptyl	24.1 ± 1.5i	37.9 ± 0.9g	30.5 ± 2.3f	26.6 ± 0.9i	29.8
**B9**	*n*-octyl	19.5 ± 0.9j	33.3 ± 1.1h	27.0 ± 0.7g	9.0 ± 0.8k	22.2
**B10**	Cyclohexyl	39.7 ± 1.0e	60.3 ± 1.2c	56.4 ± 1.1d	50.9 ± 1.0e	51.8
**B11**	benzyl	38.9 ± 1.0e	50.8 ± 2.5e	55.3 ± 1.3d	33.8± 2.2g	44.7
**B12**	Phenyl	18.4 ± 2.1j	35.7 ± 2.7gh	15.5 ± 0.8j	22.5 ± 3.4j	23.0
Kresoxim-methyl		71.3 ± 0.1c	66.8 ± 1.1b	54.3 ± 0.7d	88.2 ± 1.5 d	70.2
Carbendazim		96.1 ± 0.1a	5.3 ± 0.8k	93.9 ± 0.7d	94.4 ± 0.1b	72.4

^*a*^ The difference between the data with the different lowercase letters within a column is significant (*P <* 0.05).

**Table 3:** Substitution patterns of compounds C1−C20 and initial antifungal activity at 0.5 mM.

Compound	Substituent		Average inhibition rate (%) (around 100 μg/mL, 72 h)^*a*^				
	R^1^, R^2^	R^3^	*F*. *solani*	*P*. *grisea*	*V*. *mali*	*B*. *dothidea*	Mean
**C1**	2-OH	*t*-Butyl	84.7 ± 0.1d	82.3 ± 1.6b	89.8 ± 0.1e	96.5 ± 0.1a	88.3
**C2**	2-OH	*t*-Amyl	91.9 ± 0.7b	87.1 ± 0.0ab	91.1 ± 1.2d	95.3 ± 0.1ab	91.4
**C3**	4-OH	*t*-Butyl	80.9 ± 1.2fg	91.4 ± 1.9a	92.3 ± 0.1c	93.0 ± 0.1c	89.4
**C4**	4-OH	*t*-Amyl	82.1 ± 0.1ef	82.3 ± 1.6b	82.1 ± 0.1g	93.4 ± 0.6c	85.0
**C5**	2-OAc	*t*-Butyl	83.4 ± 1.2de	82.3 ± 1.6b	86.0 ± 1.2f	93.0 ± 0.1c	86.2
**C6**	2-OAc	*t*-Amyl	89.4 ± 0.7c	89.2 ± 0.9a	88.9 ± 0.7e	94.1 ± 0.1bc	90.4
**C7**	2-OMe	*t*-Butyl	71.3 ± 3.7h	60.4 ± 0.7fg	64.1 ± 1.5k	77.5 ± 0.8f	68.3
**C8**	2-OMe	*t*-Amyl	55.3 ± 1.1l	59.1 ± 1.2g	58.6 ± 0.5l	63.8 ± 1.1k	59.2
**C9**	4-OMe	*t*-Butyl	58.9 ± 0.4 k	66.0 ± 1.4ef	54.9 ± 1.0o	75.2 ± 0.2g	63.8
**C10**	4-OMe	*t*-Amyl	64.6 ± 1.1i	69.7 ± 1.7de	61.3 ± 0.3l	78.1 ± 1.8f	68.4
**C11**	2-OH-3-OMe	*t*-Butyl	66.5 ± 1.1i	67.0 ± 0.6e	52.0 ± 0.7p	85.0 ± 2.0e	67.6
**C12**	2-OH-3-OMe	*t*-Amyl	79.7 ± 1.9g	65.8 ± 1.0ef	65.1 ± 0.6k	71.3 ± 0.4h	70.5
**C13**	4-F	*t*-Butyl	61.4 ± 1.0j	50.5 ± 0.9h	48.8 ± 0.0q	57.5 ± 0.1l	54.6
**C14**	4-F	*t*-Amyl	57.5 ± 0.4k	57.2 ± 0.7g	66.5 ± 0.4j	73.6 ± 0.5g	63.7
**C15**	4-Br	*t*-Butyl	44.2 ± 0.6n	57.7 ± 0.9g	50.0 ± 0.0q	66.4 ± 0.5j	54.6
**C16**	4-Br	*t*-Amyl	39.4 ± 1.3o	57.2 ± 0.7h	54.4 ± 0.8l	64.7 ± 0.8m	53.9
**C17**	4-CF_3_	*t*-Butyl	20.2 ± 0.8q	59.9 ± 0.2g	96.4 ± 1.2a	43.6 ± 2.1n	55.0
**C18**	4-CF_3_	*t*-Amyl	31.9 ± 0.9p	46.1 ± 1.0h	40.3 ± 0.7r	43.0 ± 1.5n	40.3
**C19**	3,4-OCH_2_O	*t*-Butyl	43.4 ± 1.2n	74.1 ± 0.2cd	73.6 ± 1.0j	66.5 ± 1.0j	64.4
**C20**	3,4-OCH_2_O	*t*-Amyl	51.3 ± 1.1lm	76.2 ± 0.7c	75.8 ± 0.8h	69.0 ± 1.1i	68.1
Kresoxim-methyl			71.3 ± 0.1h	66.8 ± 1.1e	54.3 ± 0.7o	88.2 ± 1.5d	70.2
Carbendazim			96.1±0.1a	5.3±0.8i	93.9±0.7b	94.4±0.1bc	72.4

^*a*^ The difference between the data with the different lowercase letters within a column is significant (*P <* 0.05).

The synthesized compounds include 11 new compounds and 49 known compounds. The known compounds were confirmed by comparison of NMR data and those reported in literature. The new compounds (**C2**, **C4**, **C6**, **C8**, **C10**–**C12**, **C14**, **C16**, **C18**, **C20**) were identified by ^1^H NMR, ^13^C NMR and HRMS analysis. In positive or negative ESI-MS spectra, each of the compounds showed its corresponding pseudo-molecular ion peak [M+Na]^+^. In ^1^H and ^13^C NMR spectra, all the compounds revealed two doublet signals in the ranges of 7.5 to 8.1 and *δ*_H_ 6.2 to 6.7 ppm due to the protons of the ethylene moiety and one signal in the range of *δ*_C_ 165 to 167 ppm due to C (= O). The coupling constant of ca. 16 Hz between the protons of the ethylene moiety showed *E* configuration of the compounds. The NMR data of the known compounds were agreement with the corresponding literature data.

### Antifungal activity

According to the mycelium linear growth rate method [39], the synthesized compounds were screened for antifungal activity in vitro at 0.5 mM (ca.100 μg/mL) against four plant pathogenic fungi. Kresoxim-methyl and carbendazim, two commercial fungicide standards, were used as positive controls.

The results listed in Tables 1, 2 and 3 showed that all the compounds displayed some inhibition activity against each of the fungi at 0.5 mM (around 100 μg/mL). Among A and B series, compounds **A2**, **A5**, **A8**, **A22**, **A25**, **B4** and **B6** showed the higher activity with average inhibition rates of 73‒84% for four tested fungi, superior to kresoxim-methyl and carbendazim (70.2%, 72.4%). For **A** series, **A2** showed the highest average activity (84.6%), followed by **A25** and **A5** (83.1%, 80.7%). Among **B** series, **B6** gave the highest average activity (81.1%) followed by B4 (73.0%). For **C** series, all the compounds showed the average inhibition rates of >50% except for **18**. Especially, **C1**-**C6** exhibited the very high average inhibition rates of 85–91% for all the fungi, obviously superior to all compounds **A**, **B**, kresoxim-methyl or carbendazim.

In order to explore the antifungal potential in more detail and SAR, some of the compounds (**A2**, **A4**, **A8**, **A25**, **C1**−**C6**) with the higher initial activity were further determined for median effective concentrations (EC_50_) against each of the fungi. The results are listed in Table 4. Seventy percent of all the test items (28/40) revealed good to excellent activity with EC_50_ values of 9.9−30 μg/mL. Compounds **A4**, **A25** and **C2** showed the highest activity against *F*. *solani* with EC_50_ values of 17.8−19.8 μg/mL (*P*<0.05). For *P grisea*, **C1** and **C2** showed the highest activity (EC_50_ = 15.4, 17.0 μg/mL) (*P*<0.05), followed by **A2** (EC_50_ = 18.3 μg/mL). Compounds **A25**, **C1** and **C3** displayed the strongest activity against *V mali* (EC_50_ = 18.4, 18.6, 21.1 μg/mL) (*P*<0.05). For *B*. *dothidea*, **C1**, **C1** and **C5** showed the highest activity with EC_50_ values of 11.4, 9.9 and 13.7 μg/mL (*P*<0.05), followed by **A2** with EC_50_ values of 17.5 μg/mL. As far as the average EC_50_ value of each compound for all the fungi was concerned, **C1** had the highest activity (average EC_50_ = 17.4 μg/mL), followed by **C2** (average EC_50_ = 18.5 μg/mL). It was worth noting that all the test compounds were much more active on *P*. *grisea* and *V*. *mali* than kresoxim-methyl (EC_50_ = 58.7, 98.5 μg/mL) or on *P*. *grisea* than carbendazim (EC_50_ > 100 μg/mL).

**Table 4:** EC_50_ values of the compounds with higher initial activity against four strains of fungi.

Compd.	EC_50_ (confidence intervals at 95% probability) (μg/mL, 72 h)^*a*^				
	*F*. *solani*	*P*. *grisea*	*V*. *mali*	*B*. *dothidea*	Mean
**A2**	26.1±0.5d (21.4–31.6)	18.3±0.5b (13.5–24.0)	42.6±0.8e (31.6–57.5)	17.5±0.8e (13.2–22.9)	26.1
**A4**	19.0±1.0c (13.8–25.7)	48.9±2.5f (36.3–63.1)	60.8±4.3f (42.7–97.7)	43.2±1.1i (34.7–52.5)	43.0
**A8**	32.8±2.0f (28.8–37.2)	46.0±1.6e (38.9–55.0)	59.1±2.8f (41.7–87.1)	21.0±1.7f (15.1–28.2)	39.7
**A25**	17.8±0.6c (14.8–21.4)	44.5±1.2e (36.3–55.0)	18.4±0.6b (13.5–24.5)	23.2±1.2g (20.4–26.9)	26.0
**C1**	24.1±0.8d (20.9–28.2)	15.4±0.1a (12.6–18.6)	18.6±0.8b (14.8–23.4)	11.4±0.0c (9.1–13.8)	**17.4**
**C2**	19.8±0.9c (17.0–22.9)	17.0±0.1ab (12.3–22.9)	27.4±1.4c (22.9–33.1)	9.9±0.1b (5.9–14.5)	**18.5**
**C3**	40.6±1.0g (34.7–47.9)	23.4±0.6cd (20.0–26.9)	21.1±0.2b (15.8–27.5)	23.2±0.7g (19.5–26.9)	27.1
**C4**	29.3±1.1e (24.5–35.5)	23.0±0.7c (21.4–24.5)	31.0±0.5c (22.9–41.7)	29.2±1.0h (25.7–33.1)	28.1
**C5**	42.2±1.4g (33.9–51.3)	25.7±0.4d (19.1–33.9)	29.2±0.4c (22.4–37.2)	13.7±0.1d (9.5–18.2)	27.7
**C6**	34.1±0.5f (25.1–45.7)	24.8±0.3cd (20.0–30.9)	34.9±0.5d (25.1–49.0)	20.4±0.7f (17.4–24.0)	28.6
**KM**^***b***^	14.2±4.4b (9.8–20.4)	58.7±2.6g (36.3–128.8)	98.5±4.5g (77.6–134.9)	4.3±0.1a (3.0–5.8)	43.9
**CBD**^***c***^	3.3±0.1a (2.5–4.4)	>100	2.8±0.1a (1.8–4.1)	3.6±0.1a (3.3–4.0)	

^*a*^The difference between the data with the different lowercase letters within a column is significant (*P <* 0.05)

^*b*^ KM: Kresoxim-methyl

^*c*^ CBD: carbendazim.

## Discussion—Structure-activity relationship

Comparison of the activities and structures of the compounds in Tables 1 and 2 showed that both substitution patterns of the benzene ring and the type of alkyl groups in the alcohol moiety significantly influence the activity of cinnamic esters. For ethyl cinnamate derivatives (**A** series), in most cases, the introduction of substituents to the benzene ring leads to increase of the activity. For electron-donating groups like OH, OMe, and OAc, the order of the activity of various position isomers is *o*-substituted isomer > *p*-substituted isomer > *m*-substituted isomer, whereas for electron-withdrawing groups like halogen atoms and trifluoromethyl, *p*-substituted isomers generally have the highest activity. 2,4-Dihydroxy or 2,5-dihydroxy compounds (**A23**, **A24**) are less active than the corresponding single hydroxyl compounds (**A23** vs **A2** or **A4**; **A24** vs **A2** or **A3**). For 2-OH-substituted compound (**A2**), the presence of additional 3-OMe has little effect on the activity (**A2** vs **A25**), whereas for 4-OH- or 4-OMe-substituted compounds, introduction of 3-OMe leads to significant decrease of the activity (**A4** vs **A26**; **A7** vs **A27**). Additionally, compared with 3,4-dimethoxy, 3,4-methylenedioxy can improve the activity (**A27** vs **A28**).

For **B** series, the activity of the linear alkyl esters (**A1**, **B1**−**B3**, **B5**, **B7**−**B9**) showed an obvious trend of increase at first and then decrease with elongation of the linear alkyl chain in the alcohol moiety (Table 2). The *n*-butyl ester (**B3**) showed the highest activity with an average inhibition rates of 46.8% for all the fungi, followed by the *n*-propyl ester (**B2**, 39.4%), *n*-amyl ester (**B5**, 35.4%) and *n*-hexyl ester (**B7**, 35.8%). Compared with linear alkyl groups, the corresponding tertiary alkyl groups can significantly increase the activity (**B3** vs **B4**; **B5** vs **B6**), showing that the steric hindrance near the ester group is helpful to improvement of the activity. The same trend was also observed by comparison of the activity of **B7** and **B10**. Replacement of the cyclohexyl with phenyl leads to decrease of the activity (**B12** vs **B10**), whereas replacement of the linear alkyl with benzyl (**B11**) causes slight increase of the activity.

By comparison of the activity of compounds **C** in Table 3 and that of the corresponding compounds **A** in Table 1 or **B** in Table 2, it was found that there exists a complex comprehensive effect between the substituent on the phenyl ring and the alkyl group in the alcohol moiety on the activity. The combinations of “2-OH, 4-OH or 2-OAc + *t*-butyl or *t*-amyl” (**C1**-**C6**) are able to create significantly synergistic enhancement effect on the activity. On the contrary, the combinations of “2-OMe, 4-OMe, 2-OH-3-OMe, 4-F, 4-Br, 4-CF_3_ or 3,4-OCH_2_O + *t*-butyl or *t*-amyl” (**C7**-**C20**) generally decrease the activity, compared with *t*-butyl or *t*-amyl cinnamates (**B4**, **B6**), or the corresponding substituted cinnamic acid ethyl esters (**A5**, **A7**, **A13**, **A19**, **A22**, **A25**, **A28**).

From the EC_50_ values in Table 4, it was further found that the comprehensive effect between the substituent on the phenyl ring and the alkyl group in the alcohol moiety on the activity varies with the species of the test fungi. The combinations of both “2-OH + *t*-butyl” (**C1**) and “2-OH + *t*-amyl” (**C2**) can significantly improve the activity against each of the fungi, relative to “2-OH + ethyl” (**A2**). By contrast, the combinations of “4-OH or 2-OAc + *t*-butyl or *t*-amyl” (**C1**−**C6**) can increase the activity on *P grisea*, *V mali* and *B*. *dothidea*, but decrease the activity on *F*. *solani* (**C3**, **C4** vs **A4**; **C5**, **C6** vs **A8**).

Although the activity of the compounds against some of the fungi is inferior to carbendazim, this class of compounds showed some attractive advantages, such as natural compounds or natural compound framework, simple structure, easy synthesis, low-cost as well as environmentally friendly [6,7]. Therefore, cinnamic acid esters possess great potential as new antifungal agents or promising lead compounds for development of new environmentally friendly plant fungicides. The results of this study can provide direction for the further optimal design of the structure. Furthermore, it is necessary to determine the *in vivo* activity and action mechanism of these compounds.

## Supporting information

S1 File ^1^H NMR, ^13^C NMR, LRMS and HRMS data and spectra of the compounds and their toxicity regression equations against four fungi. (PDF)
