# Supplementary material for: Bioactivity and structure-activity relationship of cinnamic acid esters and their derivatives as potential antifungal agents for plant protection
Source: PLoS One. 2017 Apr 19;12(4):e0176189. doi: 10.1371/journal.pone.0176189 (PMC5397049; doi:10.1371/journal.pone.0176189)
Supplement: S1 File — (PDF) [file pone.0176189.s001.pdf]

Supporting information

Bioactivity and Structure-Activity Relationship of Cinnamic Acid Esters and Their Derivatives as Potential Antifungal Agents for Plant Protection

Kun Zhou[1¶], Dongdong Chen[1¶], Bin Li[2], Bingyu Zhang[1], Fang Miao[2*], Le Zhou[1*]

[1]College of Chemistry & Pharmacy, Northwest A&F University, Yangling, Shaanxi, People's Republic of China

[2]College of Life Science, Northwest A&F University, Yangling, Shaanxi, People's Republic of China

*Corresponding authors

E-mail: zhoulechem@nwsuaf.edu.cn (L. Zhou); miaofangmf@163.com (F. Miao).)

¶These authors contributed equally to this work.

Contents

NMR data and physical properties of Compounds **A11-A15**, **A17**, **A18**, **A20−A23**, **B1−B5**, **B7−B12**, **C1**, **C3**, **C5**, **C9**, **C13**, **C15**, **C17**, **C19**……………………………………………………………………………………………3

^{1}H NMR and ^{13}C NMR spectra of compounds **A11-A15**, **A17**, **A18** and **A20−24**…………………………………8

^{1}H NMR and ^{13}C NMR spectra of compounds **B1−B12**………………………..…………………………………….15

^{1}H NMR and ^{13}C NMR spectra of compounds **C1−C20**…………………………………………………………...21

HRMS of **C2**, **C4**, **C6**, **C8**, **C10-C12**, **C14**, **C16**, **C18**, and **C20**……………………….………………………39

References……………………………………………………………………………………………………….45

Table……..46

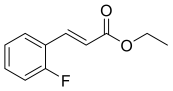

Ethyl 2-fluorocinnamate (A11). Yield: 91%; a yellow oil; ^{1}H NMR (500 MHz, $CDCl_3$): δ 7.83 (1H, d, *J* = 16.2 Hz), 7.53 (1H, 2×t, *J* = 7.5, 1.6 Hz), 7.34-7.36 (1H, m), 7.17-7.14 (1H, m), 7.07-7.11 (1H, m), 6.55 (1H, d, *J* = 16.1 Hz), 4.29 (2H, q, *J* = 7.1 Hz), 1.35 (3H, t, *J* = 7.1 Hz), matching those in literature.[1]

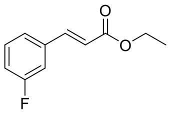

Ethyl 3-fluorocinnamate (A12). Yield: 93%; a yellow oil; ^{1}H NMR (500 MHz, $CDCl_3$): δ 7.65 (1H, d, *J* = 16.0 Hz), 7.28-7.37 (2H, m), 7.23 (1H, 2×t, *J* = 9.7, 3.8 Hz), 7.09 (1H, 2×t, *J* =8.2, 1.7 Hz), 6.44 (1H, d, *J* = 16.0 Hz), 4.29 (2H, q, *J* = 7.1 Hz), 1.35 (3H, t, *J* = 7.1 Hz, 3H) , matching those in literature.[2]

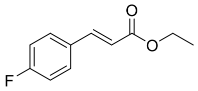

Ethyl 4-fluorocinnamate (A13). Yield: 95%; a yellow crystal; mp 30-32 °C; ^{1}H NMR (500 MHz, $CDCl_3$): δ 7.66 (1H, d, *J* = 16.0 Hz), 7.49-7.52 (2H, m), 7.09 (2H, t, *J* = 8.6 Hz), 6.37 (1H, d, *J* = 16.0 Hz), 4.28 (2H, q, *J* = 7.1 Hz), 1.35 (3H, t, *J* = 7.1 Hz), matching those in literature.[3]

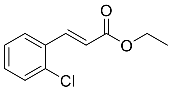

Ethyl 2-chlorocinnamate (A14). Yield: 91%; a yellow oil; ^{1}H NMR (500 MHz, $CDCl_3$): δ 8.10 (1H, d, *J* = 16.0 Hz), 7.62 (1H, dd, *J* = 7.5, 2.0 Hz), 7.41 (1H, dd, *J* = 7.5, 1.5 Hz), 7.25-7.31(2H, m), 6.44 (1H, d, *J* = 15.9 Hz), 4.30 (2H, q, *J* = 7.1 Hz), 1.36 (3H, t, *J* = 7.1 Hz), matching those in literature.[4]

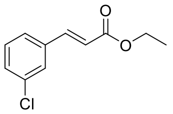

Ethyl 3-chlorocinnamate (A15). Yield : 92%; a yellow needle crystal;. mp 33-35 °C. ^{1}H NMR (500 MHz, $CDCl_3$): δ 7.62 (1H, d, *J* = 16.0 Hz), 7.50 (1H, s), 7.29-7.39 (3H, m), 6.44 (1H, d, *J* = 16.0 Hz), 4.28 (2H, q, *J* = 7.1 Hz), 1.35 (3H, t, *J* = 7.1 Hz), agreement with those reported in literature.[5]

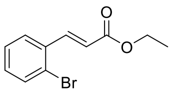

Ethyl 2-Bromocinnamate (A17). Yield: 90%; a yellow oil; ^{1}H NMR (500 MHz, $CDCl_3$): δ 8.06 (1H, d, *J* = 15.9 Hz), 7.58-7.61 (2H, m), 7.33 (1H, t, *J* =7.4 Hz), 7.23 (1H, 2×t, *J* = 7.8, 1.5 Hz), 6.40 (1H, d, *J* = 15.9 Hz), 4.30 (2H, q, *J* = 7.1 Hz), 1.36 (3H, t, *J* = 7.1 Hz) , agreement with those reported in literature.[1]

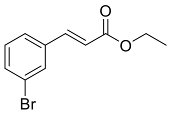

Ethyl 3-Bromocinnamate (A18). Yield: 94%; a yellow needle crystal; mp 36-37 °C (34-36 °C[27]); ^{1}H NMR (500 MHz, $CDCl_3$): δ 7.65 (1H, s), 7.60 (1H, d, *J* = 16.0 Hz), 7.49 (1H, d, *J* = 7.8 Hz), 7.43 (1H, d, *J* = 7.7 Hz), 7.26 (1H, q, *J*

= 7.7 Hz), 6.43 (1H, d, J = 16.0 Hz), 4.28 (2H, q, J = 7.1 Hz), 1.34 (3H, t, J = 7.1 Hz), agreement with those reported in literature.[6]

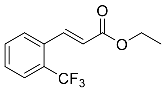

Ethyl 2-trifluoromethylcinnamate (A20). Yield : 90%; a yellow oil; ^{1}H NMR (500 MHz, $CDCl_3$): δ 8.07 (1H, d, J = 15.8 Hz), 7.71 (2H, d, J = 8.1 Hz), 7.58 (1H, t, J = 7.6 Hz), 7.49 (1H, t, J = 7.6 Hz), 6.42 (1H, d, J = 15.7 Hz), 4.30 (2H, q, J = 7.1 Hz), 1.36 (3H, t, J = 7.1 Hz), agreement with those reported in literature.[1]

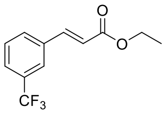

Ethyl 3-trifluoromethylcinnamate (A21). Yield: 88%; a yellow powder; mp 37-38 °C. ^{1}H NMR (500 MHz, $CDCl_3$): δ 7.76 (1H, s), 7.69 (1H, d, J = 16.0 Hz), 7.68 (1H, d, J = 7.7 Hz), 7.62 (1H, d, J = 7.7 Hz), 7.50 (1H, t, J =7.7 Hz), 6.52 (1H, d, J = 16.0 Hz), 4.30 (2H, q, J = 7.1 Hz), 1.36 (3H, t, J = 7.1 Hz); ^{13}C NMR (125 MHz, $CDCl_3$): δ 166.4, 142.7, 135.3, 131.4 (q, J = 32.5 Hz), 131.0, 129.4, 126.6 (q, J = 3.6 Hz), 124.6 (q, J = 3.8 Hz), 123.8 (q, J = 271.4 Hz), 120.3, 60.7, 14.2. The spectra data are agreement with those reported in literature.[2]

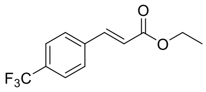

Ethyl 4-trifluoromethylcinnamate (A22). Yield: 88%; a white powder; mp 31-33 °C. ^{1}H NMR (500 MHz, $CDCl_3$): δ 7.71 (1H, d, J = 16.0 Hz), 7.61-7.65 (4H, m), 6.52 (1H, d, J = 16.0 Hz), 4.30 (2H, q, J = 7.1 Hz), 1.36 (3H, t, J = 7.1 Hz, 3H), agreement with those reported in literature.[3]

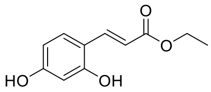

Ethyl 2,4-dihydroxycinnamate (A23). Yield: 63%; a white powder; mp 74-75℃; ^{1}H NMR (500 MHz, DMSO-d_6,): δ 10.1 (1H, s, OH), 9.8 (1H, s, OH), 7.77 (1H, d, J = 16.0 Hz), 7.41 (1H, d, J = 8.5 Hz), 6.34-6.38 (2H, m), 6.27 (1H, dd, J = 8.5, 2.3 Hz), 4.27 (2H, q, J = 7.1 Hz), 1.34 (3H, t, J = 7.1 Hz), consistent with those in literature.[7]

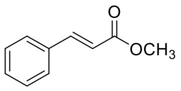

Methyl cinnamate (B1). Yield: 97%; white needle crystal; mp 36-37 °C; ^{1}H NMR (500 MHz, $CDCl_3$): δ 7.69 (1H, d, J = 16.0 Hz), 7.51–7.50 (2H, m), 7.38–7.37 (m, 3H), 6.44 (1H, d, J = 16.0 Hz), 3.80 (3H, s), matching those reported in literature.[1]

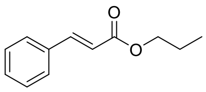

n-Propyl cinnamate (B2). Yield: 96%; a colorless oil; ^{1}H NMR (500 MHz, $CDCl_3$): δ 7.69 (1H, d, J = 16.0 Hz), 7.53–7.51 (2H, m), 7.38–7.37 (3H, m), 6.45 (1H, d, J = 16.0 Hz), 4.17 (2H, t, J = 6.7 Hz), 1.73 (2H, m), 1.00 (3H, t, J = 7.4 Hz), agreement with those reported in literature.[8]

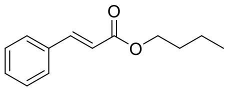

n-Butyl cinnamate (B3). Yield: 95%; a colorless oil; ^{1}H NMR (500 MHz, $CDCl_3$): δ 7.73 (1H, d, J = 16.0 Hz), 7.58–7.56 (2H, m), 7.43–7.42 (3H, m), 6.49 (1H, d, J = 16.0 Hz), 4.26 (2H, t, J = 6.7 Hz), 1.77–1.71 (2H, m), 1.51–1.47 (2H, m), 1.02 (3H, t, J = 7.4 Hz), agreement with those reported in literature.[8]

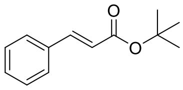

t-Butyl cinnamate (B4). Yield: 78%; a colorless oil; ^{1}H NMR (500 MHz, $CDCl_3$): δ 7.64 (1H, d, J = 16.0 Hz), 7.56–7.55 (2H, m), 7.43–7.40 (3H, m), 6.42 (1H, d, J = 16.0 Hz), 1.58 (9H, s), agreement with those reported in literature.[9]

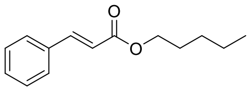

n-Amyl cinnamate (B5). Yield: 85.3%; a colorless oil; ^{1}H NMR (500 MHz, $CDCl_3$): δ 7.73 (1H, d, J = 16.0 Hz), 7.59–7.57 (2H, m), 7.44–7.42 (3H, m), 6.49 (1H, d, J = 16.0 Hz), 4.25 (2H, t, J = 6.8 Hz), 1.79–1.73 (2H, m), 1.45–1.42 (4H, m), 0.98 (t, J = 7.0 Hz, 3H), matching those reported in literature.[10]

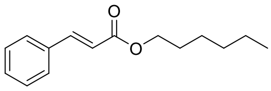

n-Hexyl cinnamate (B7). Yield: 86%; a colorless oil; ^{1}H NMR (500 MHz, $CDCl_3$): δ 7.73 (1H, d, J = 16.0 Hz), 7.58–7.57 (2H, m), 7.44–7.42 (3H, m), 6.49 (1H, d, J = 16.0 Hz), 4.25 (2H, t, J = 6.8 Hz), 1.78–1.72 (2H, m), 1.47–1.44 (2H, m), 1.39–1.37 (4H, m), 0.95 (3H, t, J = 6.8 Hz), consistent with those reported in literature.[11]

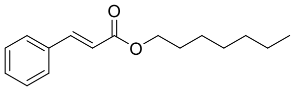

n-Heptyl cinnamate (B8). Yield: 80%; a colorless oil; ^{1}H NMR (500 MHz, $CDCl_3$): δ 7.73 (1H, d, J = 16.0 Hz), 7.59–7.57 (2H, m), 7.44–7.42 (3H, m), 6.49 (1H, d, J = 16.0 Hz), 4.25 (2H, t, J = 6.8 Hz), 1.78–1.72 (2H, m), 1.41–1.30 (8H, m), 0.94 (3H, t, J = 6.9 Hz), matching those reported in literature.[12]

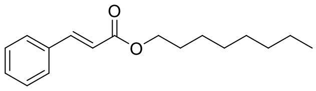

n-Octyl cinnamate (B9). Yield: 77.5%; a colorless oil; ^{1}H NMR (500 MHz, $CDCl_3$): δ 7.73 (1H, d, J = 16.0 Hz), 7.59–7.57 (2H, m), 7.44–7.42 (3H, m), 6.49 (1H, d, J = 16.0 Hz), 4.25 (2H, t, J = 6.8 Hz), 1.77–1.72 (2H, m), 1.45–1.40 (2H, m), 1.36–1.30 (8H, m), 0.94 (3H, t, J = 7.0 Hz), matching those reported in literature.[13]

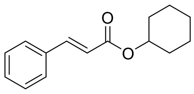

Cyclohexyl cinnamate (B10). Yield: 73%; a colorless oil; ^{1}H NMR (500 MHz, $CDCl_3$): δ 7.71 (1H, d, J = 16.0 Hz), 7.58–7.56 (2H, m), 7.43–7.41 (3H, m), 6.48 (1H, d, J = 16.0 Hz), 4.97–4.91 (1H, m), 1.99–1.95 (2H, m), 1.83–1.80 (2H,

m), 1.63–1.31 (6H, m), matching those reported in literature.[9]

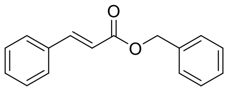

Benzyl cinnamate (B11). Yield: 76%; a white needle crystal; mp 38.7-39.2 °C (33-35 °C[34]); ^{1}H NMR (500 MHz, $CDCl_3$) δ 7.79 (1H, d, J = 16.0 Hz), 7.58–7.56 (2H, m), 7.49–7.39 (8H, m), 6.54 (1H, d, J = 16.0 Hz), 5.31 (2H, s), matching those reported in literature.[14]

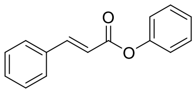

Phenyl cinnamate (B12). Yield: 73%; a white needle crystal; mp 76-78 °C (72-75 °C[34]); ^{1}H NMR (500 MHz, $CDCl_3$): δ 7.87 (1H, d, J = 16.0 Hz), 7.59–7.57 (2H, m), 7.42–7.39 (5H, m), 7.24 (1H, t, J = 7.7 Hz), 7.17 (2H, d, J = 7.7 Hz), 6.63 (1H, d, J = 16.0 Hz), consistent with those reported in literature.[14]

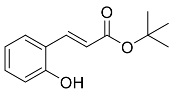

t-Butyl 2-hydroxycinnamate (C1). Yield: 70%; a yellow powder; mp 86.5-87.5 °C; ^{1}H NMR (500 MHz, $CDCl_3$): δ 8.01 (1H, d, J = 16.2 Hz), 7.45 (1H, dd, J = 7.7, 1.4 Hz), 7.35 (1H, s, OH), 7.22 (1H, 2×t, J = 8.0, 1.6 Hz), 6.85–6.89 (2H, m), 6.60 (1H, d, J = 16.2 Hz), 1.55 (9H, s), agreement with those in literature.[15]

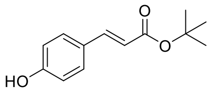

t-Butyl 4-hydroxycinnamate (C3). Yield: 77%; a yellow powder; mp 98.0-98.8 °C; ^{1}H NMR (500 MHz, $CDCl_3$): δ 7.54 (1H, d, J = 15.9 Hz), 7.39 (2H, d, J = 8.6 Hz), 6.86 (2H, d, J = 8.6 Hz), 6.51 (1H, s, OH), 6.24 (1H, d, J = 15.9 Hz), 1.53 (9H, s), agreement with those in the literature.[16]

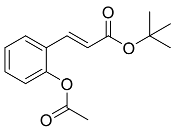

t-Butyl 2-acetoxyl cinnamate (C5). Yield: 97%; a yellow oil; ^{1}H NMR (500 MHz, $CDCl_3$): δ 7.68 (1H, d, J = 16.1 Hz), 7.63 (1H, dd, J = 7.8, 1.4 Hz), 7.37 (1H, 2×t, J = 7.9, 1.5 Hz), 7.25 (1H, t, J = 7.5 Hz), 7.11 (1H, dd, J = 8.1, 0.9 Hz), 6.39 (1H, d, J = 16.0 Hz), 2.35 (3H, s), 1.52 (9H, s), agreement with those in the literature.[17]

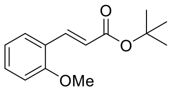

t-Butyl 2-methoxycinnamate (C7). Yield: 90%; a faint yellow oil; ^{1}H NMR (500 MHz, $CDCl_3$): δ 7.92 (1H, d, J = 16.1 Hz), 7.50 (1H, dd, J = 7.6, 1.1 Hz), 7.34 (1H, m), 6.89-6.96 (2H, m), 6.53 (1H, d, J = 16.1 Hz), 3.87 (3H, s), 1.53 (9H, s), agreement with those reported in literature.[18]

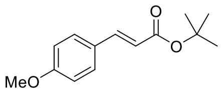

t-Butyl 4-methoxycinnamate (C9). Yield: 89%; a yellow oil; ^{1}H NMR (500 MHz, $CDCl_3$): δ 7.54 (1H, d, J = 15.9 Hz), 7.46 (2H, dd, J = 8.8, 1.9 Hz), 6.88 (2H, d, J = 8.8 Hz), 6.24 (1H, d, J = 15.9 Hz), 3.82 (3H, s), 1.52 (9H, s); ^{13}C NMR (125 MHz, $CDCl_3$): δ 166.7, 161.2, 143.2, 129.6, 127.4, 117.7, 114.3, 80.2, 55.3, 28.3. The spectra data are consistent with those in literature.[18]

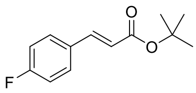

t-Butyl 4-fluorocinnamate (C13). Yield: 93%; a yellow oil; ^{1}H NMR (500 MHz, $CDCl_3$): δ 7.55 (1H, d, J = 16.1 Hz), 7.50-7.48 (2H, m), 7.06 (2H, t, J = 8.6 Hz), 6.29 (1H, d, J = 16.0 Hz), 1.53 (9H, s); ^{13}C NMR (125 MHz, $CDCl_3$): δ 166.2, 163.7 (d, J = 250.6 Hz), 142.2, 130.9 (d, J = 3.5 Hz), 129.8 (d, J = 8.7 Hz), 120.0 (d, J = 1.9 Hz), 115.9 (d, J = 21.8 Hz), 80.6, 28.2. The spectra data are consistent with those in literature.[19]

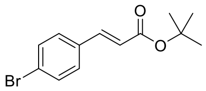

t-Butyl 4-bromocinnamate (C15). Yield: 93%; a white powder; mp 63.5-64.3 °C (60-62 °C[36]); ^{1}H NMR (500 MHz, DMSO-d_6): δ 7.66 (2H, d-like, J = 8.5 Hz), 7.60 (2H, d-like, J = 8.5 Hz), 7.54 (1H, d, J = 16.0 Hz), 6.30 (1H, d, J = 16.0 Hz), 1.48 (9H, s); ^{13}C NMR (125 MHz, $CDCl_3$): δ 166.0, 142.2, 133.6, 132.1, 129.4, 124.2, 120.9, 80.7, 28.2. The spectra data are consistent with those in literature.[19]

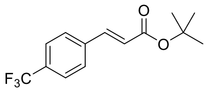

t-Butyl 4-trifluoromethylcinnamate (C17). Yield: 92%; a white powder; mp 35.9-36.2 °C; ^{1}H NMR (500 MHz, $CDCl_3$): δ7.64-7.57 (5H, m), 6.44 (1H, d, J = 16.1 Hz), 1.54 (9H, s); ^{13}C NMR (125 MHz, $CDCl_3$): δ 165.7, 141.7, 138.1, 131.5 (q, J =32.6 Hz), 128.0, 126.1 (q, J =270.5 Hz), 125.8 (q, J =3.8 Hz), 122.8, 81.0, 28.2, matching those in literature.[19]

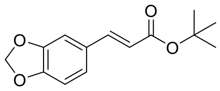

t-Butyl 3,4-methylenedioxy cinnamate (C19). Yield: 88%; a white powder; mp 73-74 °C (73-76 °C[37]); ^{1}H NMR (500 MHz, $CDCl_3$): δ 7.49 (1H, d, J = 15.9 Hz), 7.01 (1H, d, J = 1.5 Hz), 6.97 (1H, dd, J = 8.0, 1.5 Hz), 6.79 (1H, d, J = 8.0 Hz) 6.19 (1H, d, J= 15.9 Hz), 5.99 (2H, s), 1.52 (9H, s); ^{13}C NMR (125 MHz, $CDCl_3$): δ 166.5, 149.3, 148.3, 143.3, 129.1, 124.1, 118.2, 108.5, 106.5, 101.5, 80.4, 28.2. The spectra data match those in literature.[20]

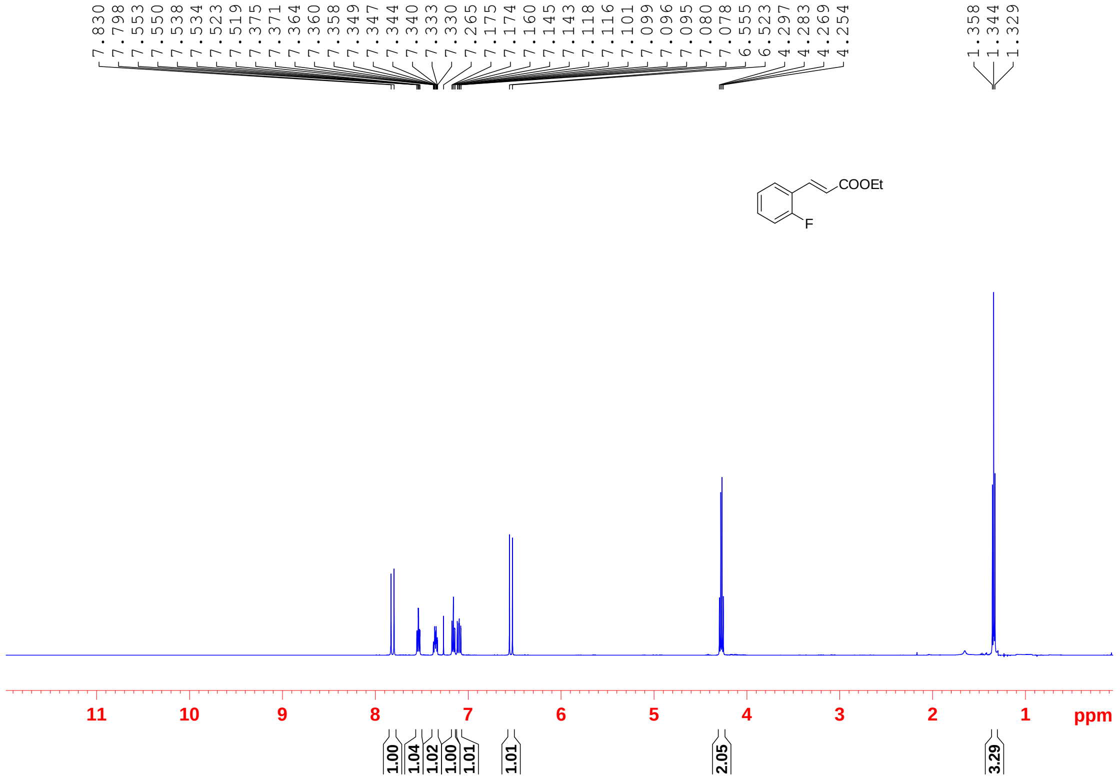

^{1}H NMR of compound A11

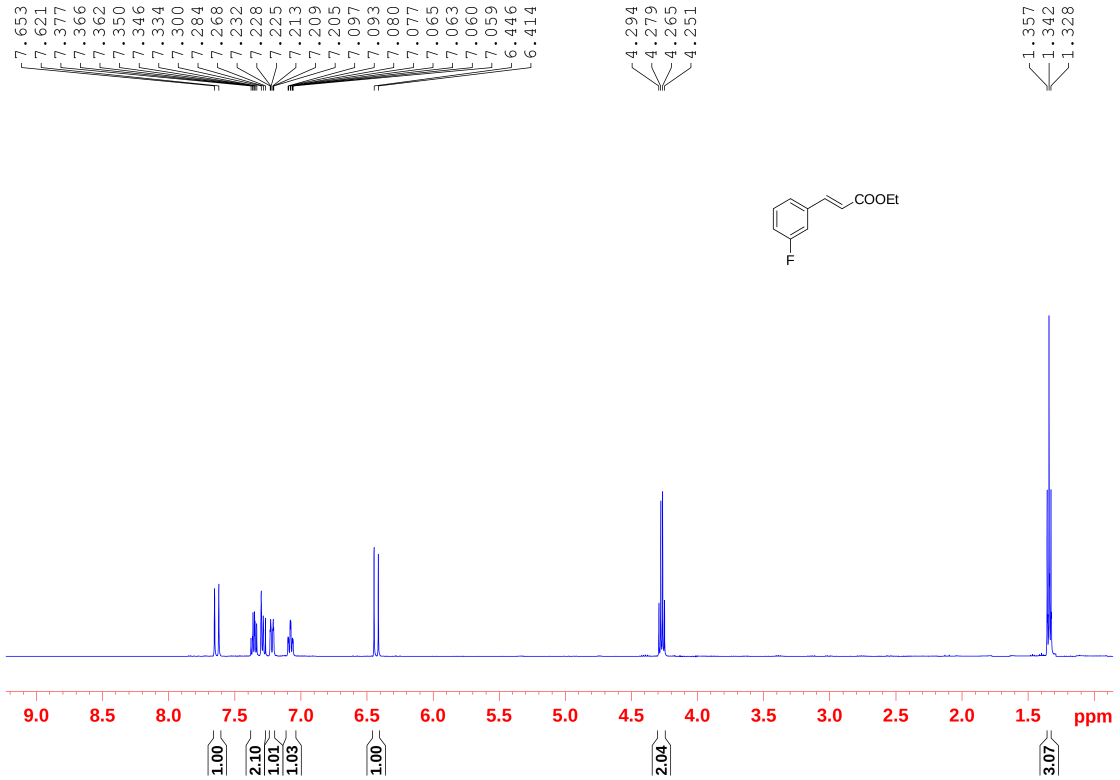

^{1}H NMR of compound A12

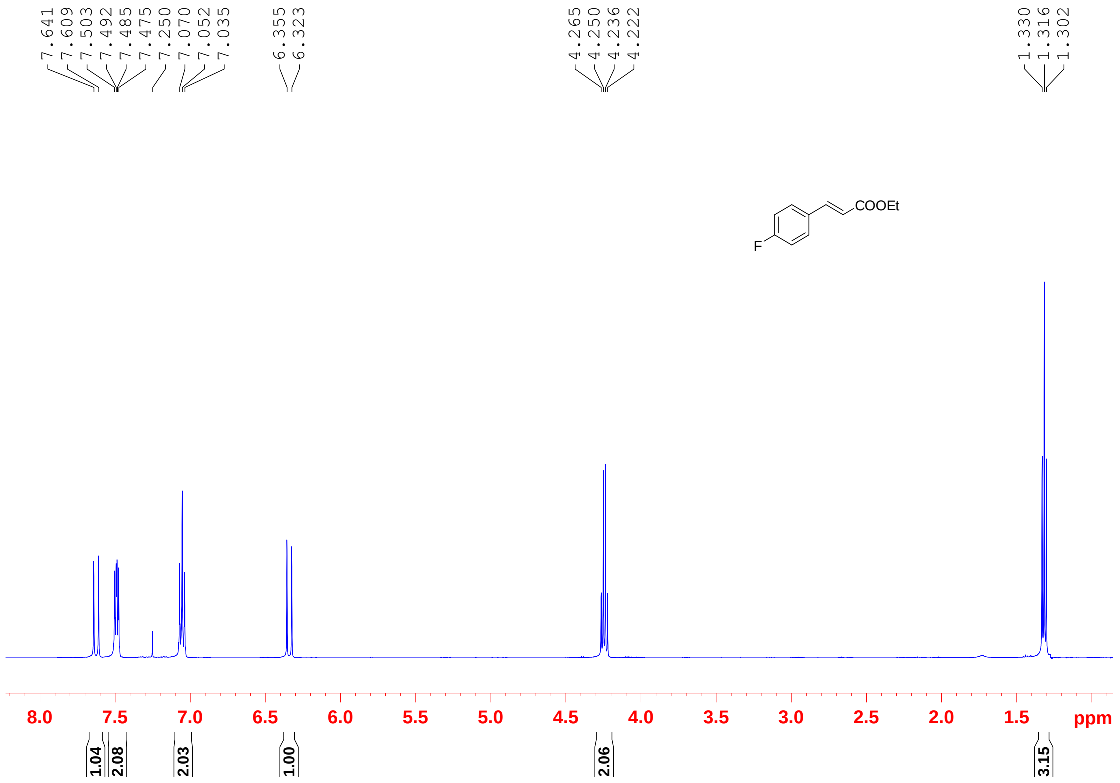

^{1}H NMR of compound A13

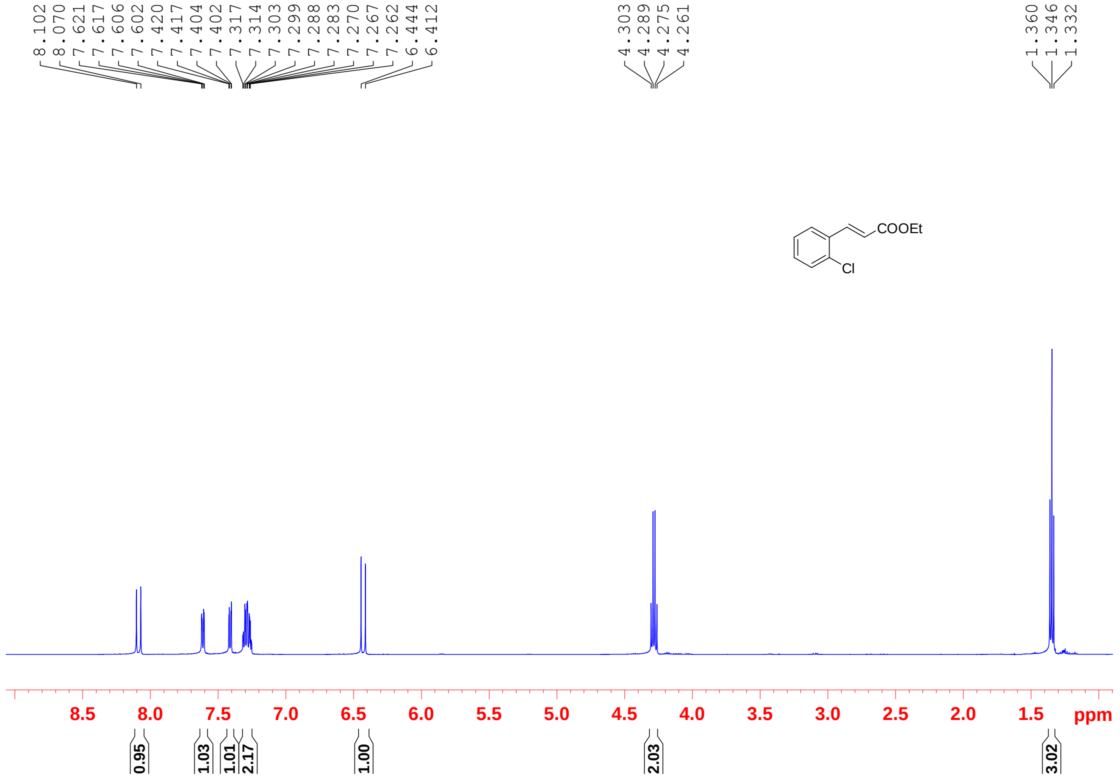

^{1}H NMR of compound A14

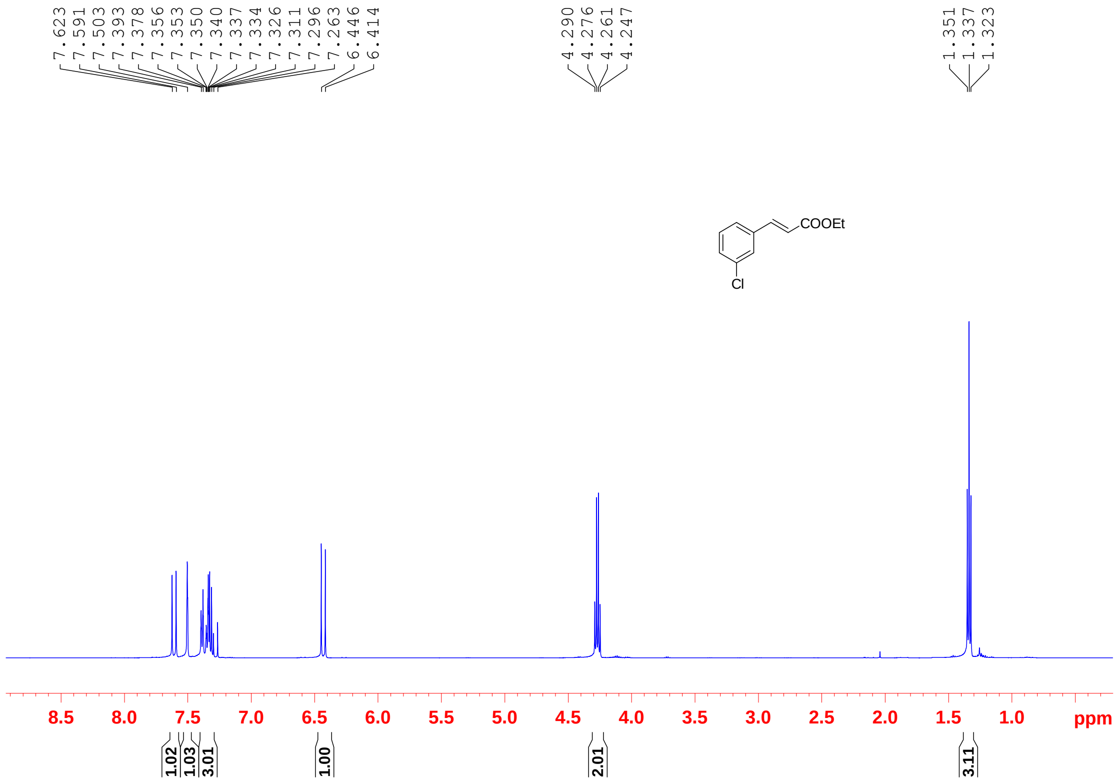

^{1}H NMR of compound A15

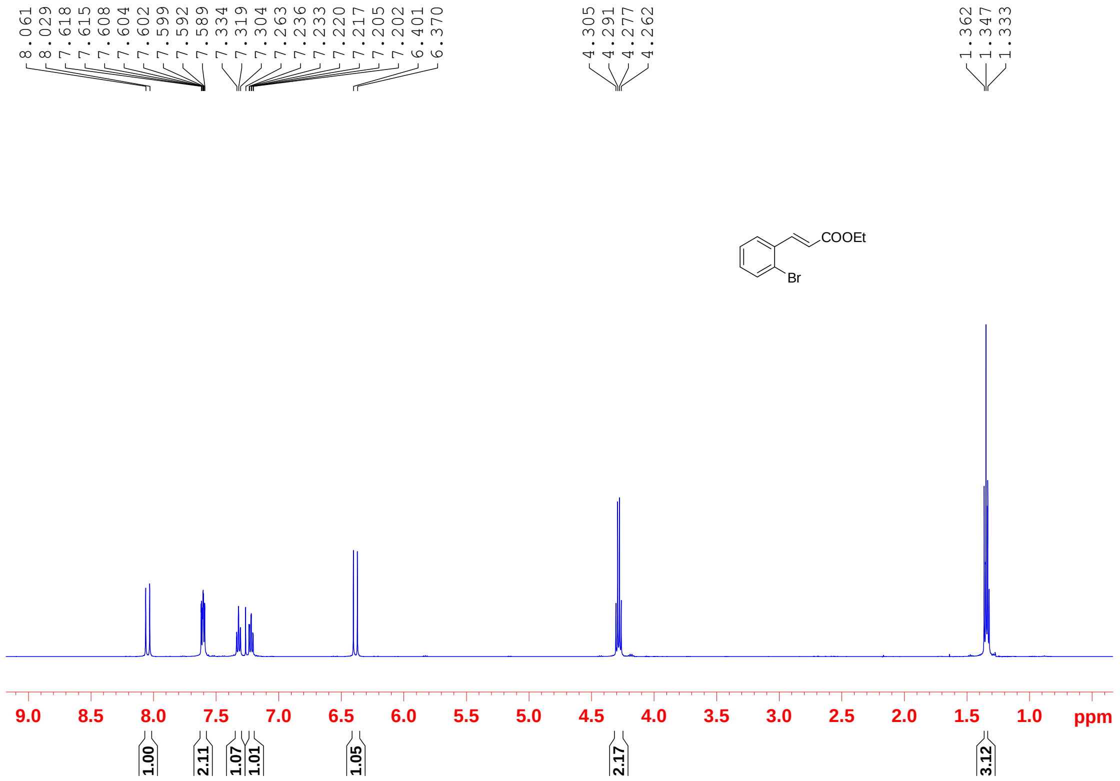

^{1}H NMR of compound A17

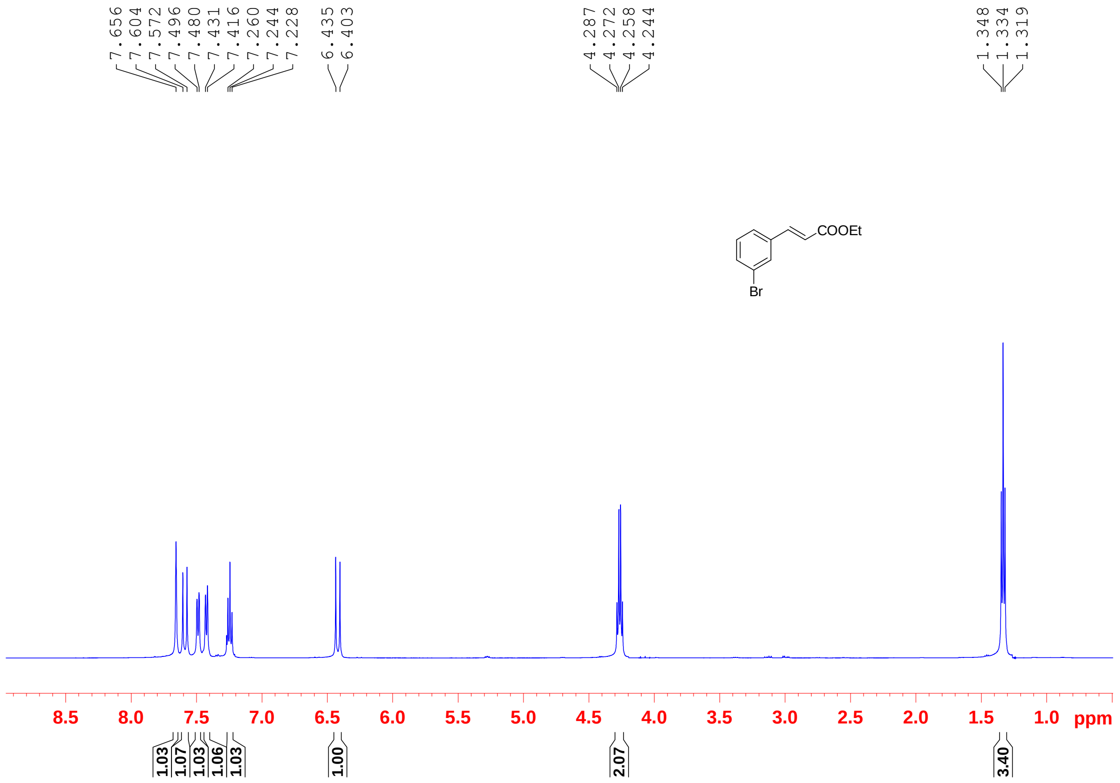

^{1}H NMR of compound A18

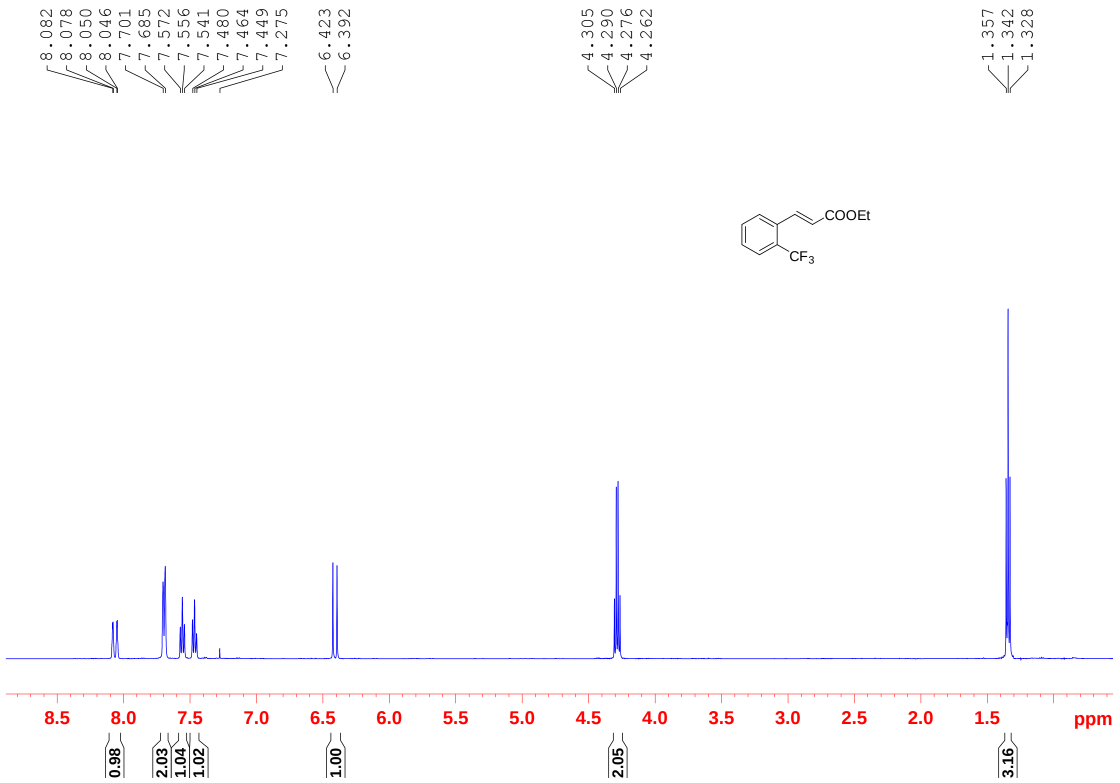

^{1}H NMR of compound A20

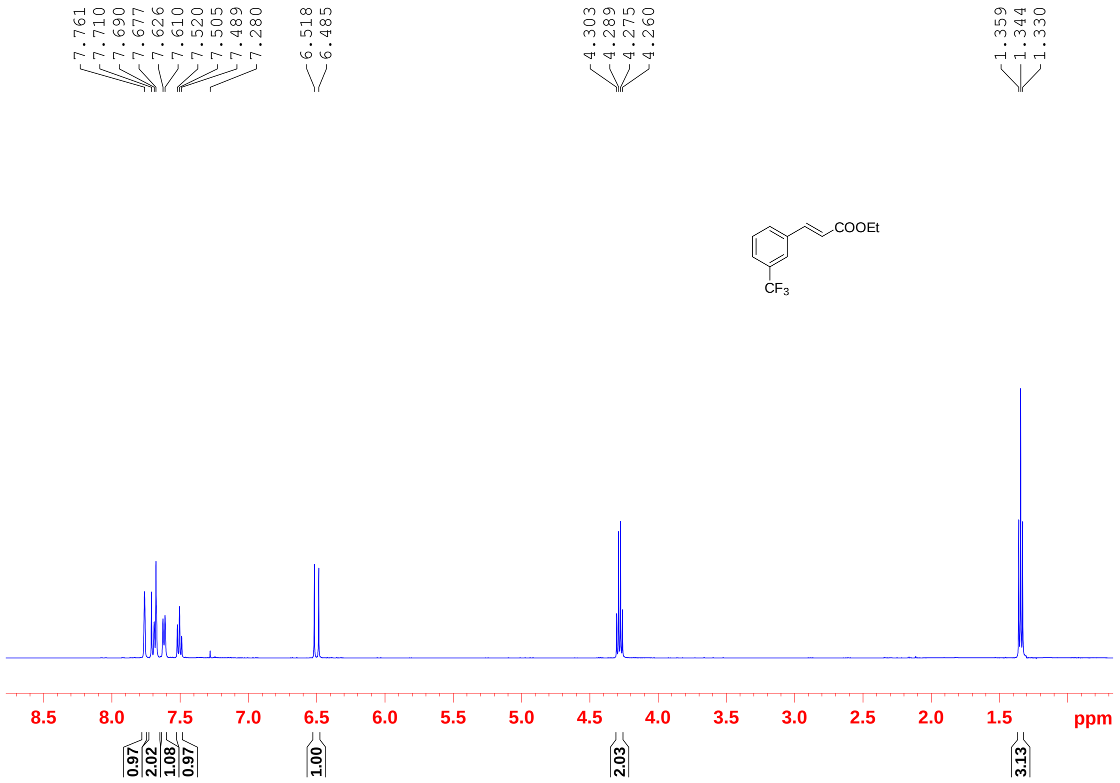

^{1}H NMR of compound A21

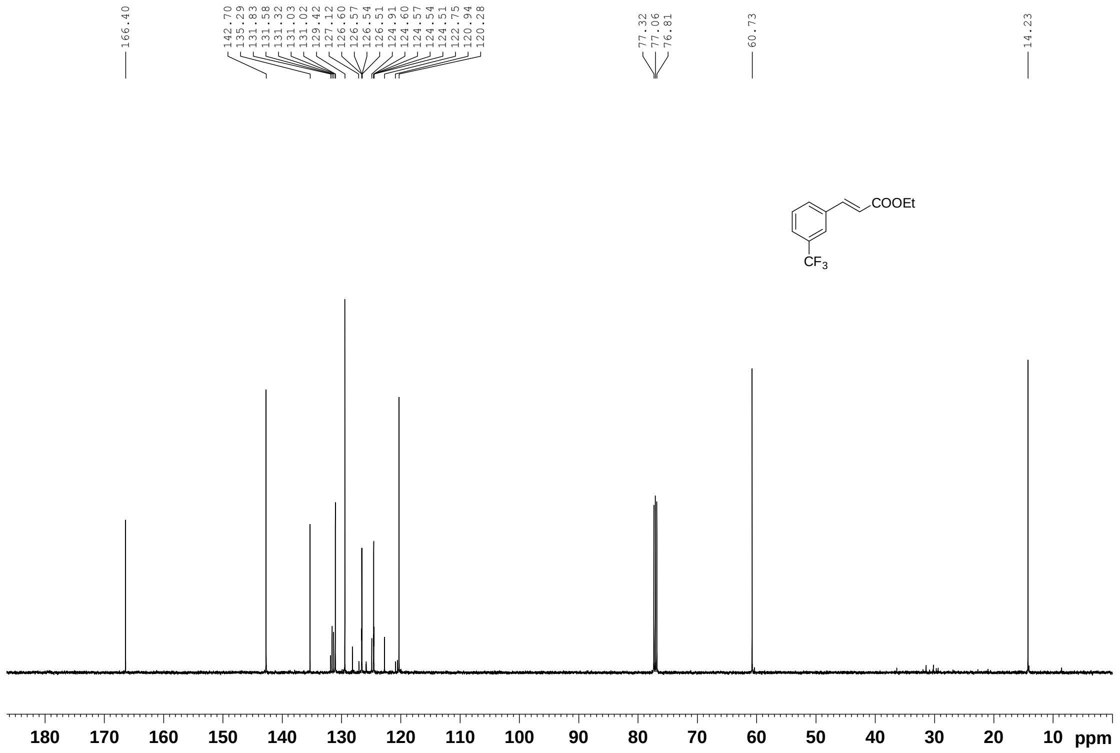

^{13}C NMR of compound A21

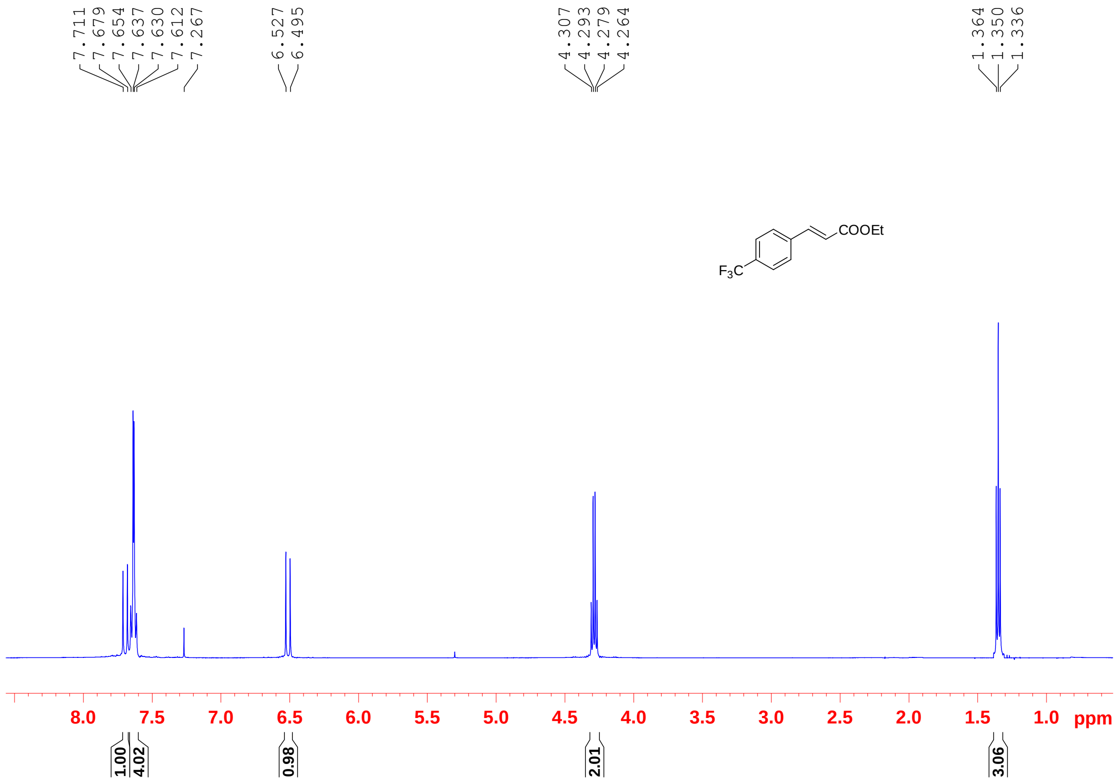

^{1}H NMR of compound A22

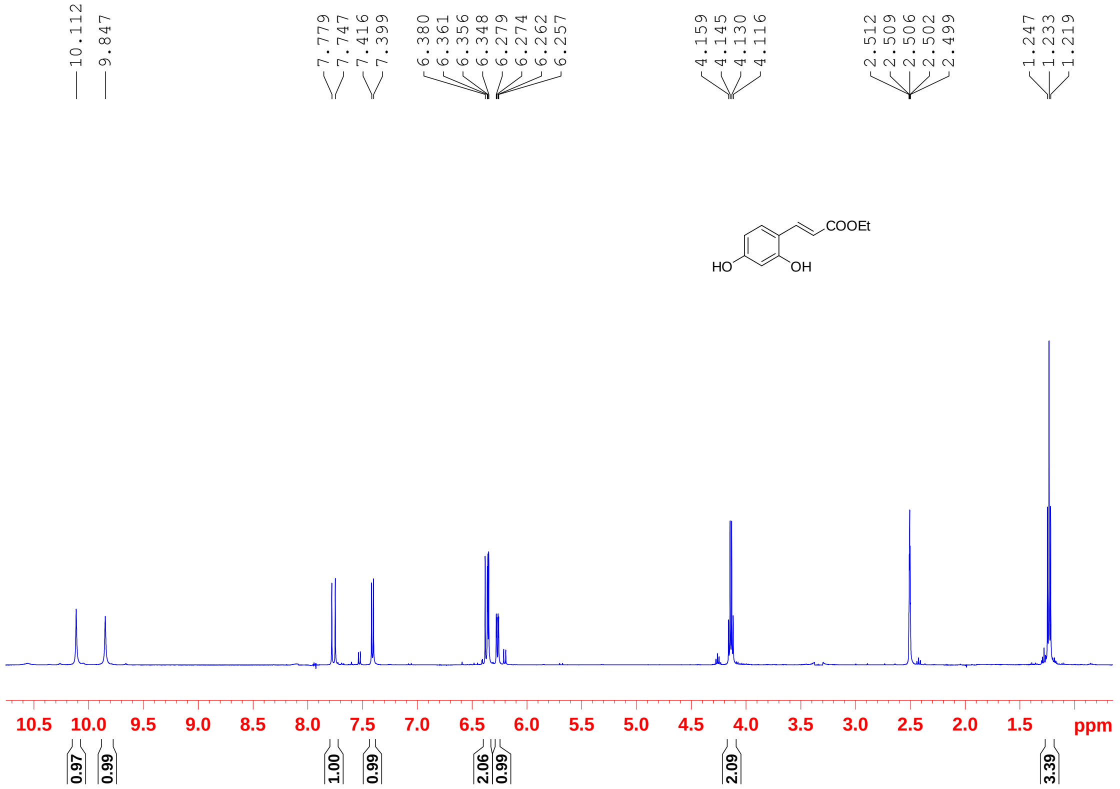

^{1}H NMR of compound A23

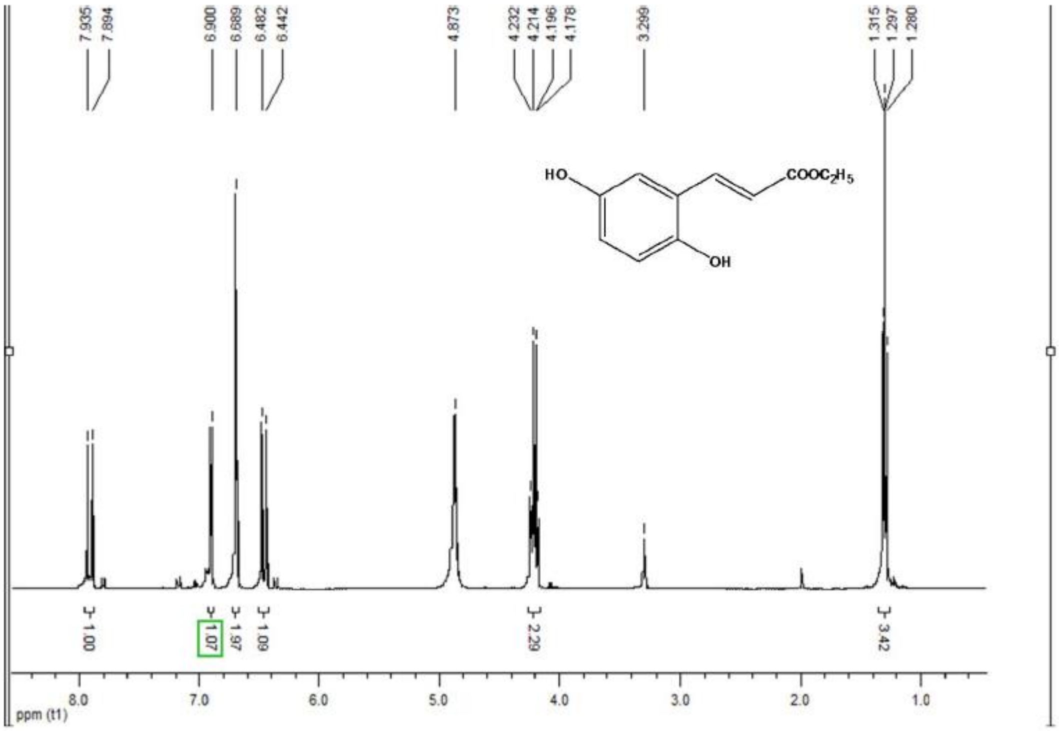

^{1}H NMR of compound A24

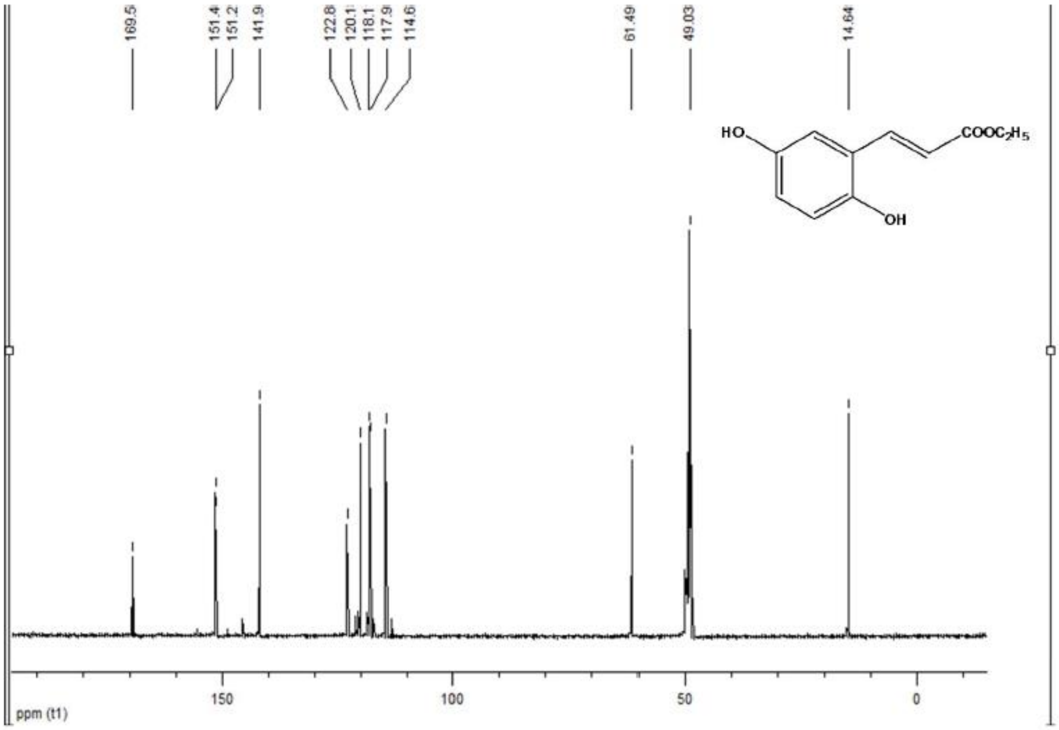

^{13}C NMR of compound A24

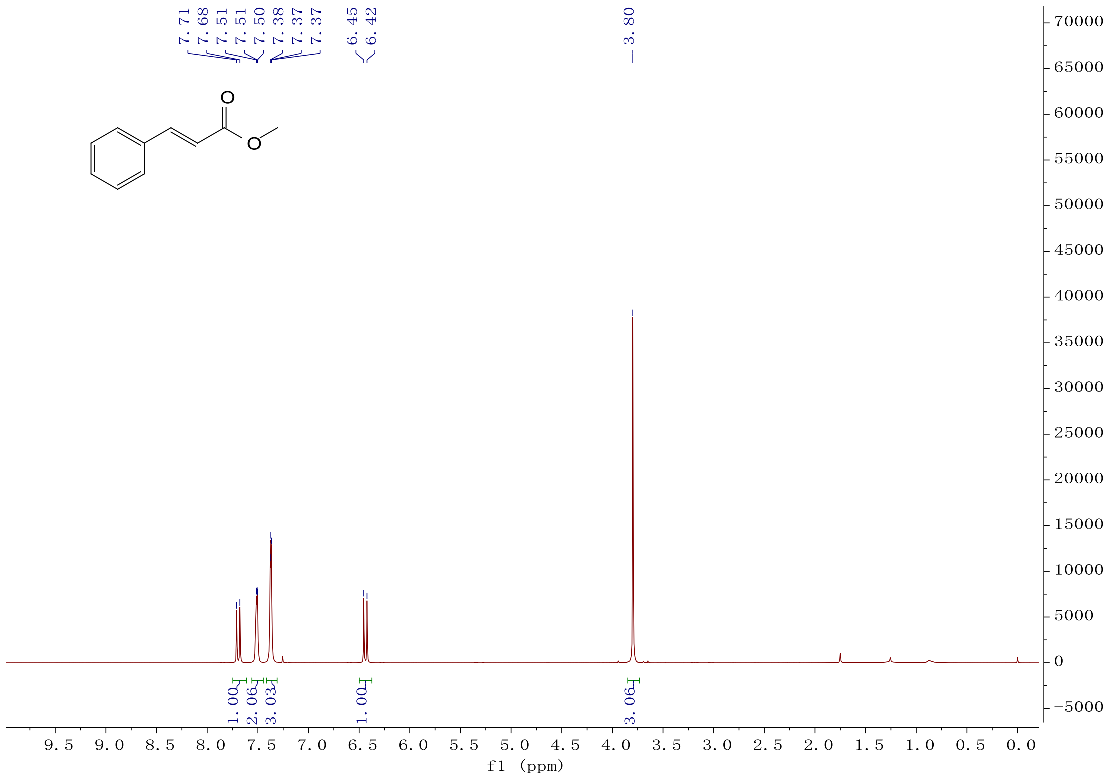

^{1}H NMR of compound B1

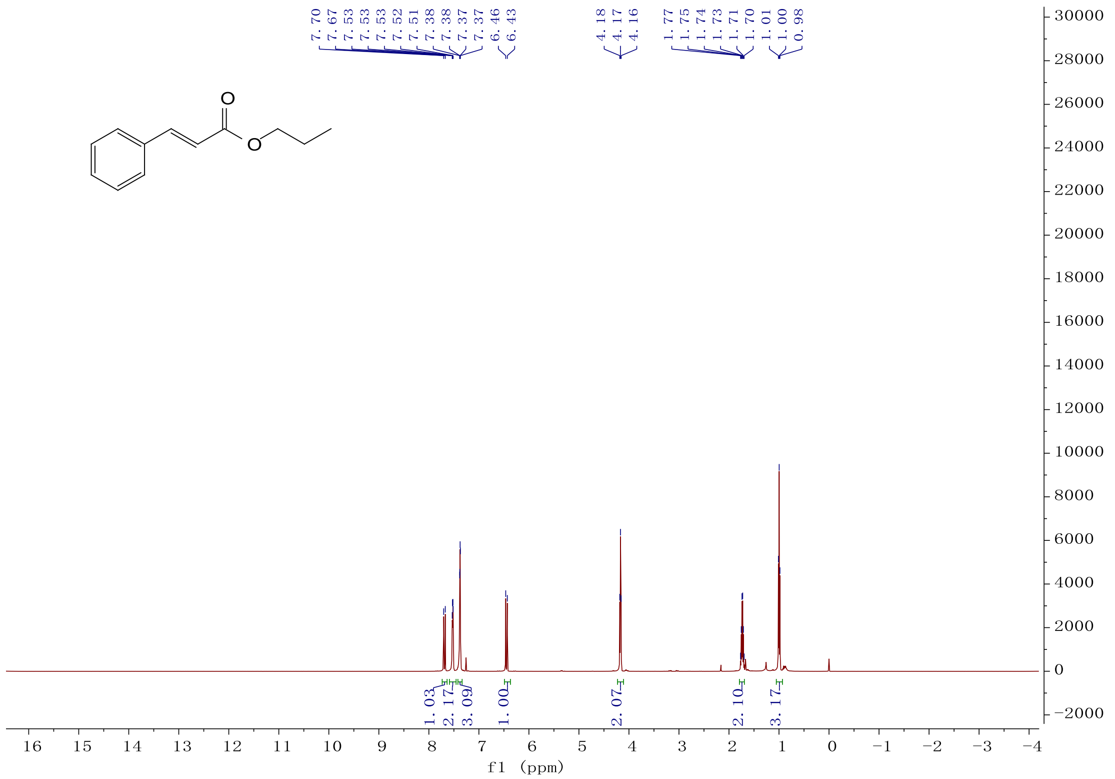

^{1}H NMR of compound B2

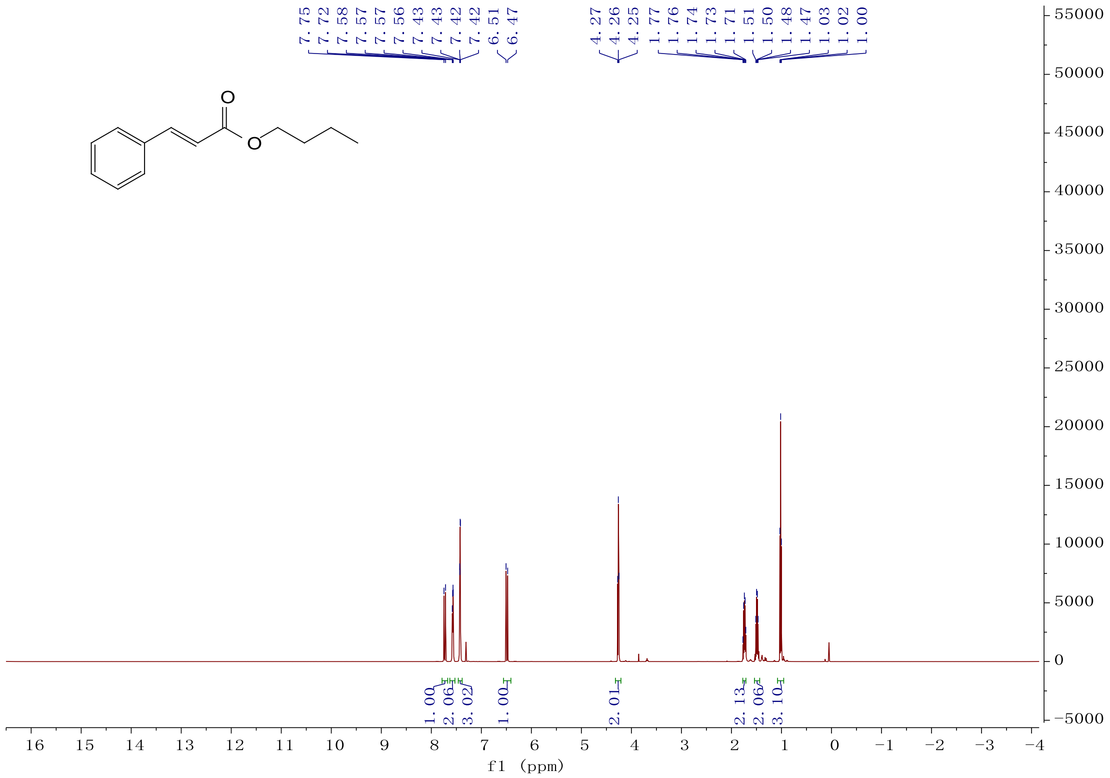

^{1}H NMR of compound B3

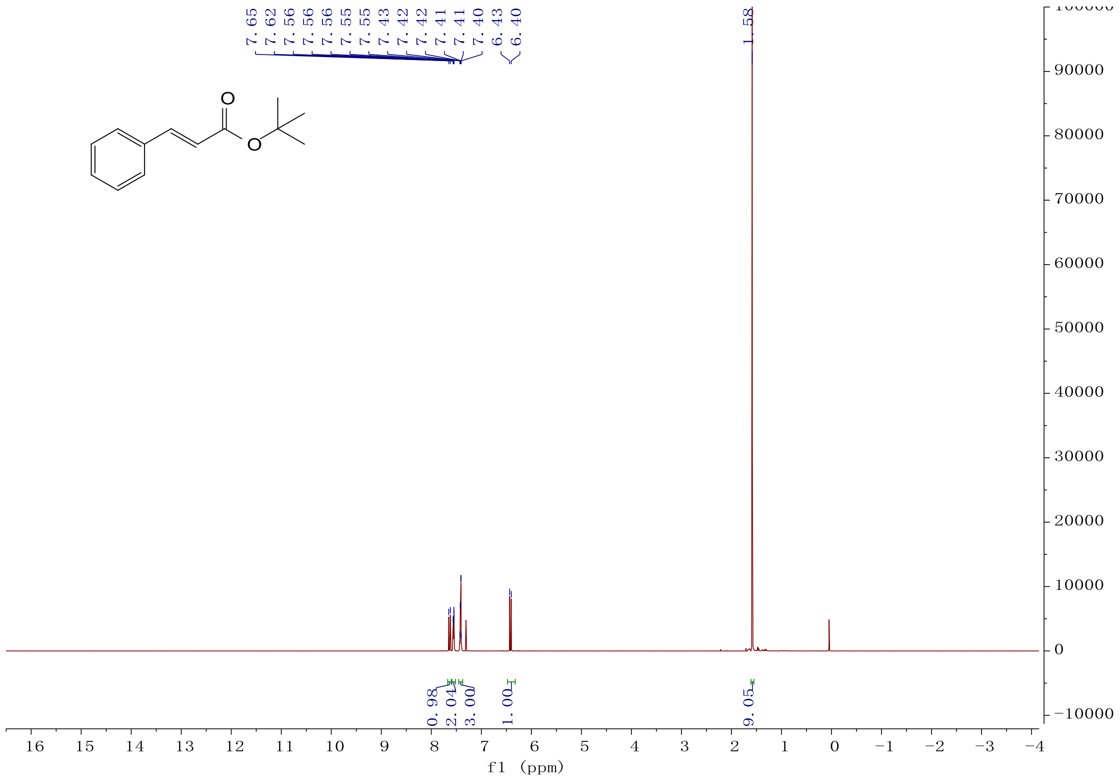

^{1}H NMR of compound B4

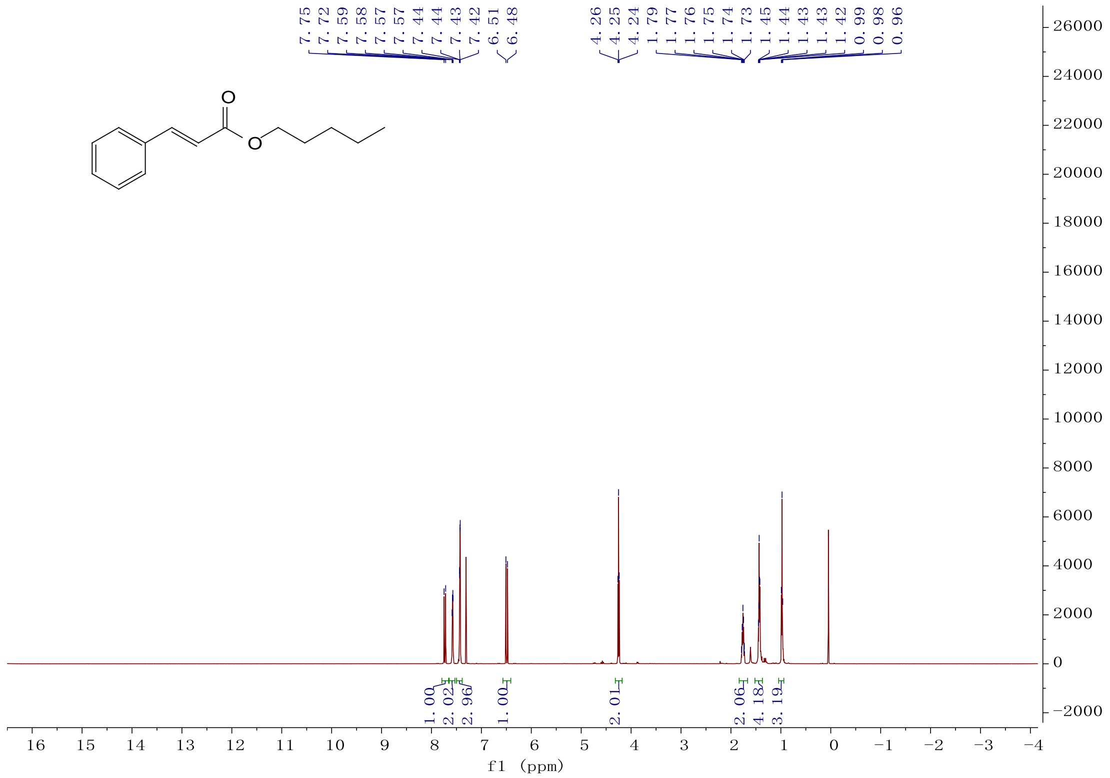

^{1}H NMR of compound B5

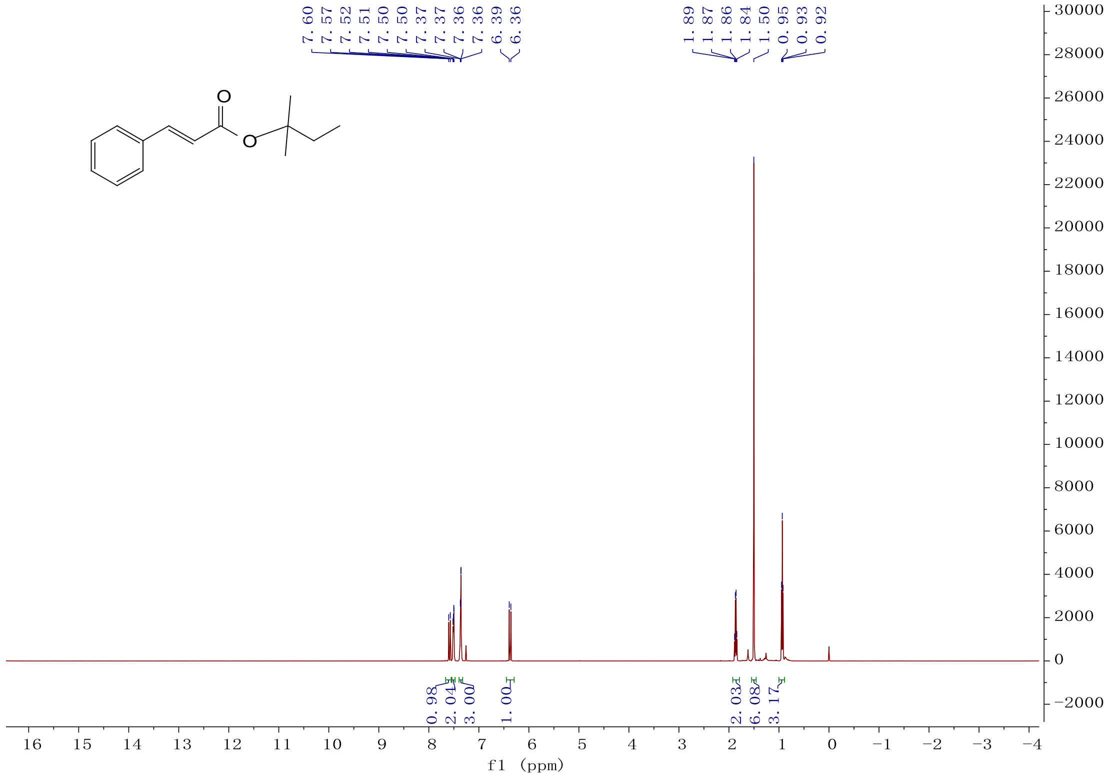

^{1}H NMR of compound B6

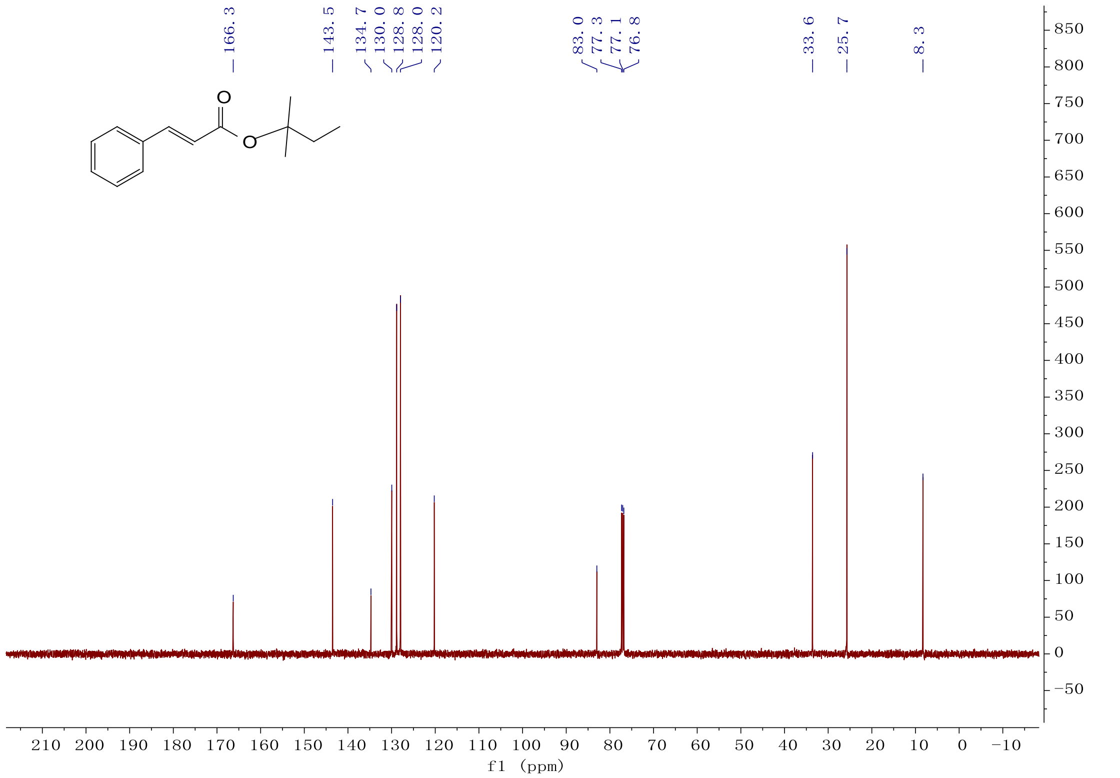

^{13}C NMR of compound B6

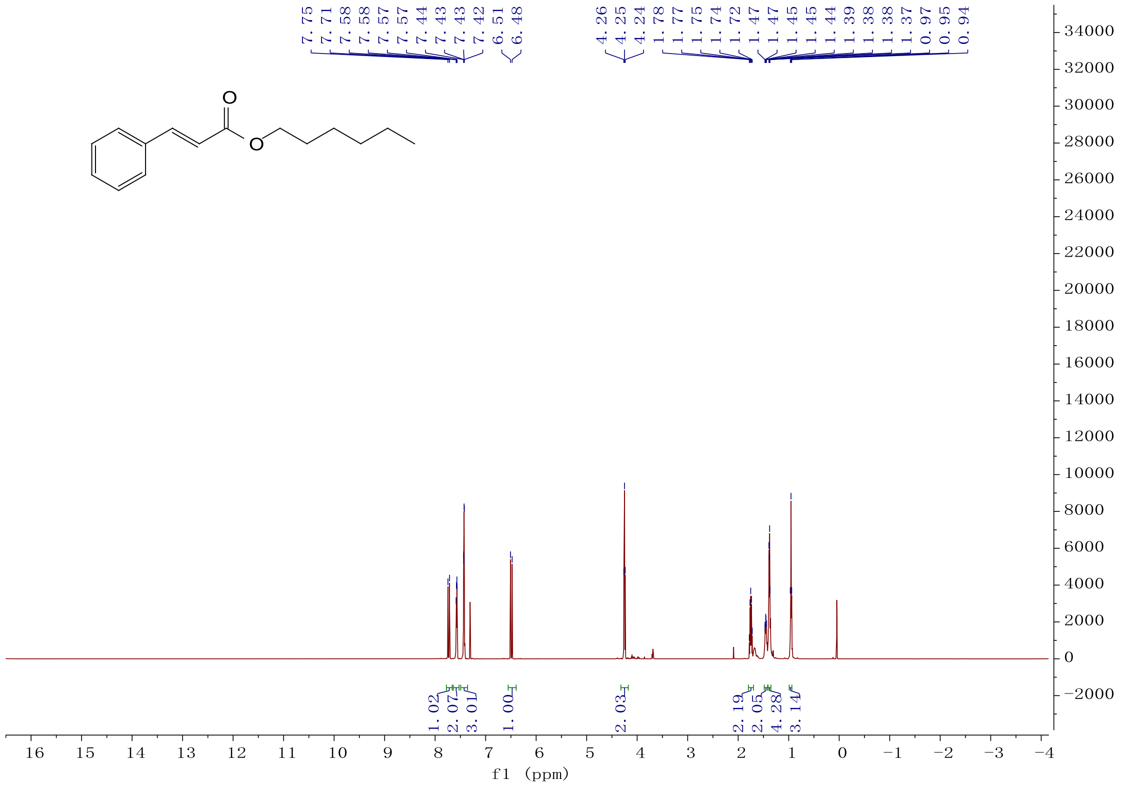

^{1}H NMR of compound B7

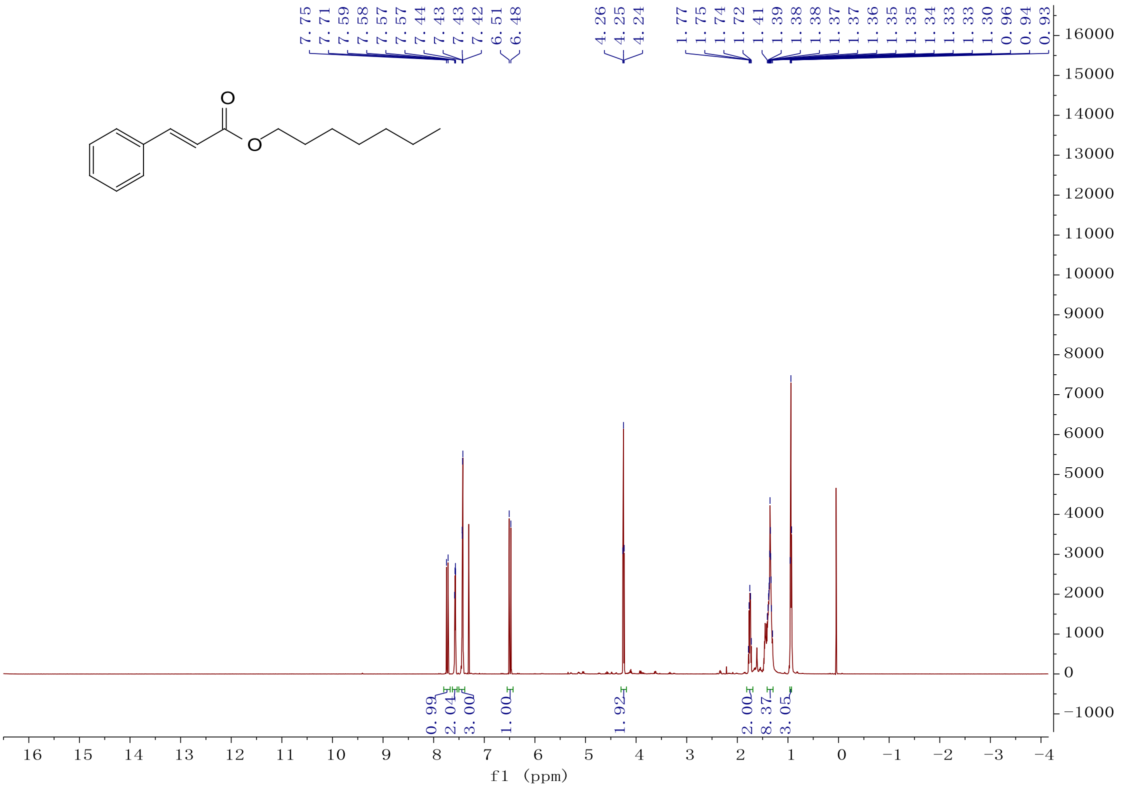

^{1}H NMR of compound B8

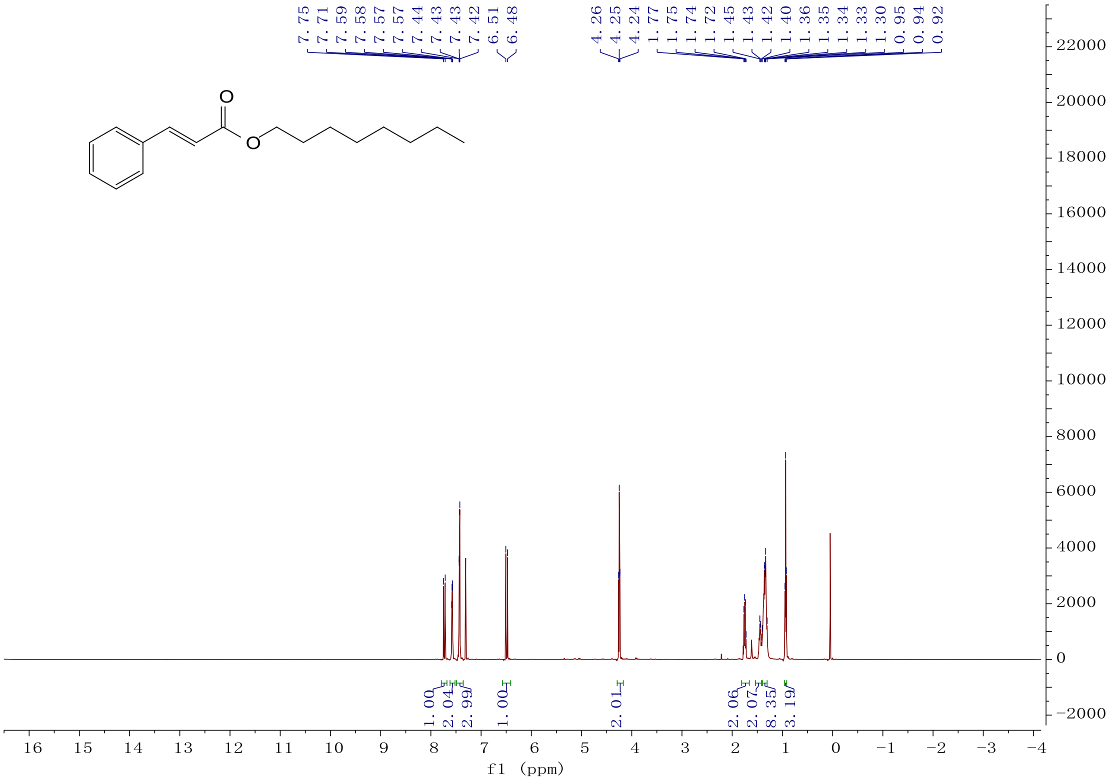

^{1}H NMR of compound B9

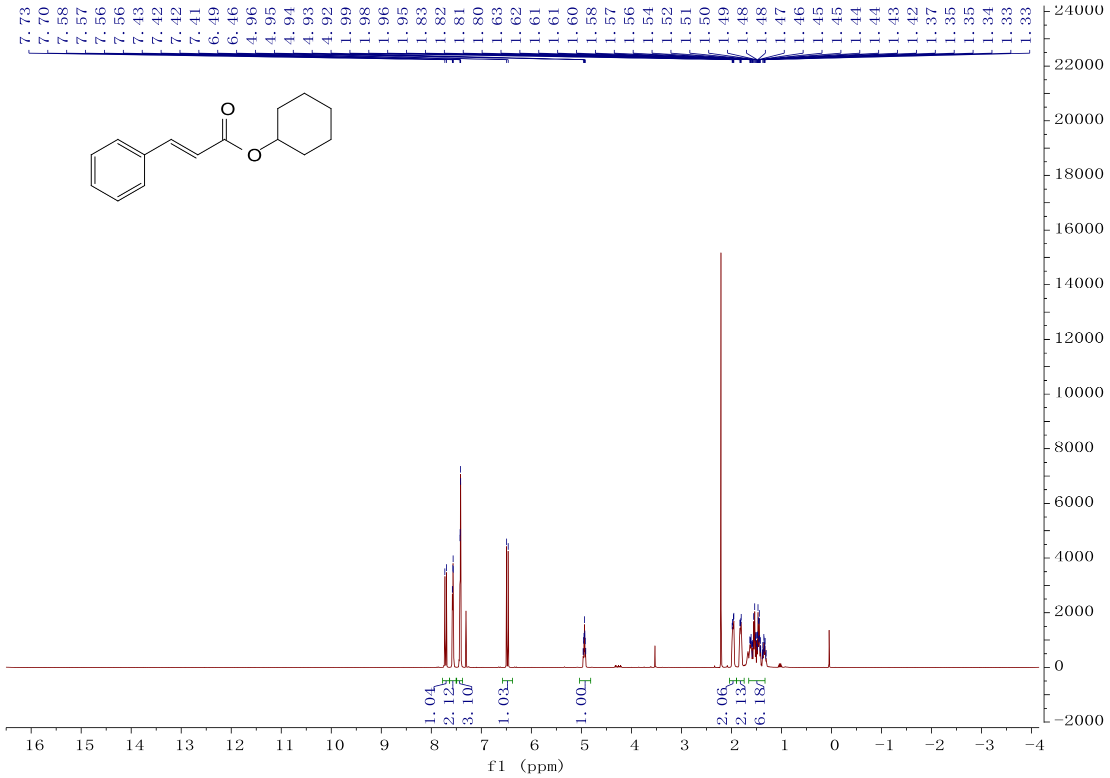

^{1}H NMR of compound B10

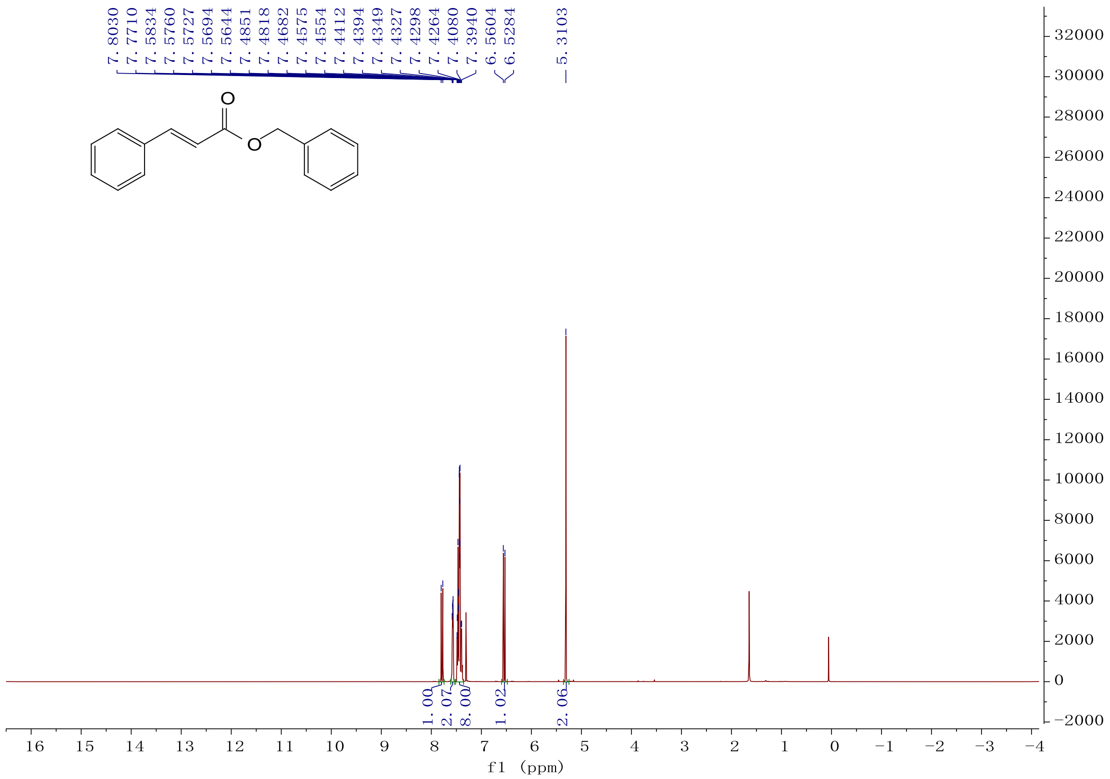

^{1}H NMR of compound B11

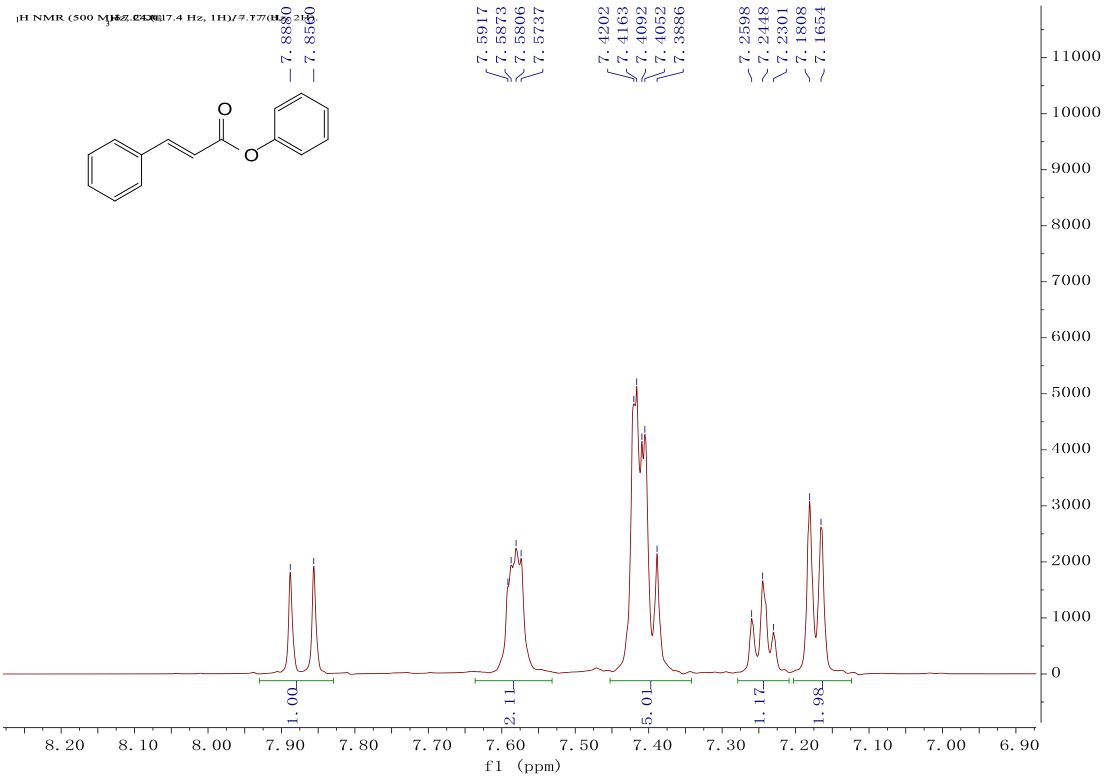

^{1}H NMR of compound B12

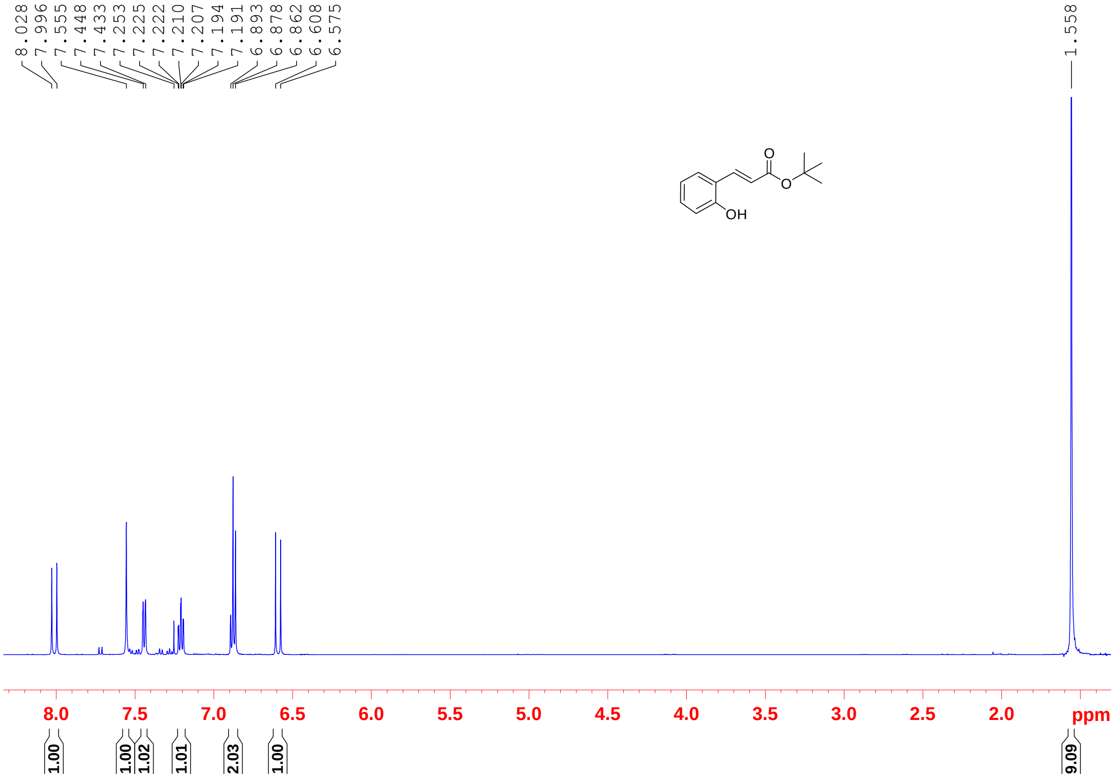

^{1}H NMR of compound C1

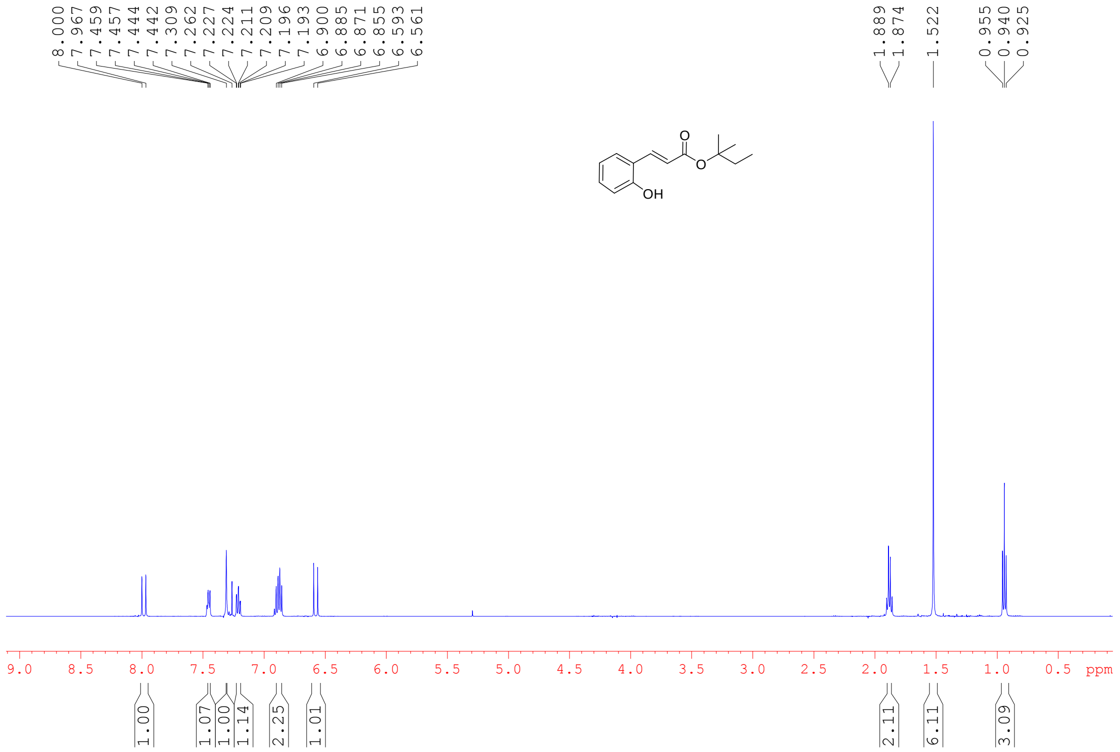

^{1}H NMR of compound C2

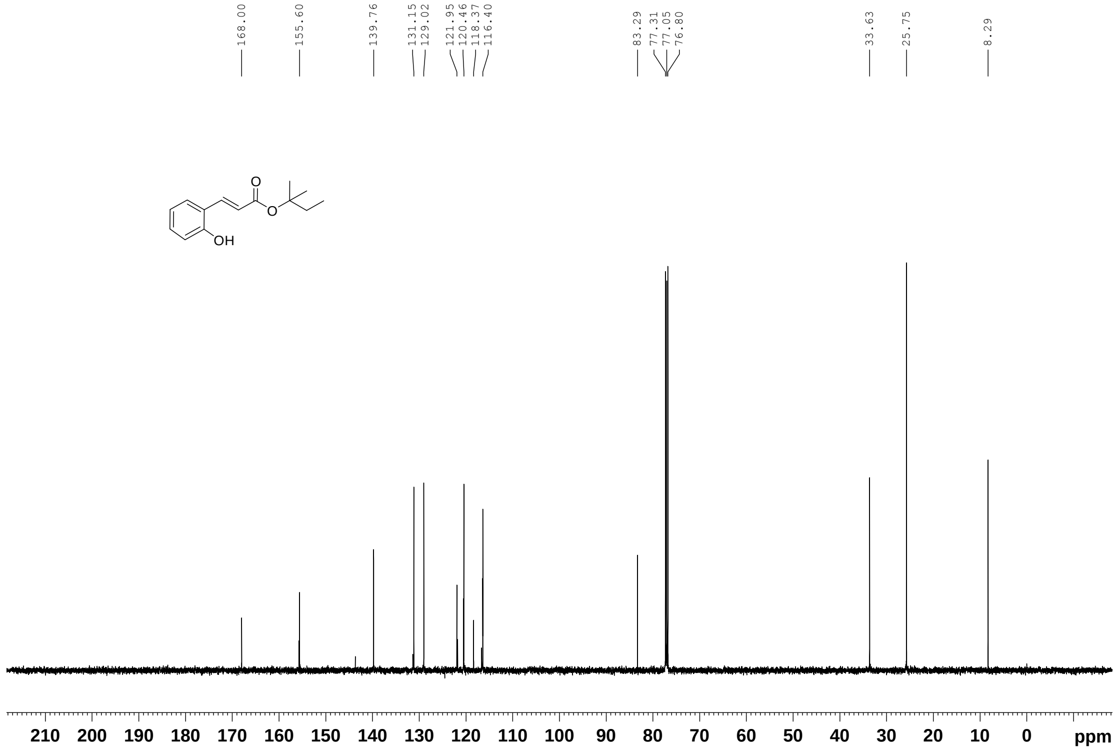

^{13}C NMR of compound C2

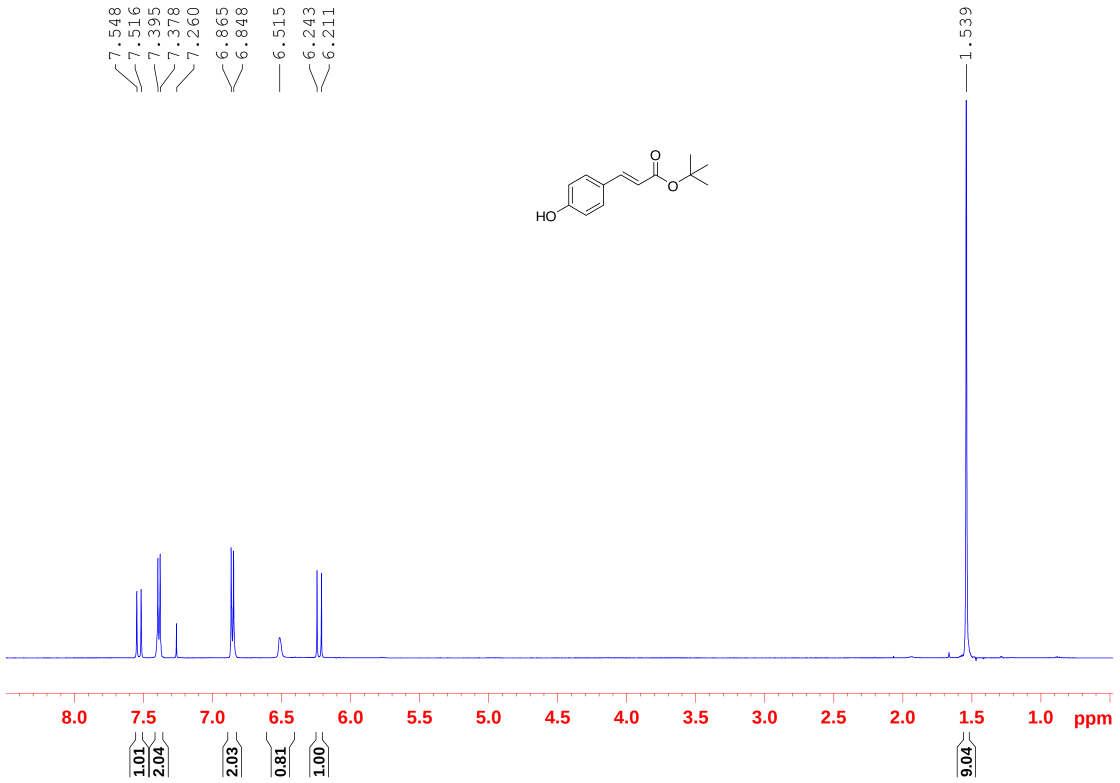

^{1}H NMR of compound C3

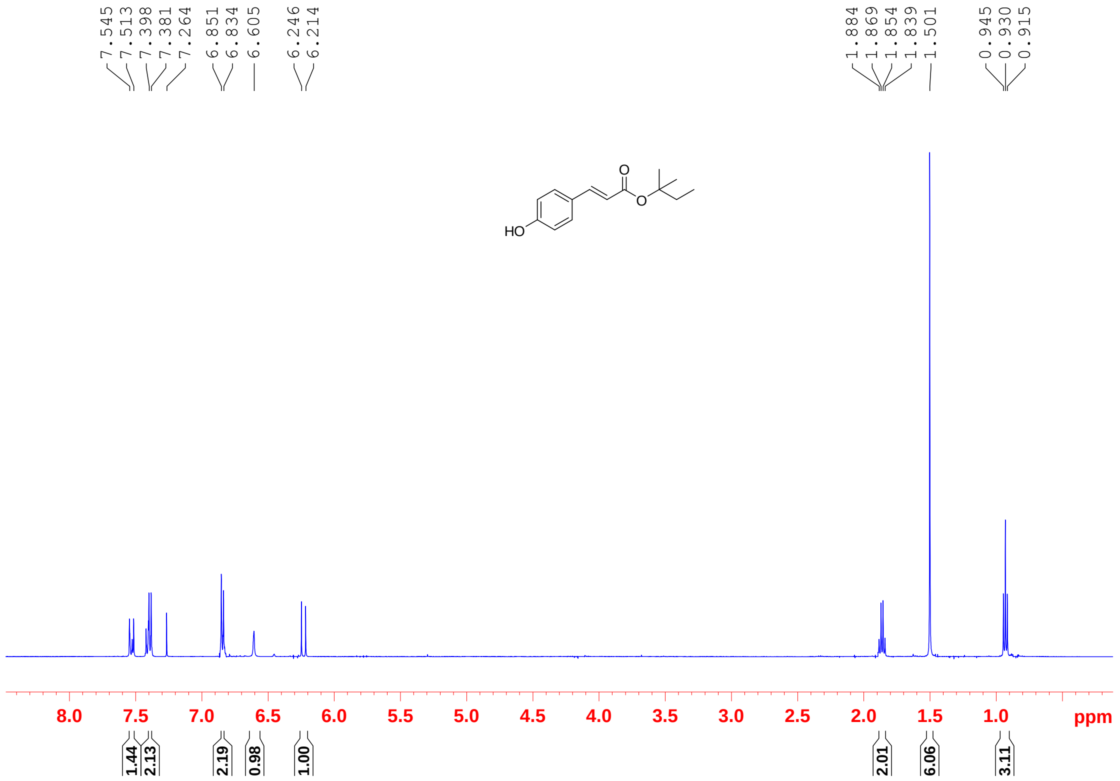

^{1}H NMR of compound C4

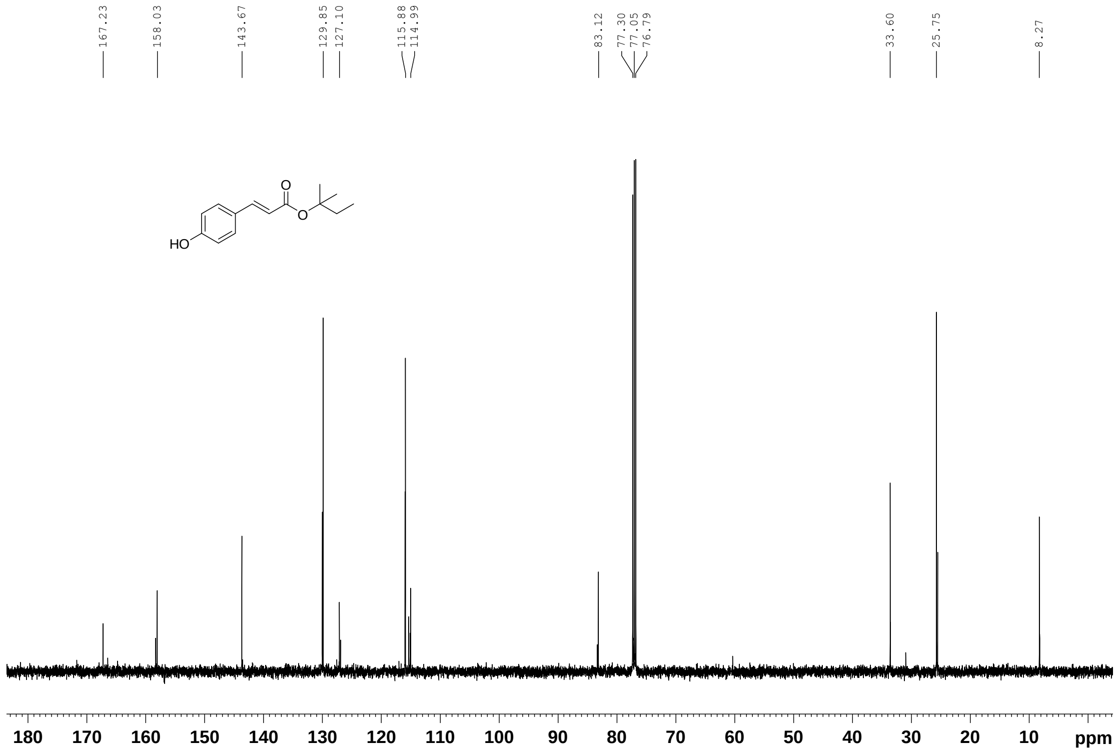

^{13}C NMR of compound C4

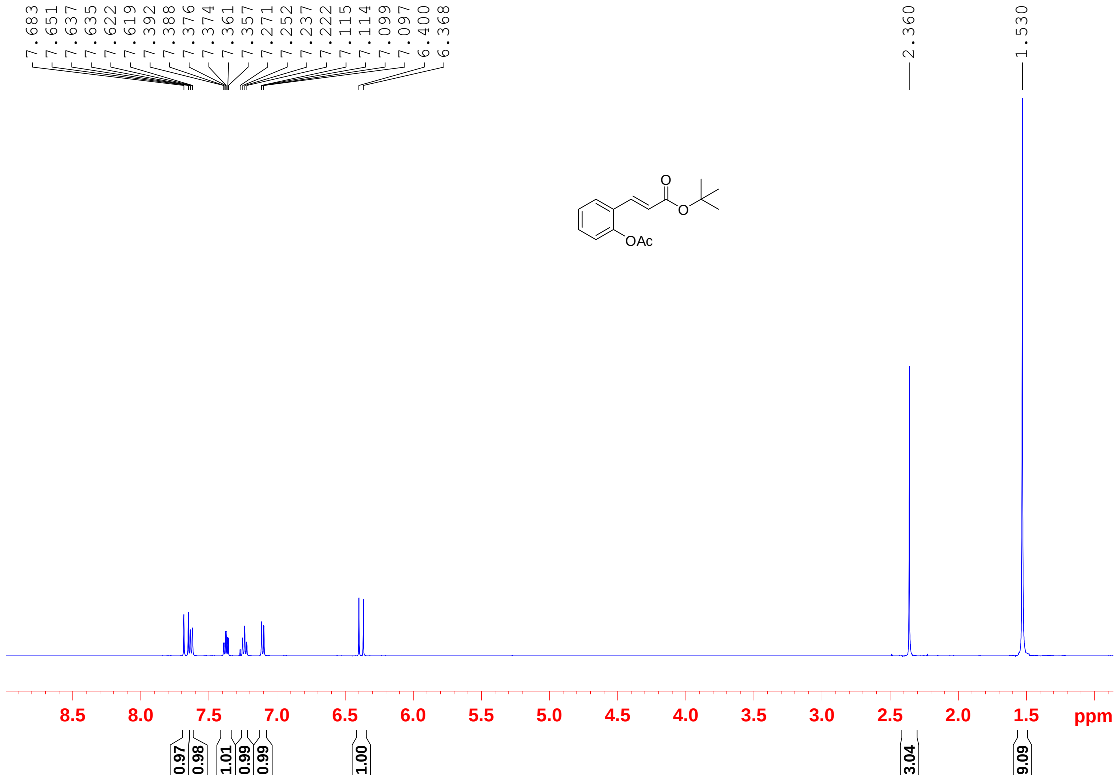

^{1}H NMR of compound C5

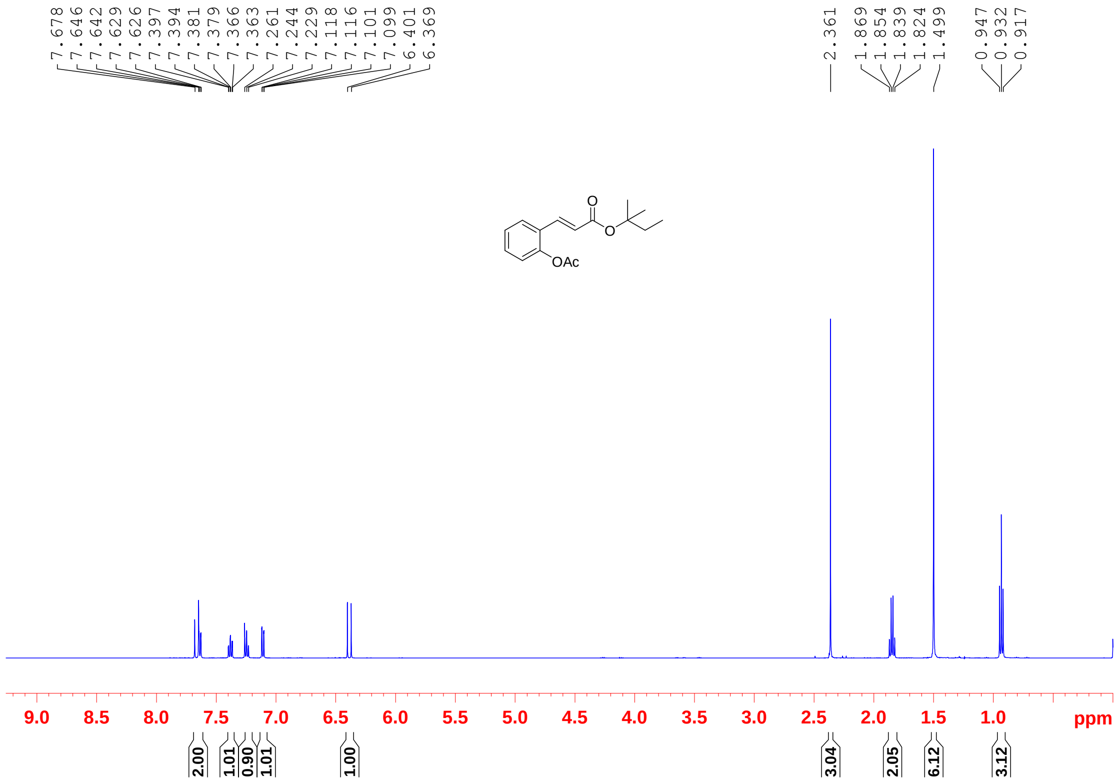

^{1}H NMR of compound C6

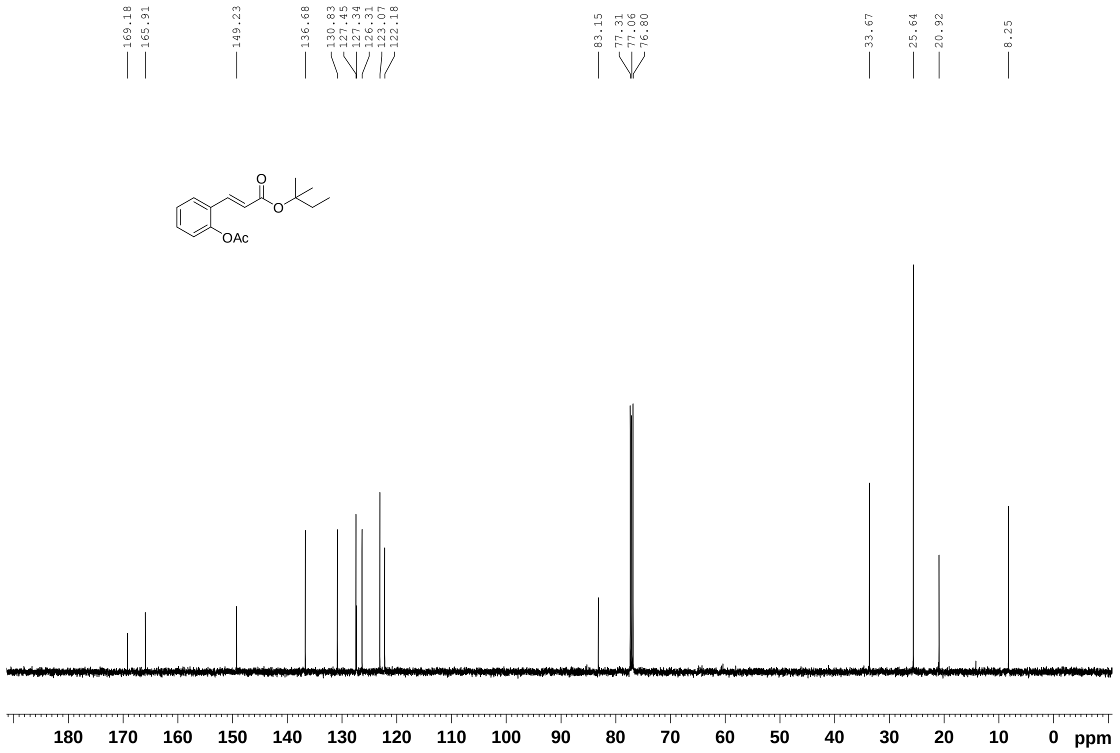

^{13}C NMR of compound C6

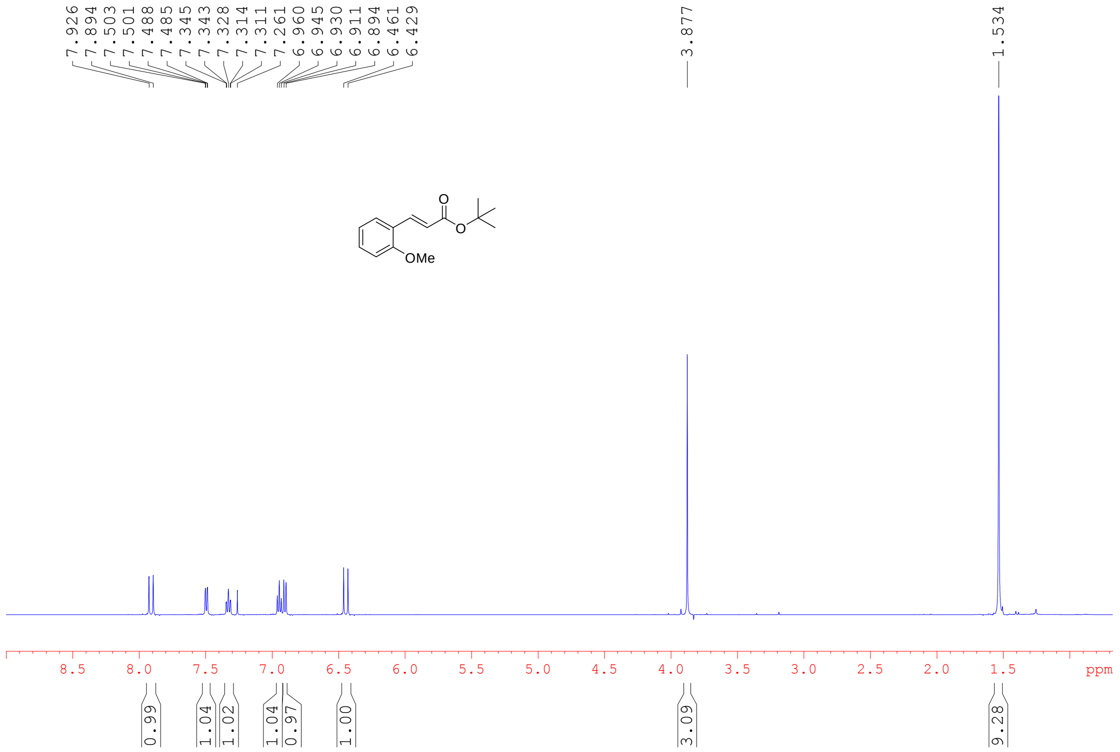

^{1}H NMR of compound C7

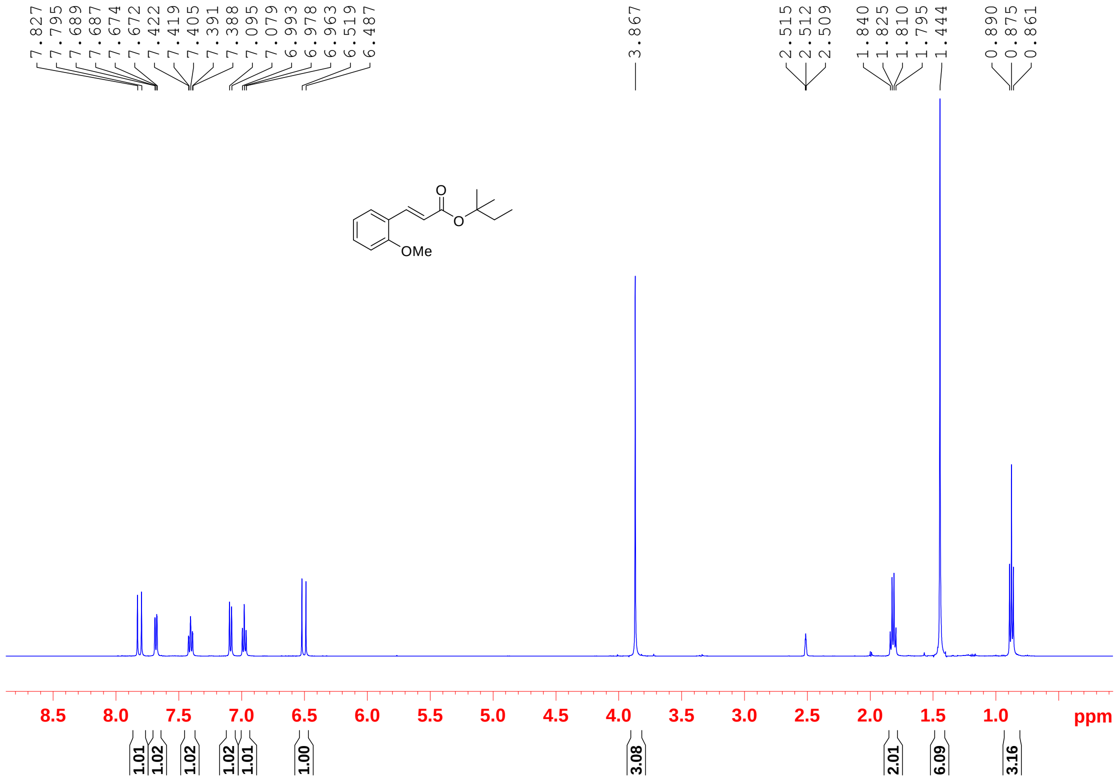

^{1}H NMR of compound C8

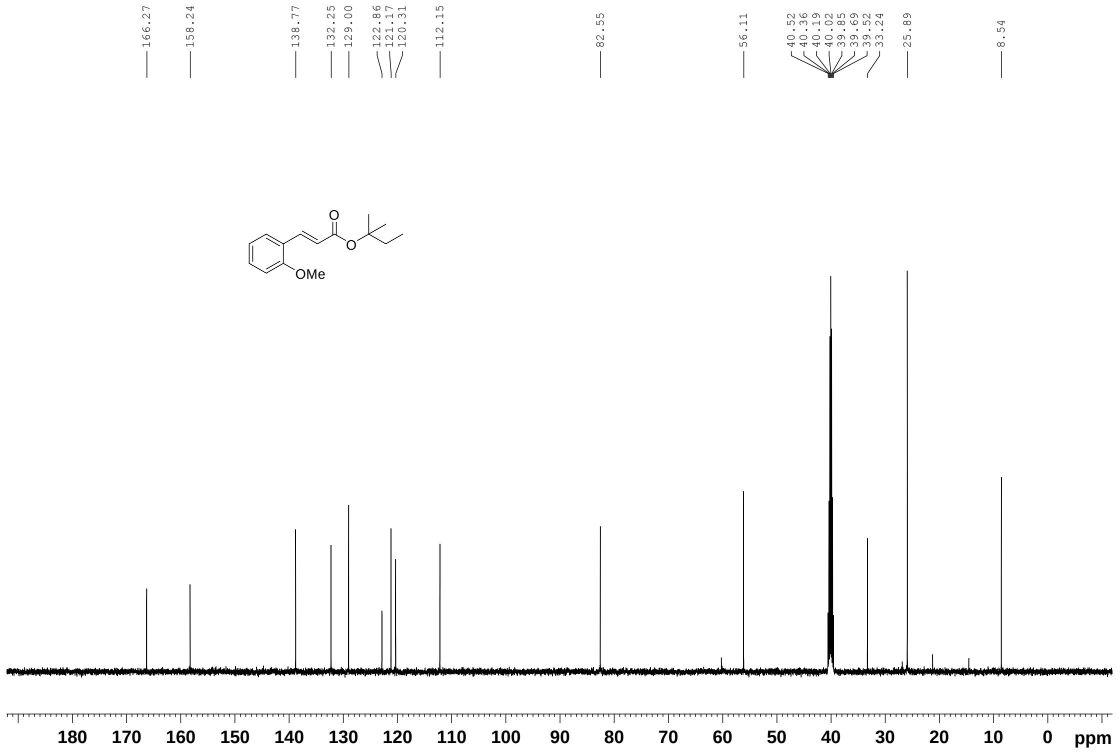

^{13}C NMR of compound C8

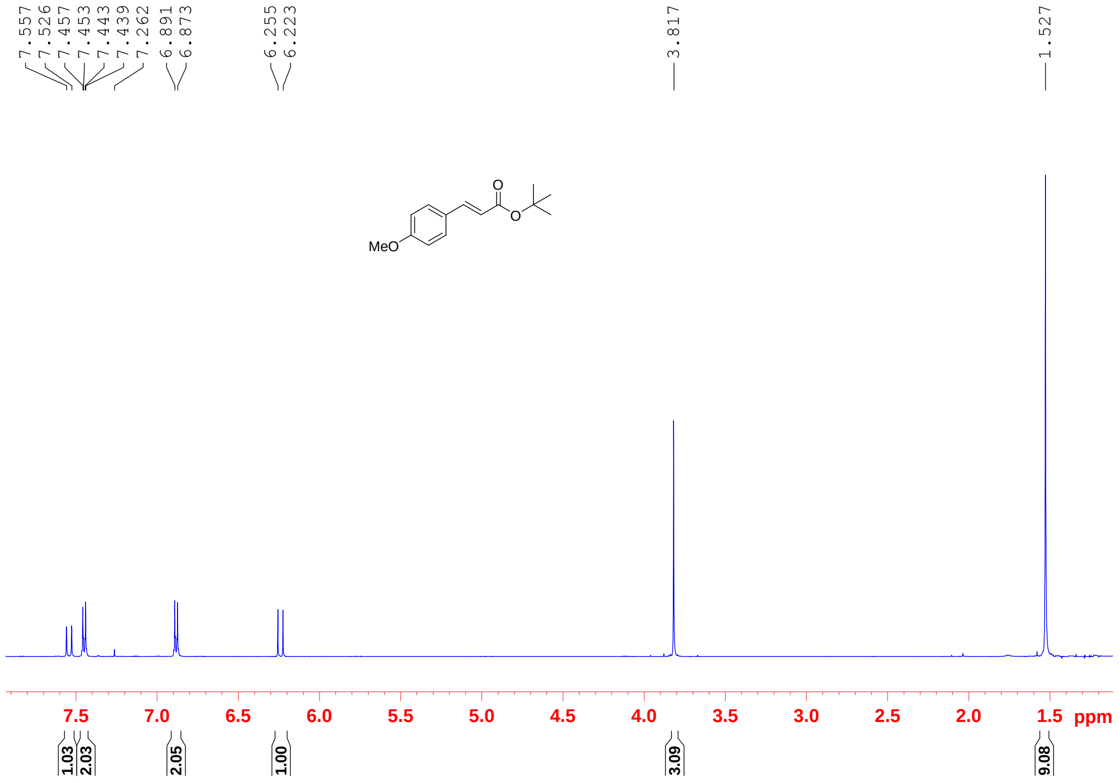

^{1}H NMR of compound C9

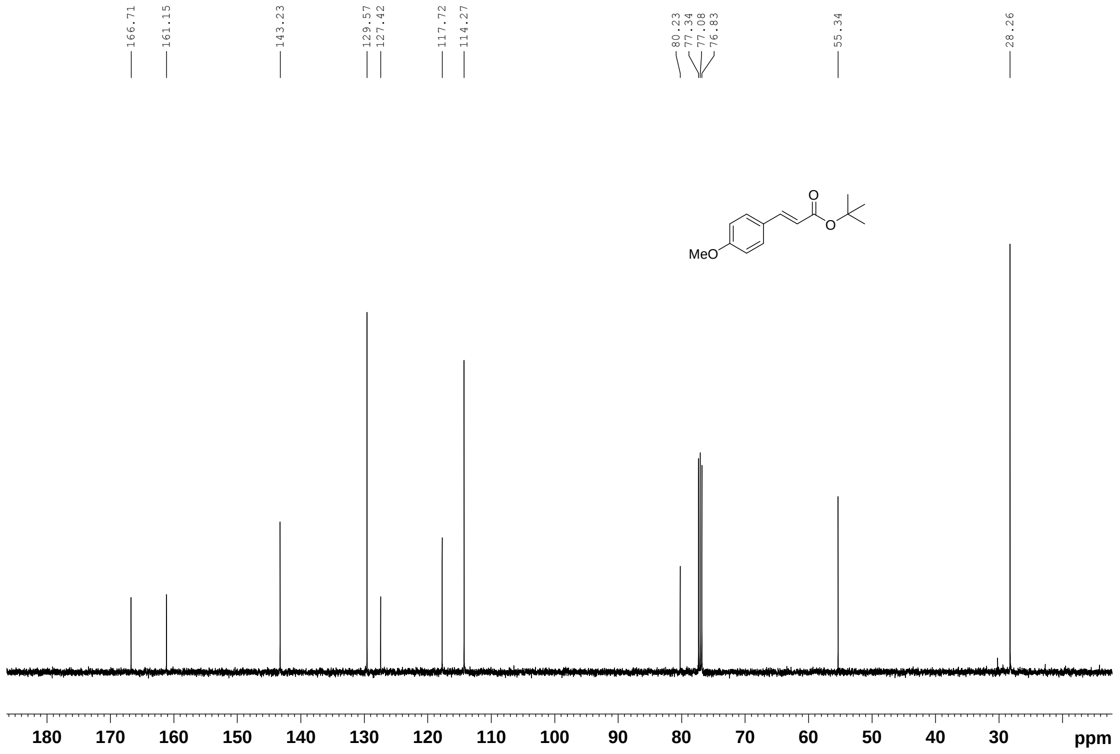

^{13}C NMR of compound C9

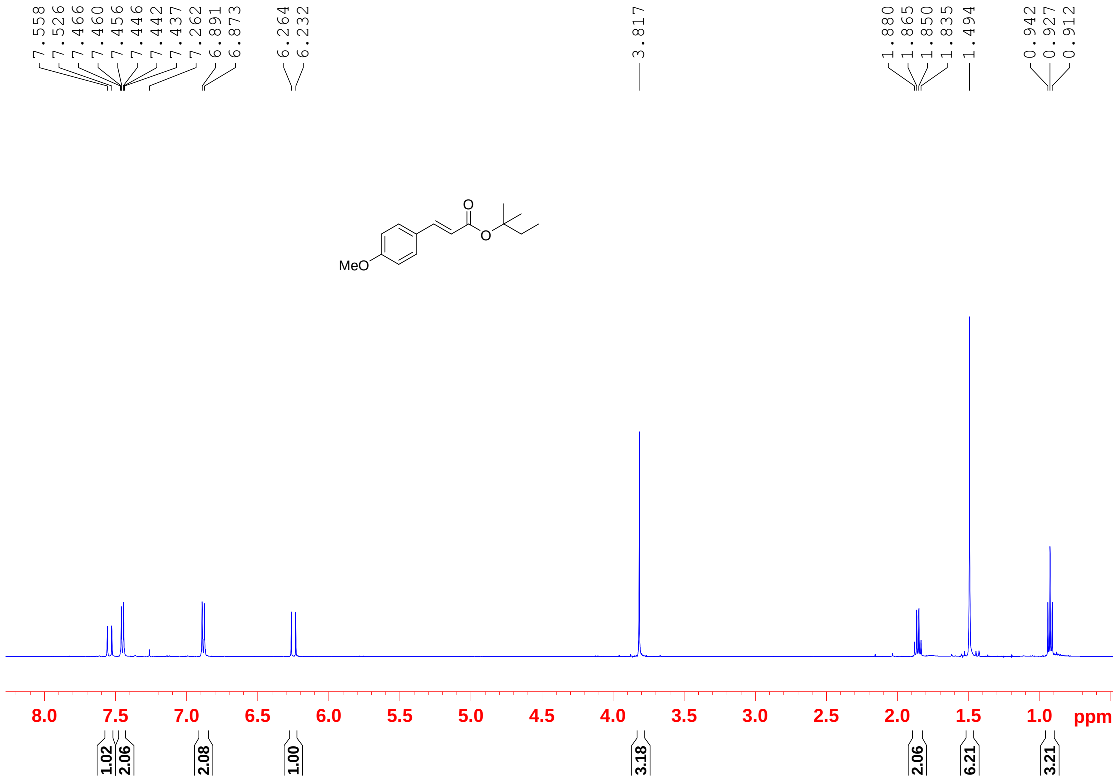

^{1}H NMR of compound C10

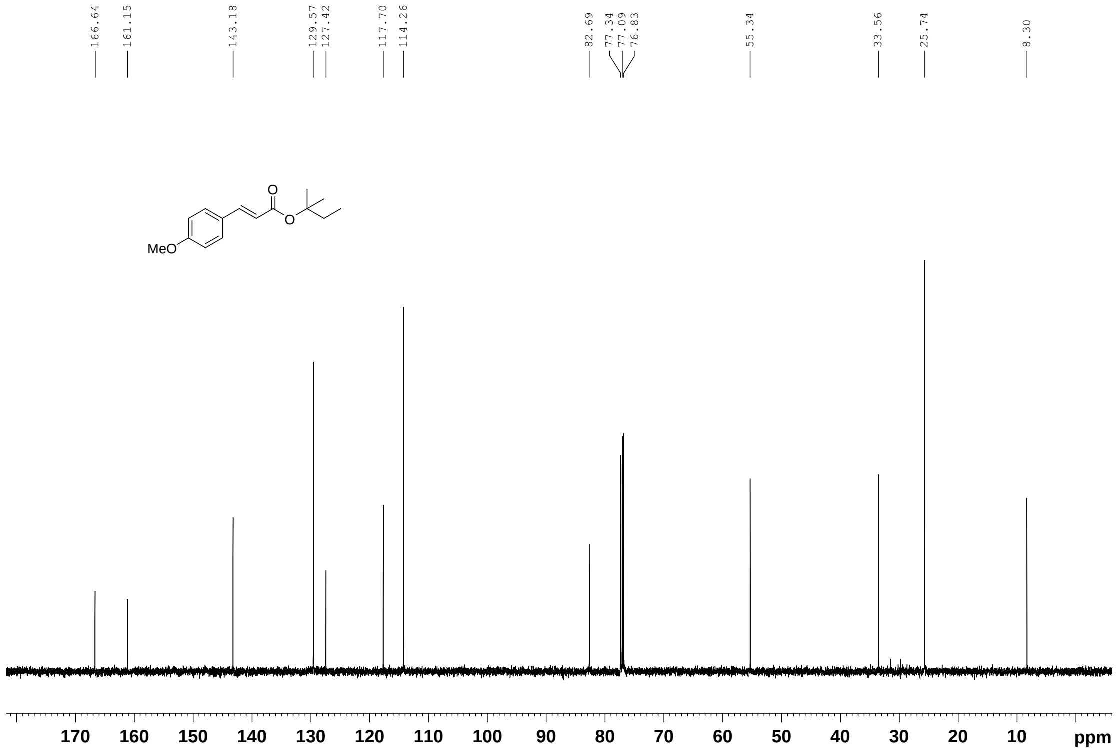

^{13}C NMR of compound C10

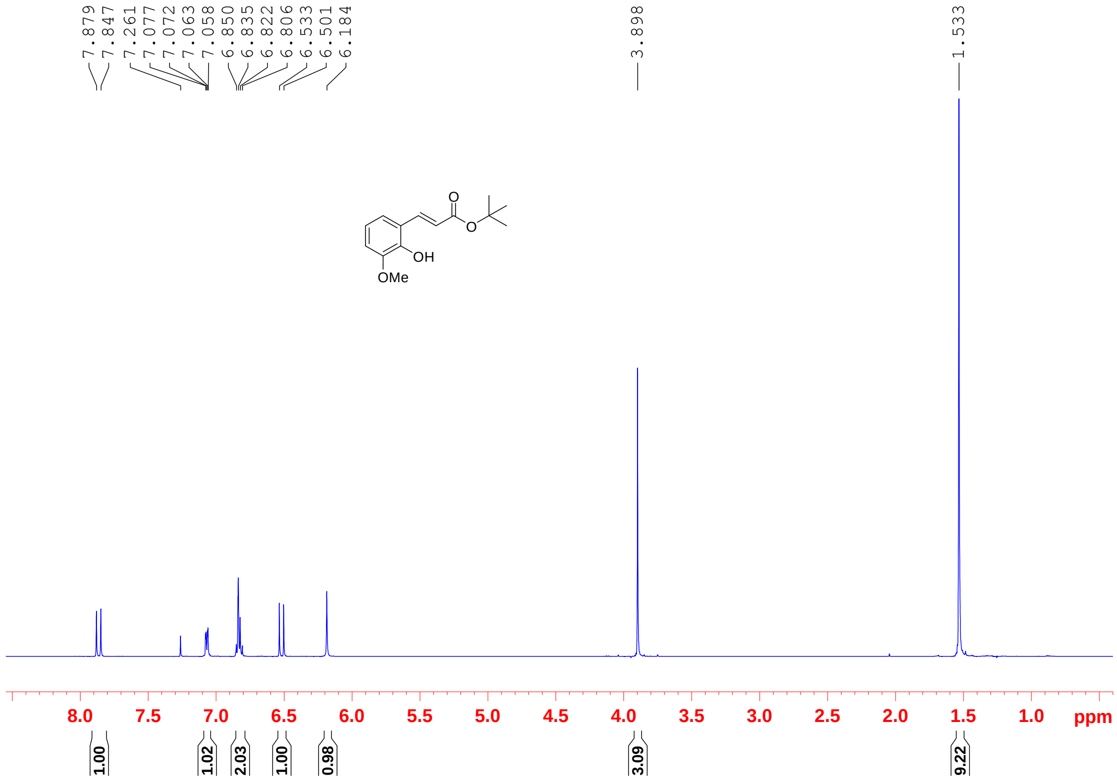

^{1}H NMR of compound C11

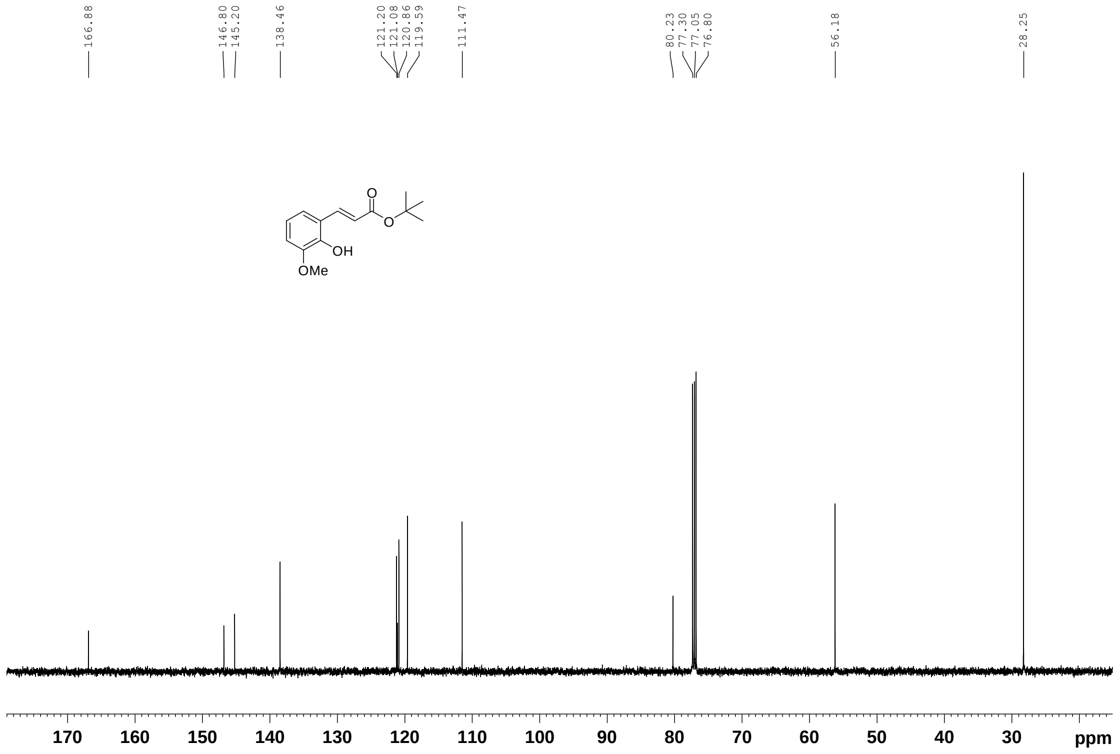

^{13}C NMR of compound C11

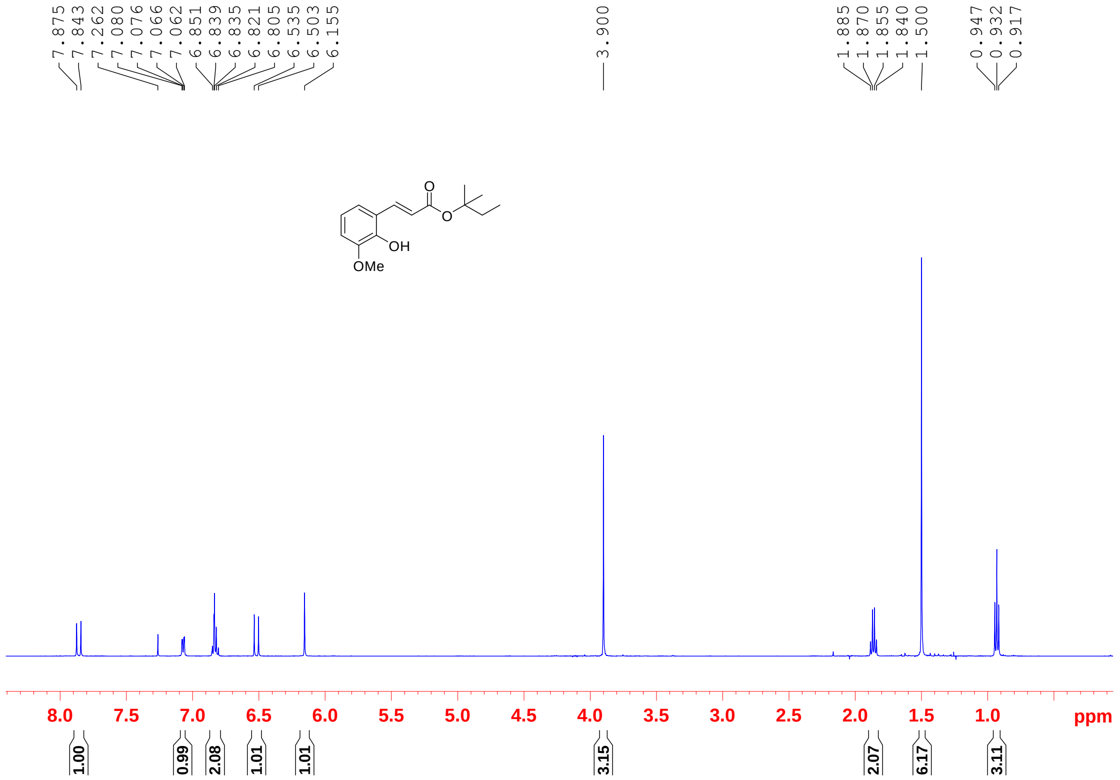

^{1}H NMR of compound C12

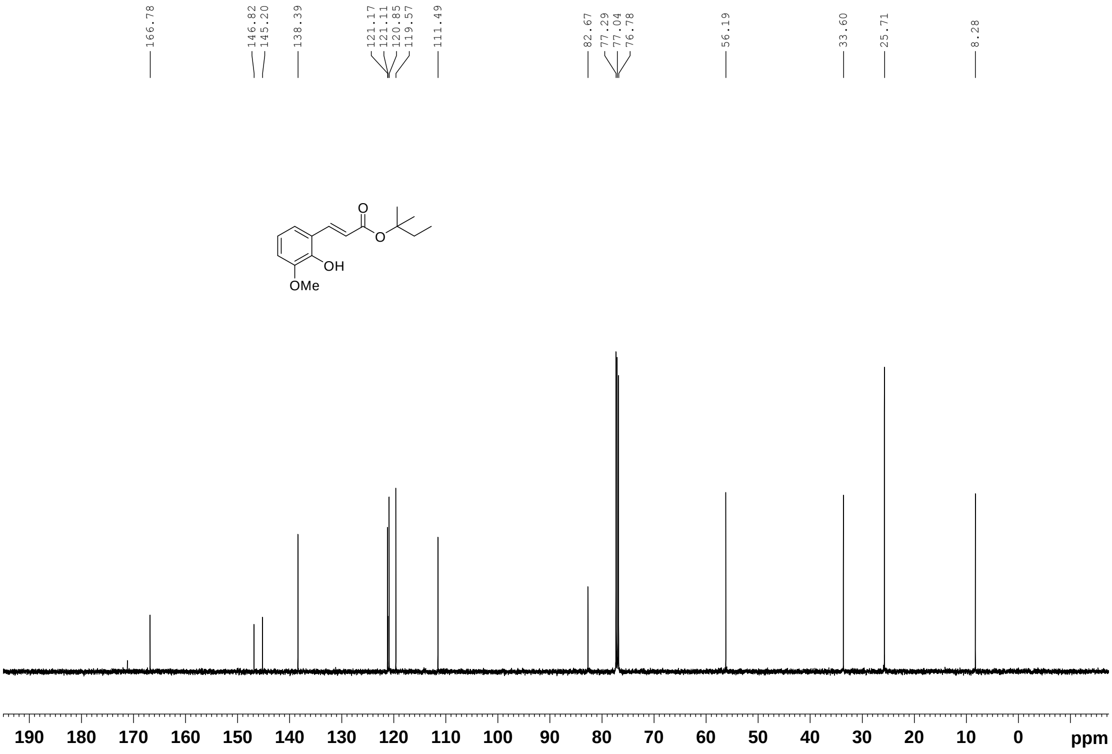

^{13}C NMR of compound C12

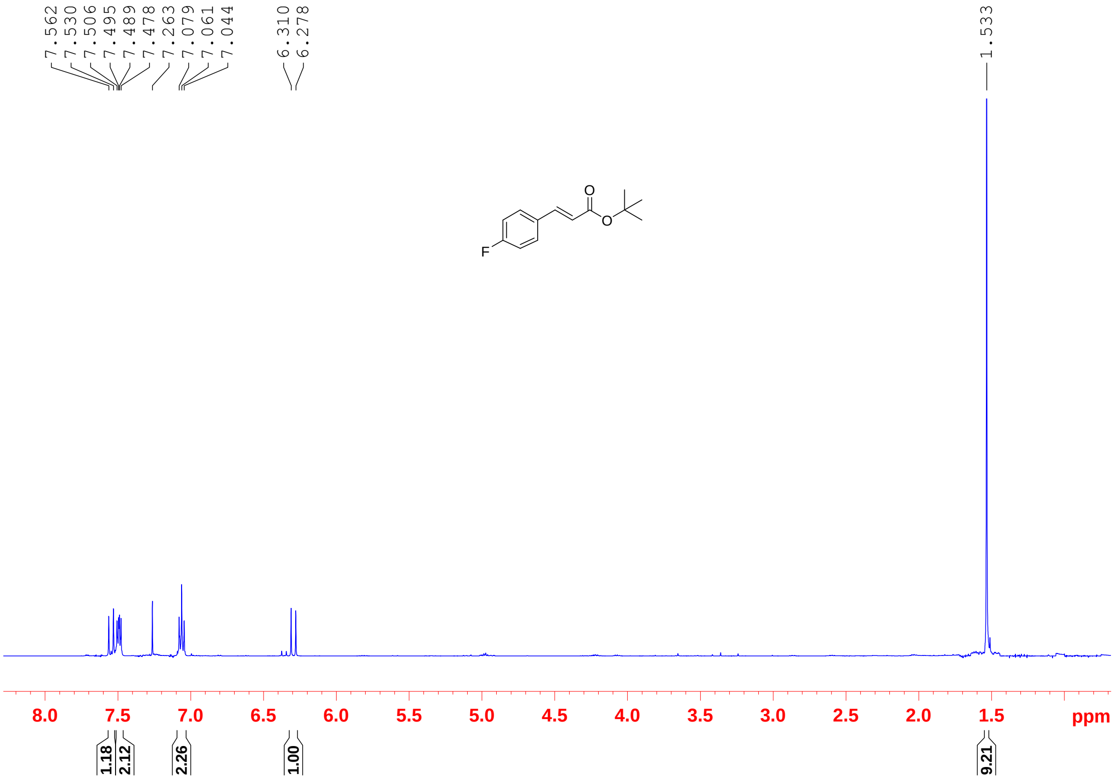

^{1}H NMR of compound C13

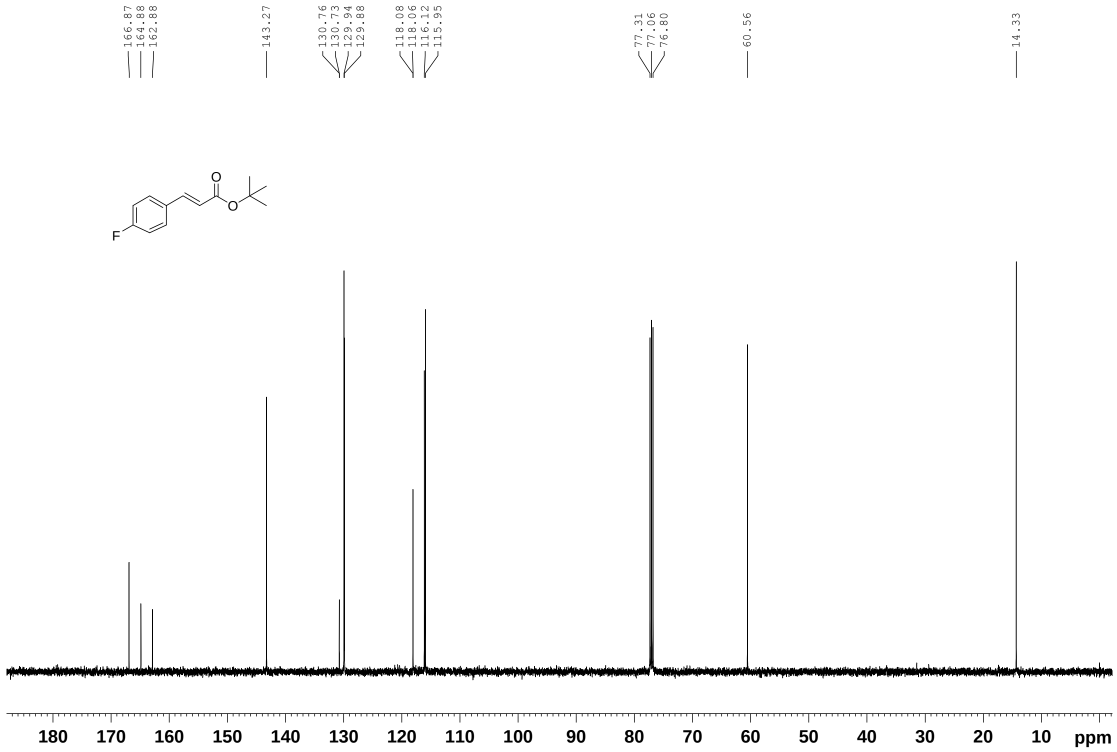

^{13}C NMR of compound C13

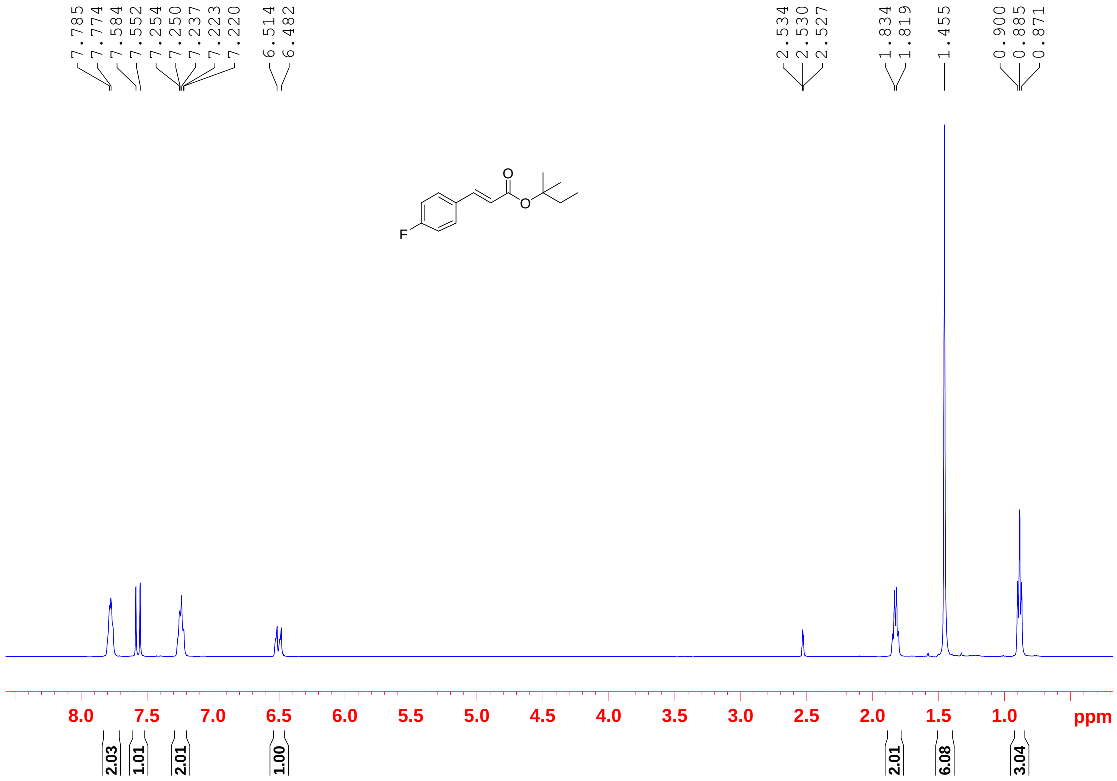

^{1}H NMR of compound C14

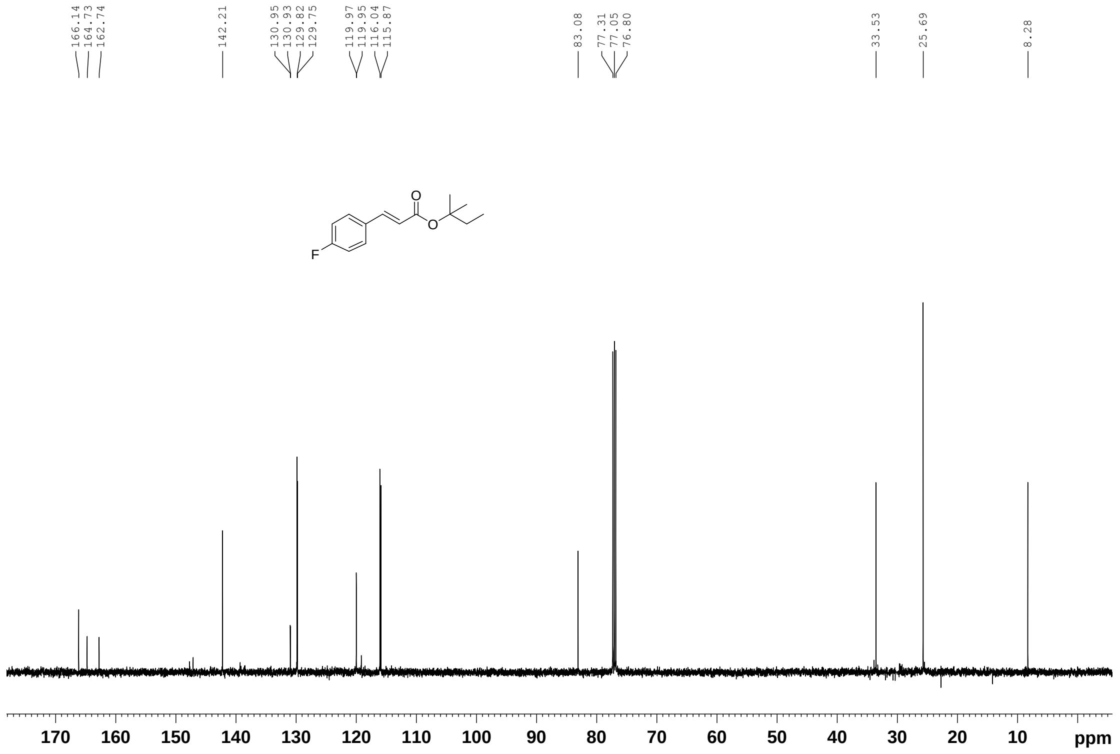

^{13}C NMR of compound C14

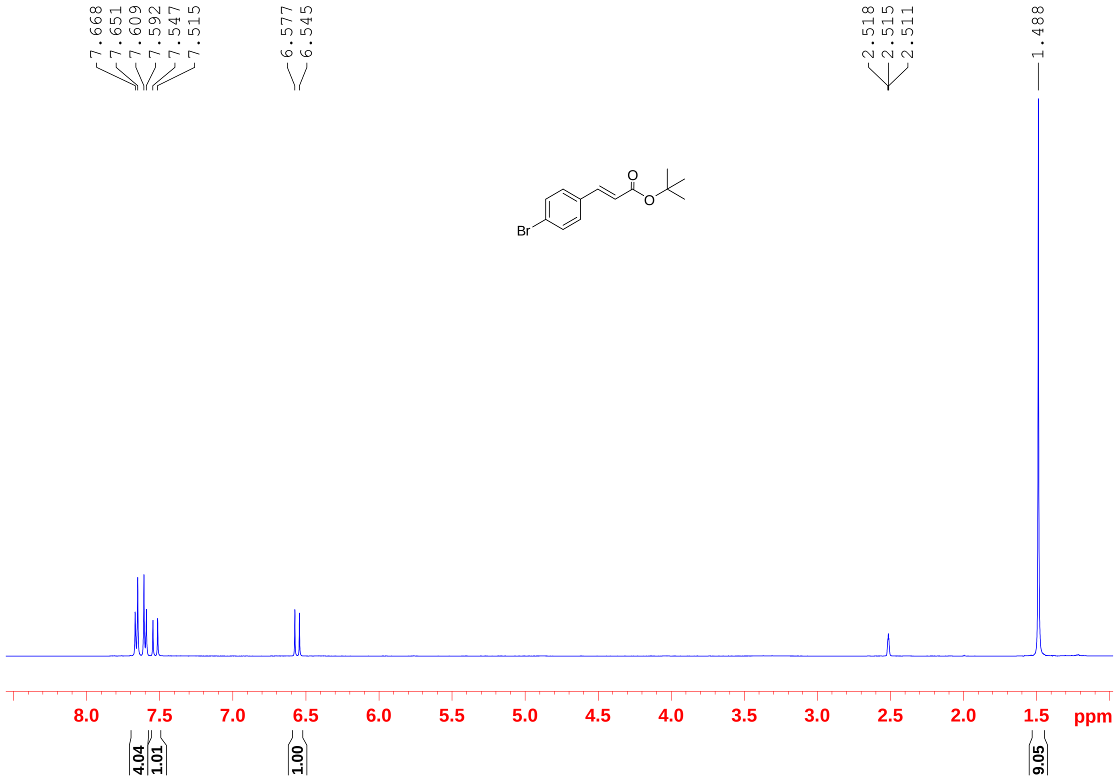

^{1}H NMR of compound C15

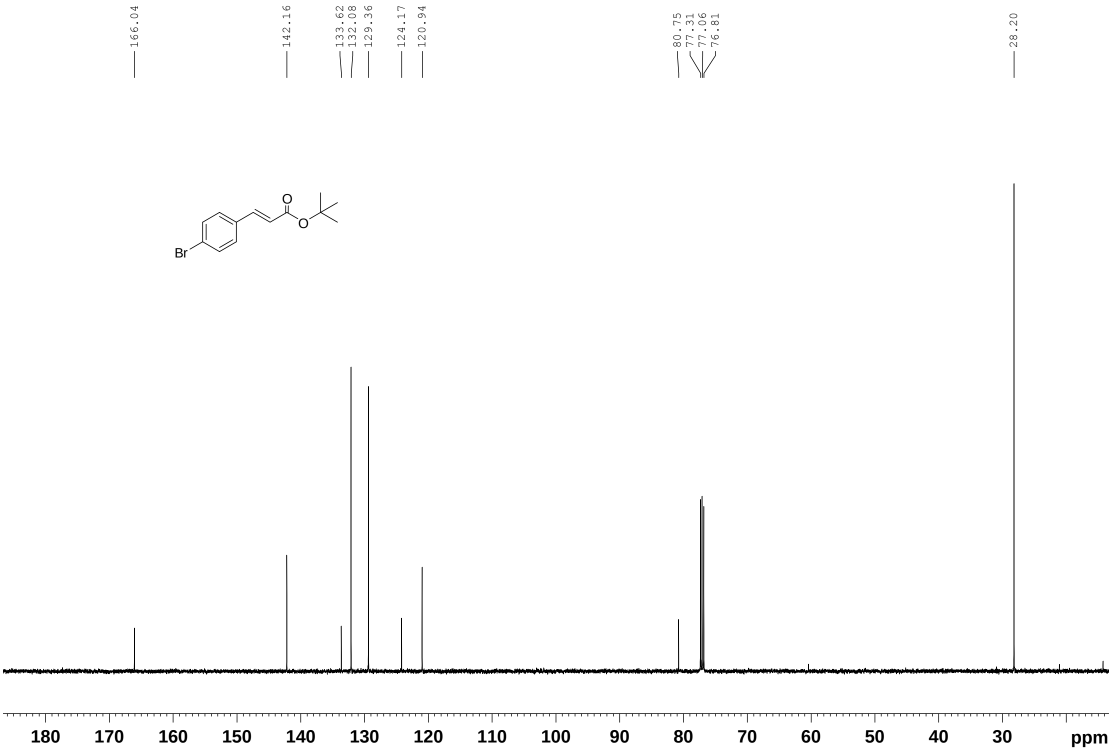

^{13}C NMR of compound C15

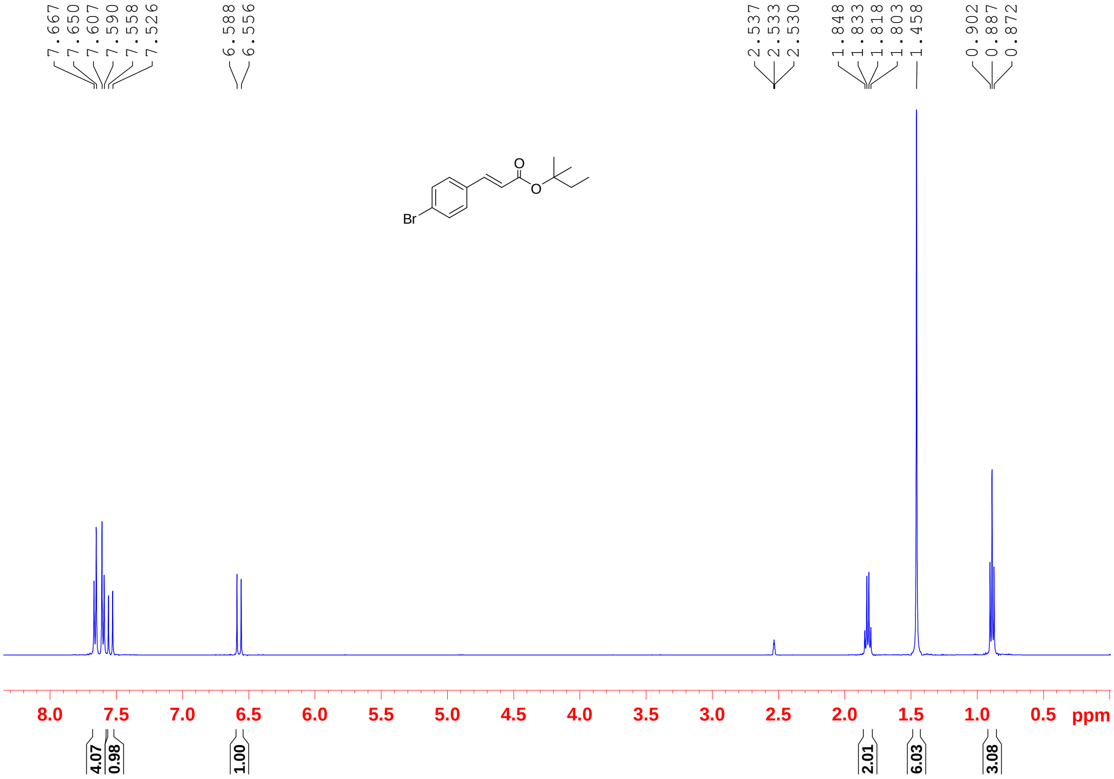

^{1}H NMR of compound C16

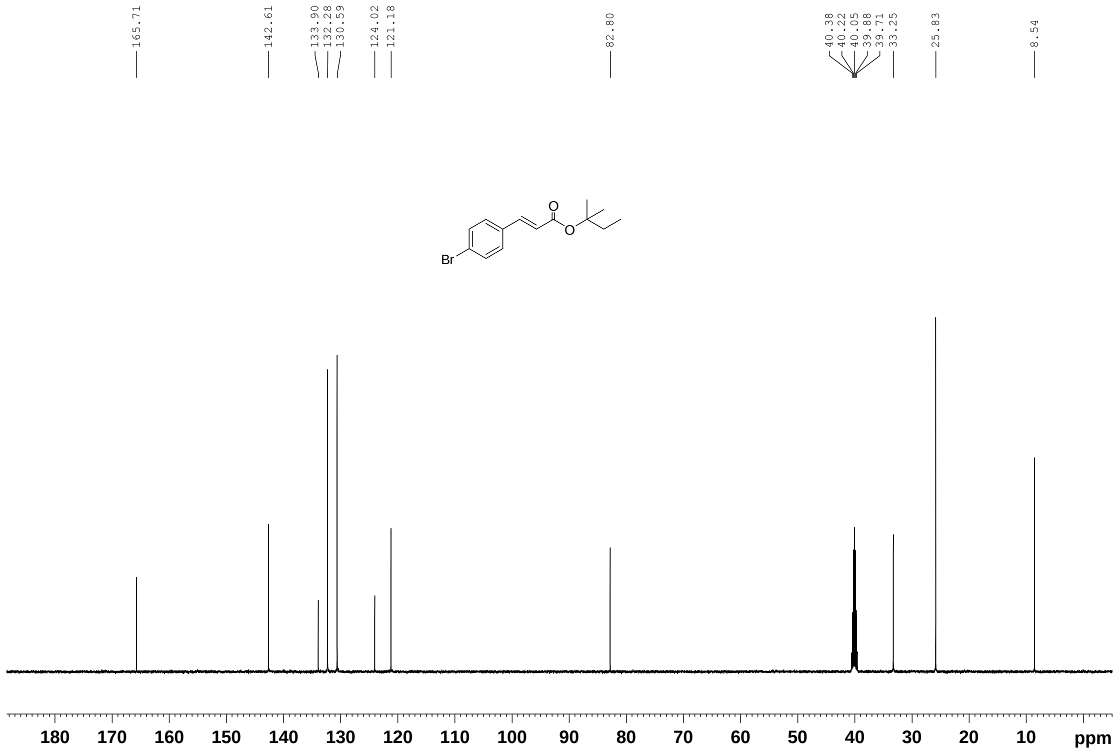

^{13}C NMR of compound C16

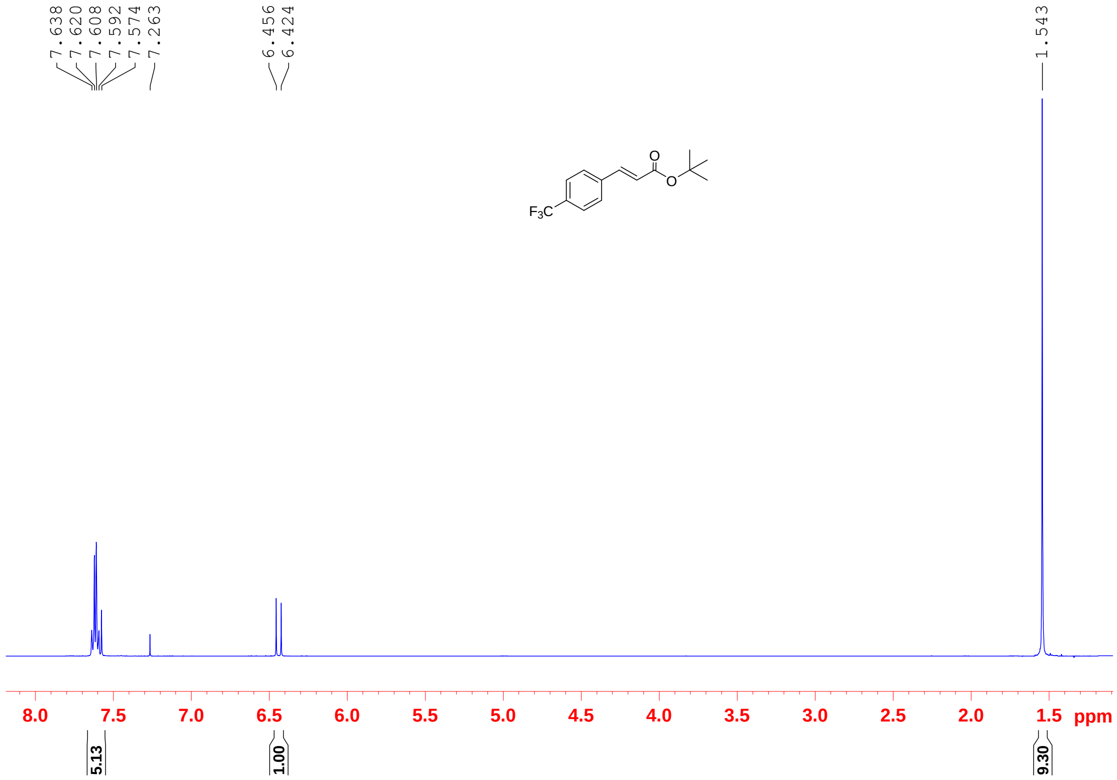

^{1}H NMR of compound C17

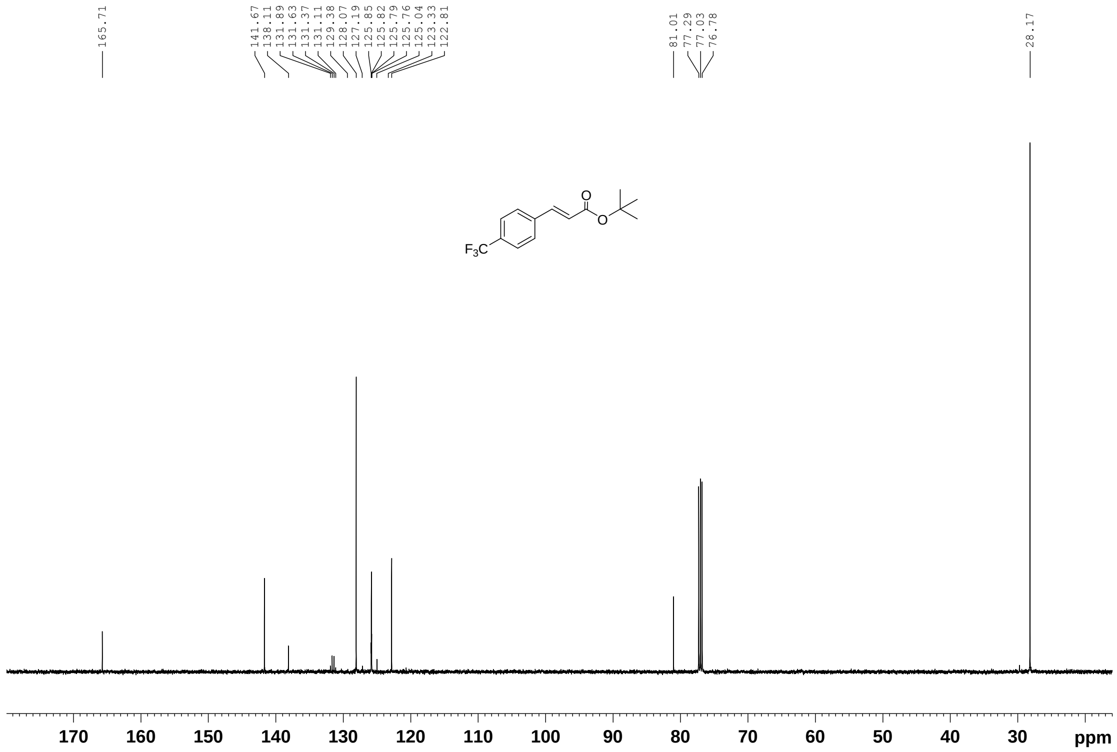

^{13}C NMR of compound C17

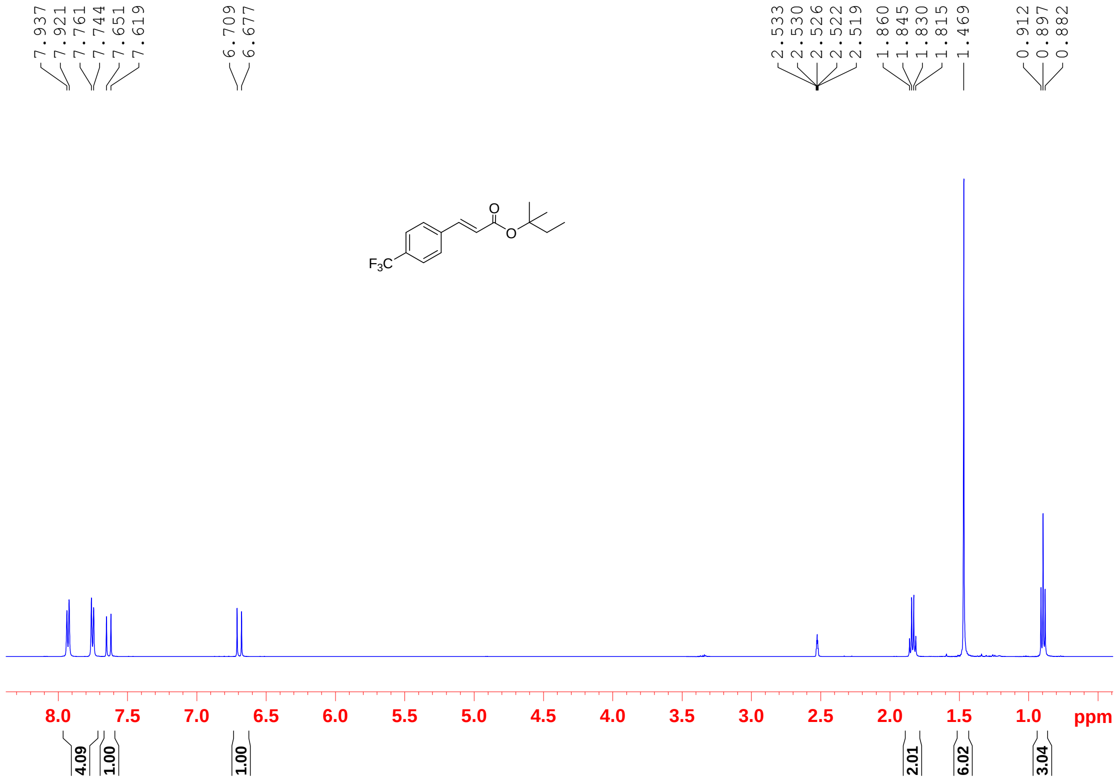

^{1}H NMR of compound C18

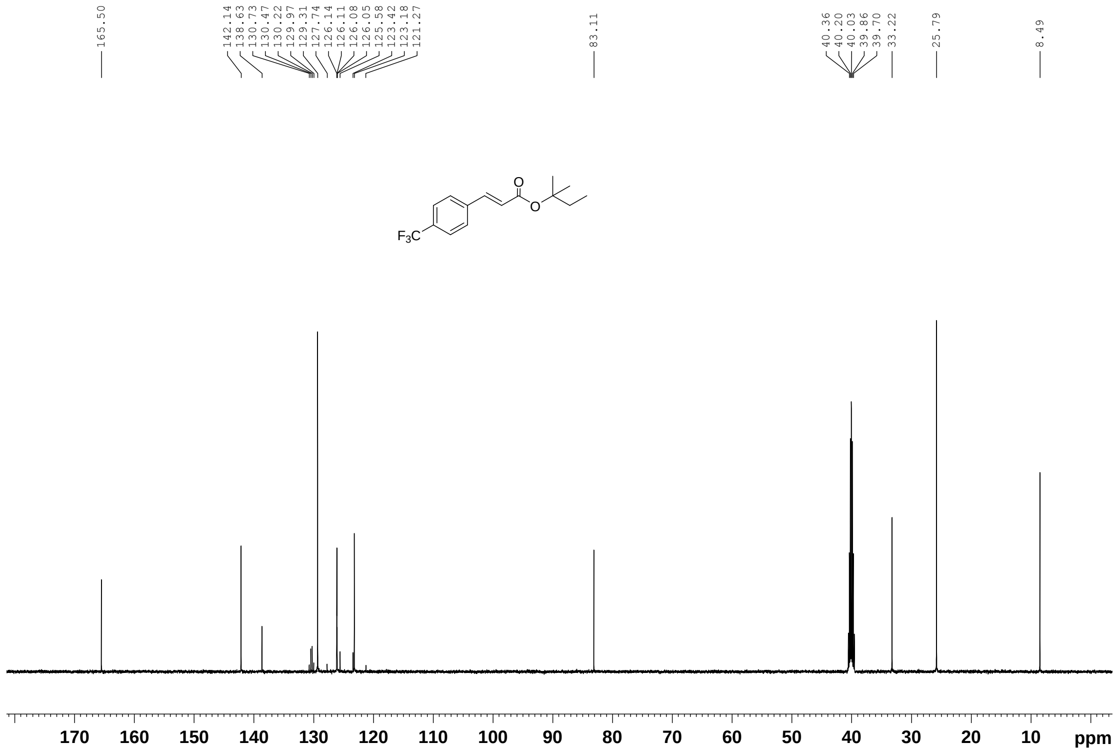

^{13}C NMR of compound C18

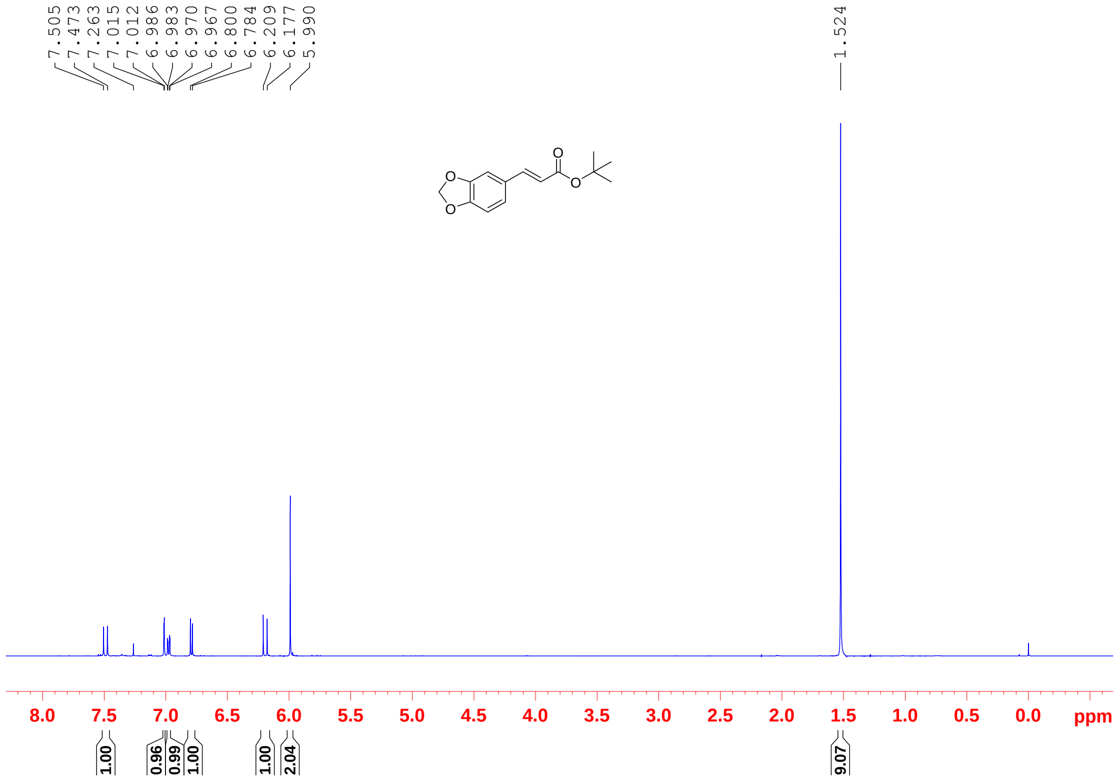

^{1}H NMR of compound C19

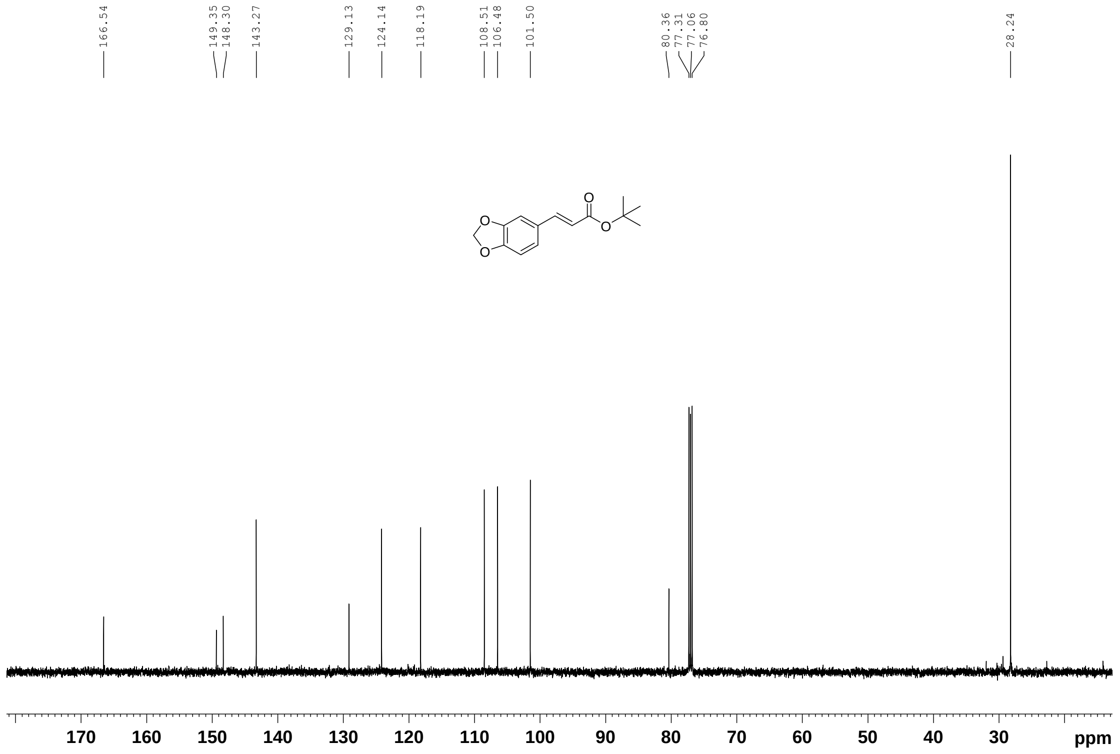

^{13}C NMR of compound C19

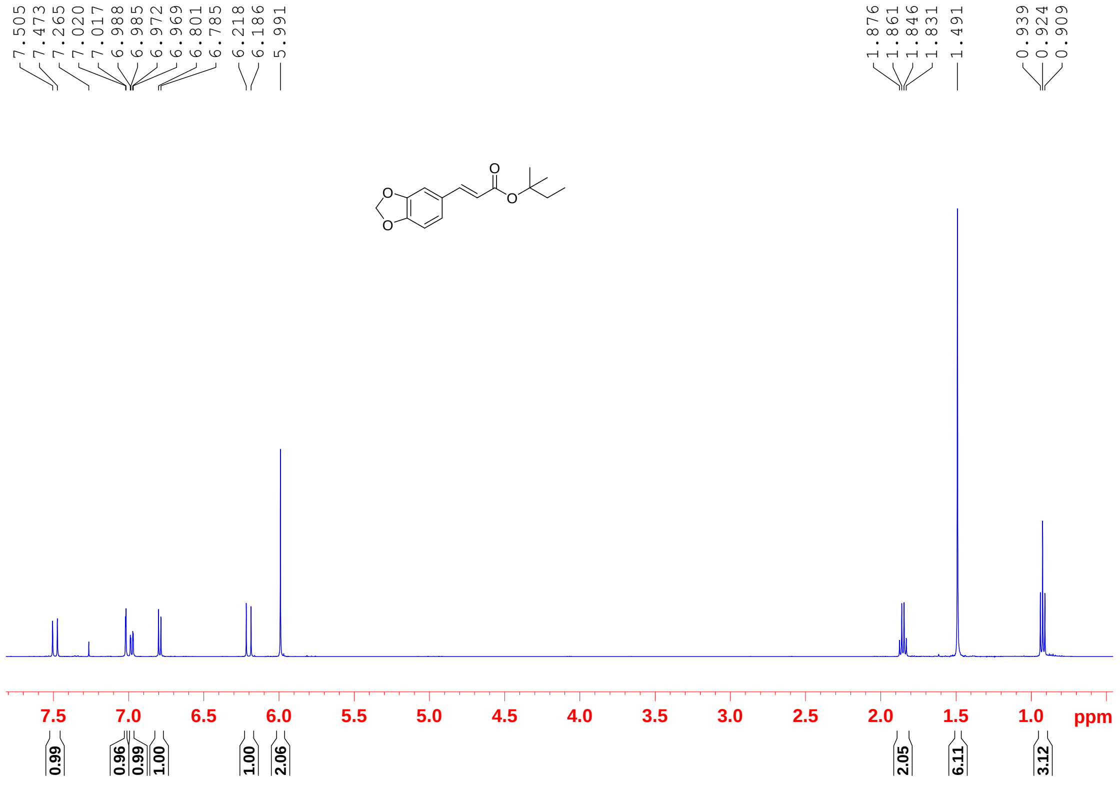

^{1}H NMR of compound C20

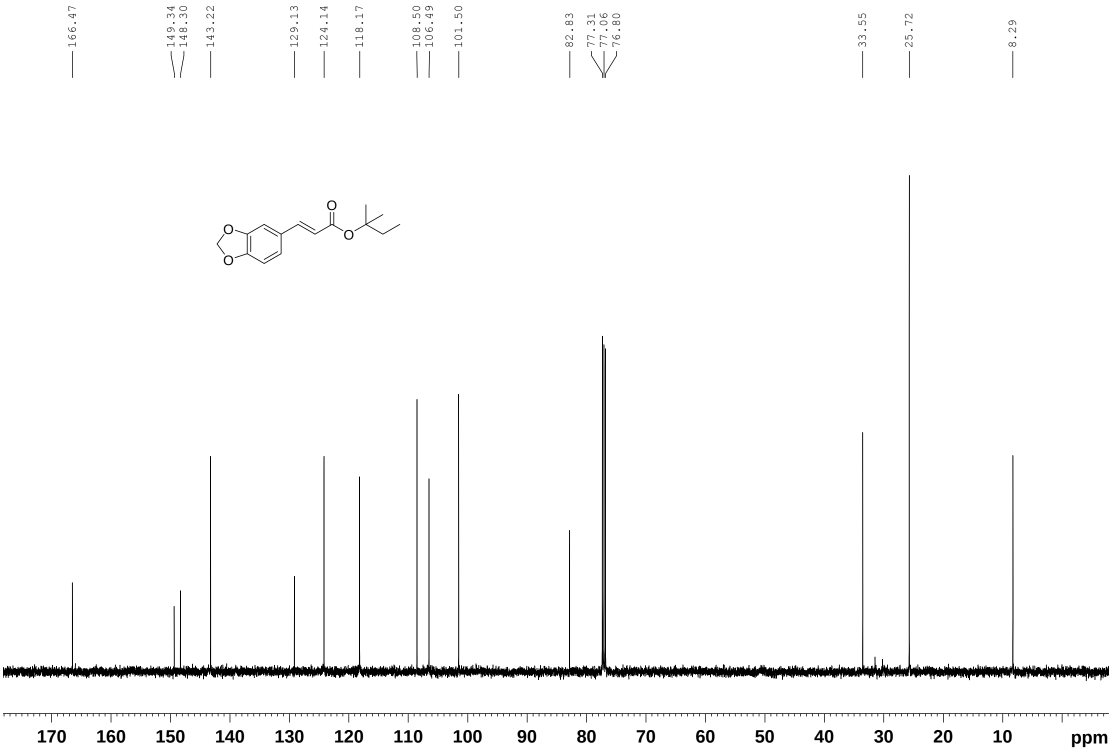

^{13}C NMR of compound C20

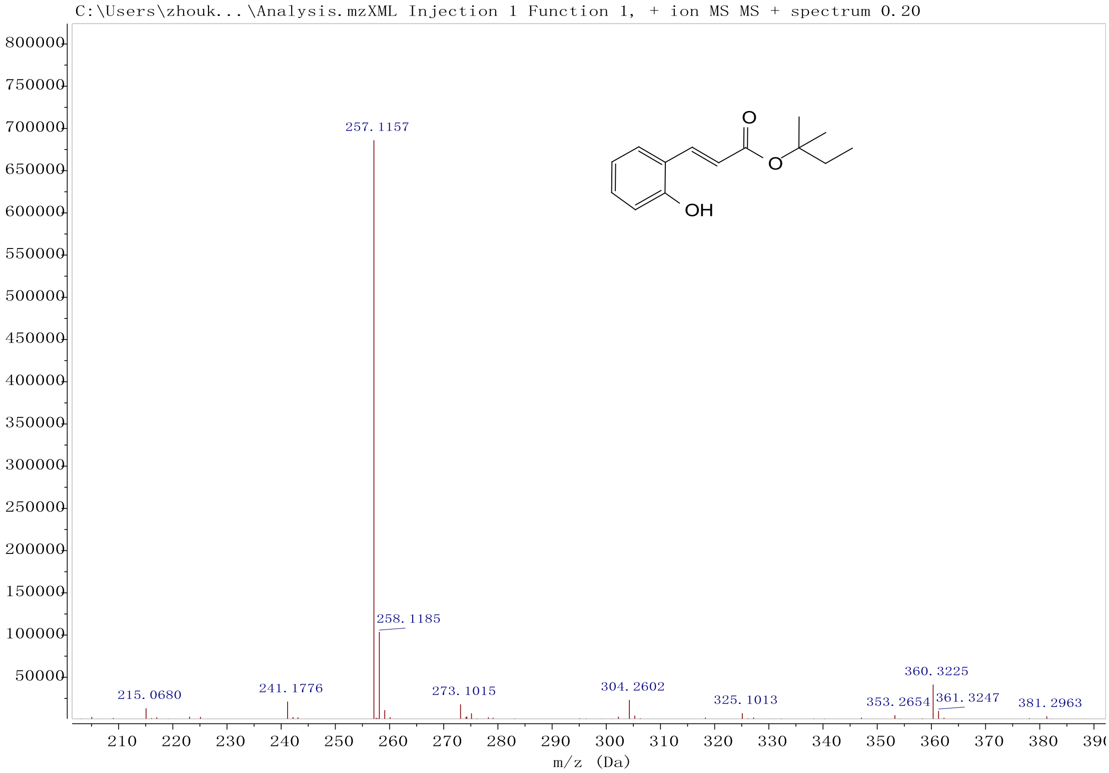

HR-ESI-MS of compound C2

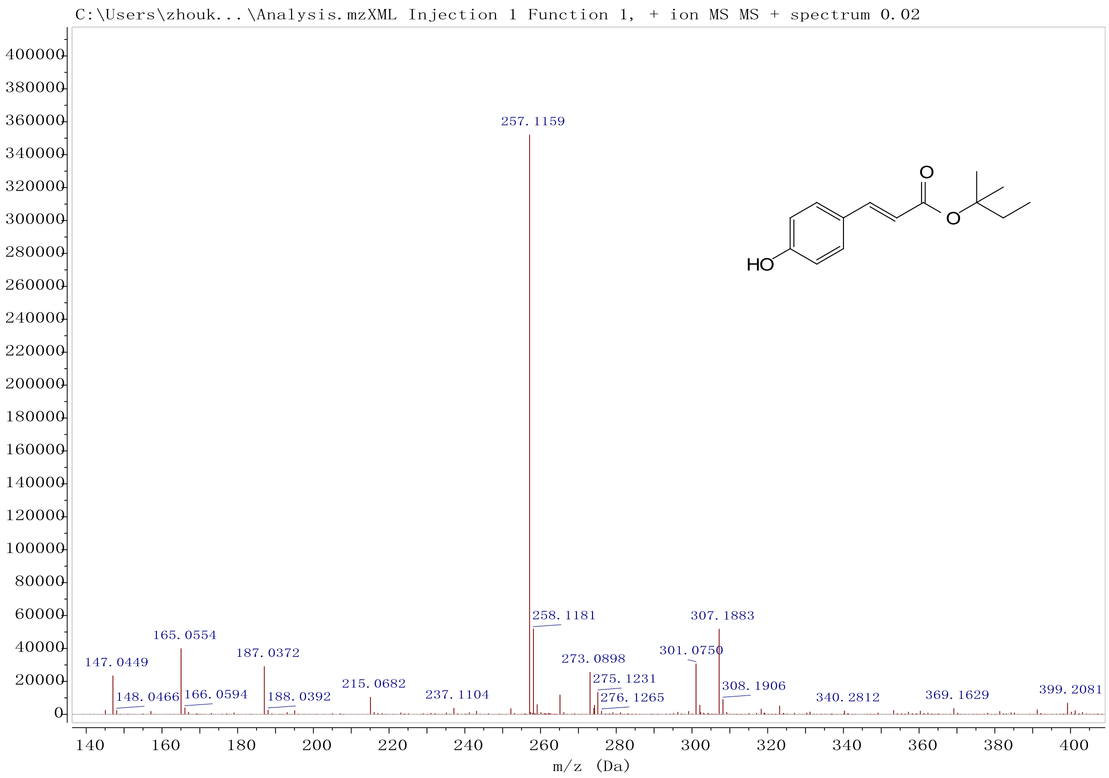

HR-ESI-MS of compound C4

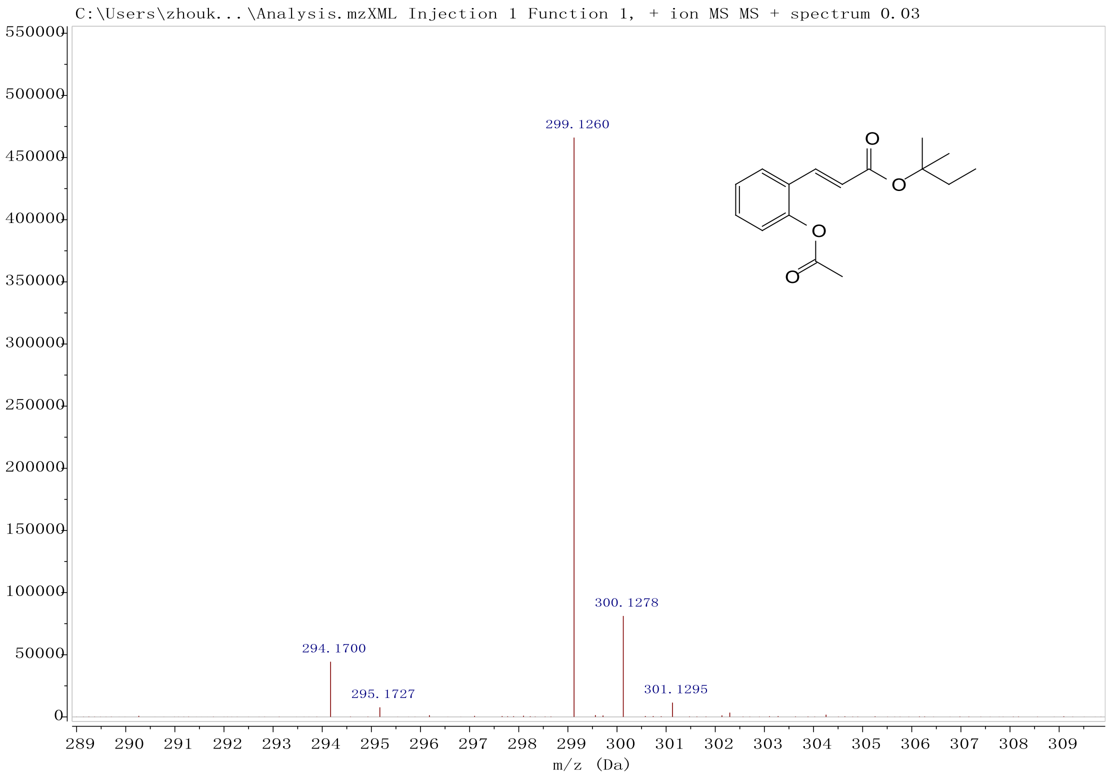

HR-ESI-MS of compound C6

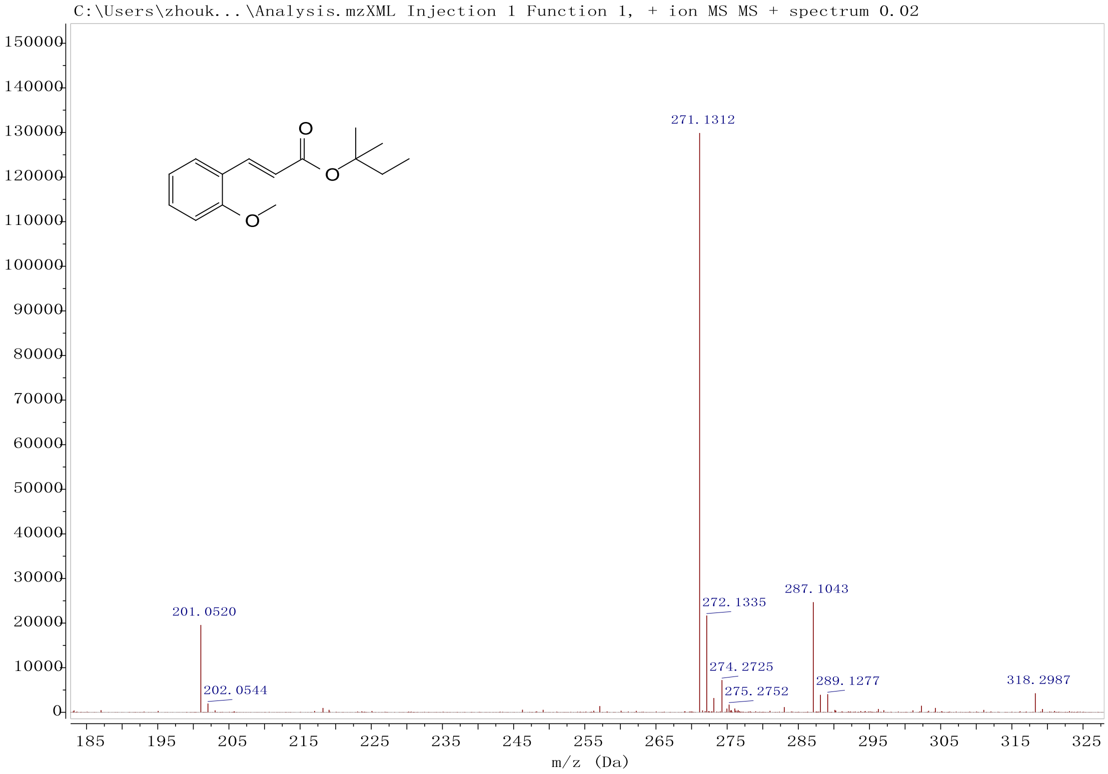

HR-ESI-MS of compound C8

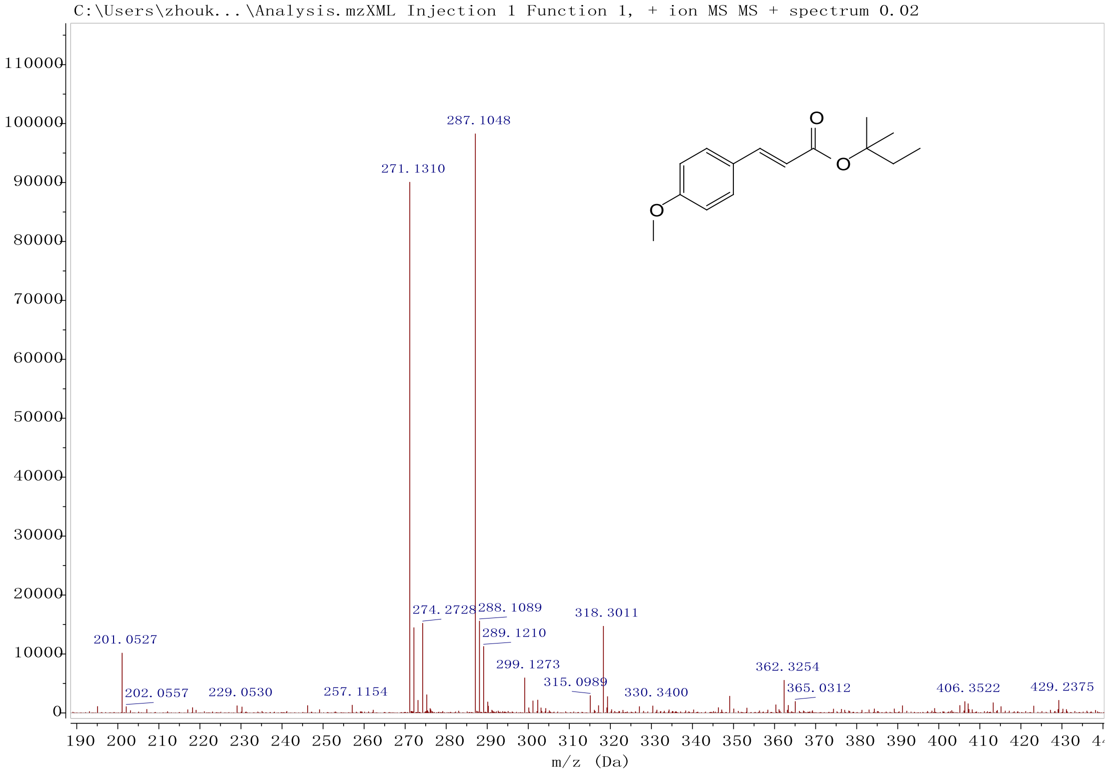

HR-ESI-MS of compound C10

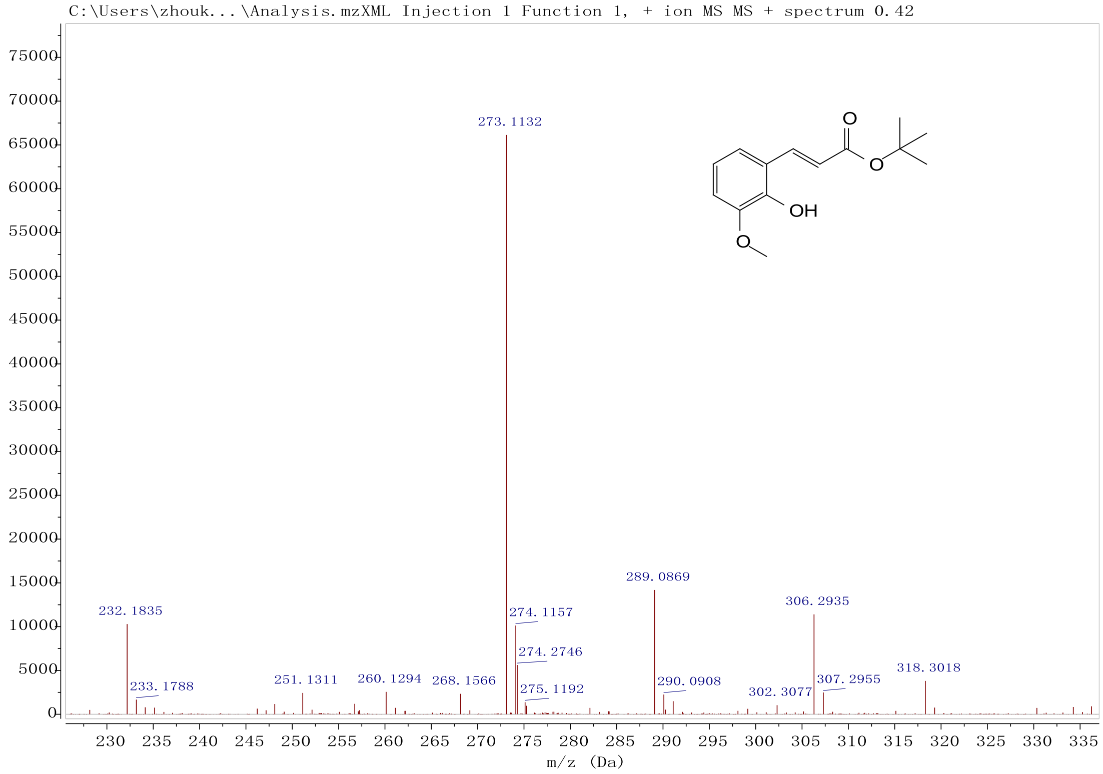

HR-ESI-MS of compound C11

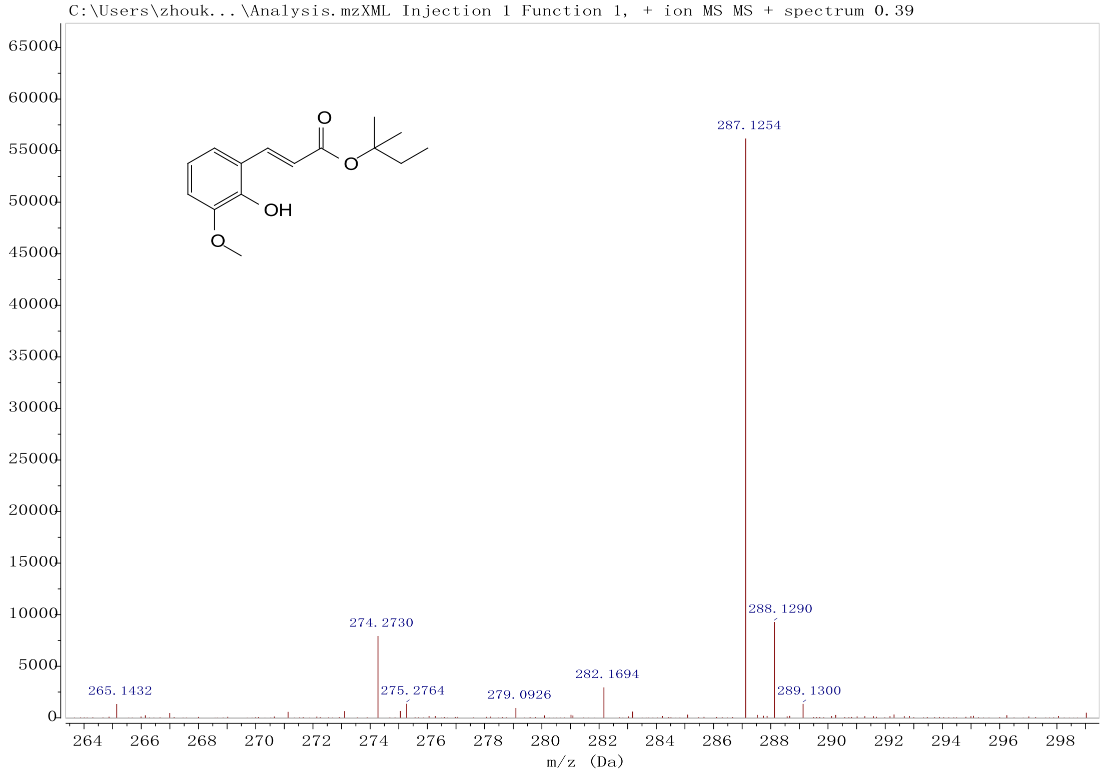

HR-ESI-MS of compound C12

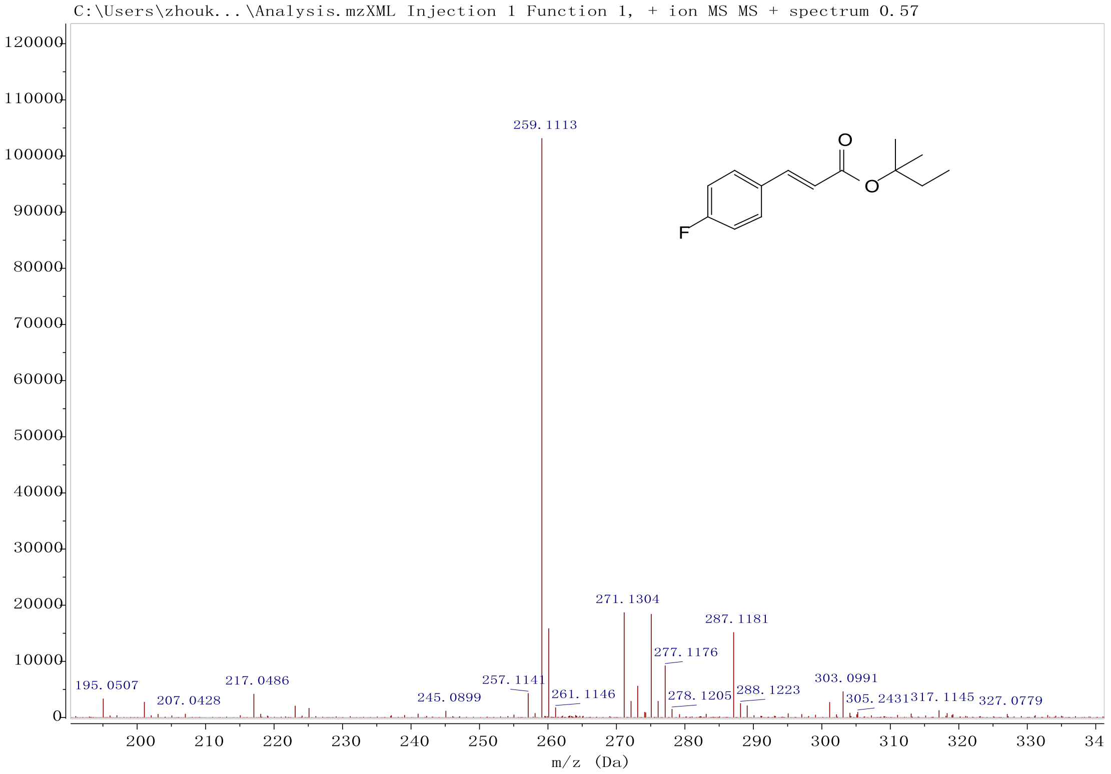

HR-ESI-MS of compound C14

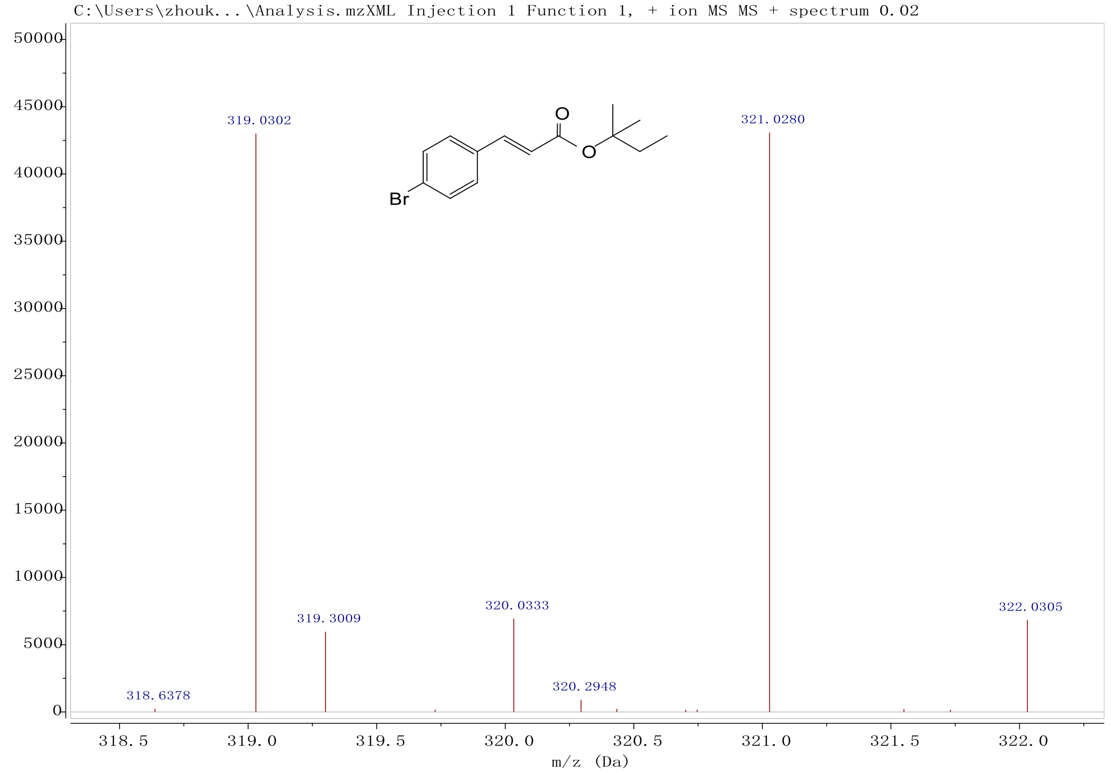

HR-ESI-MS of compound C16

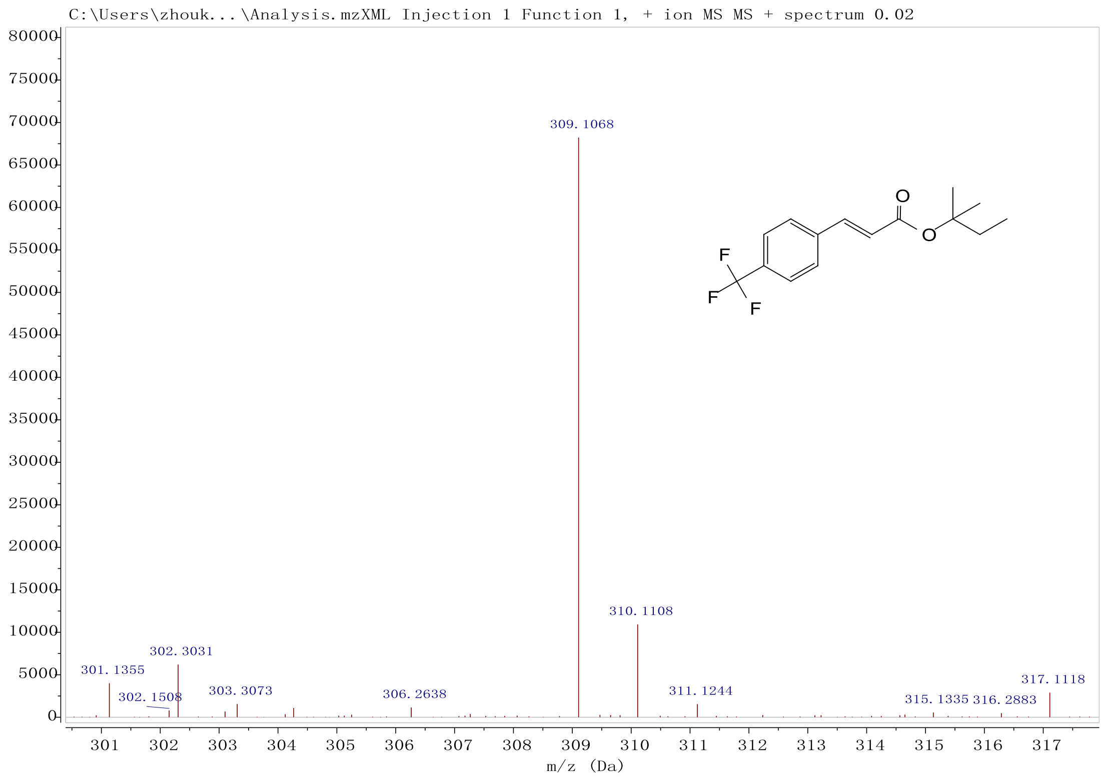

HR-ESI-MS of compound C18

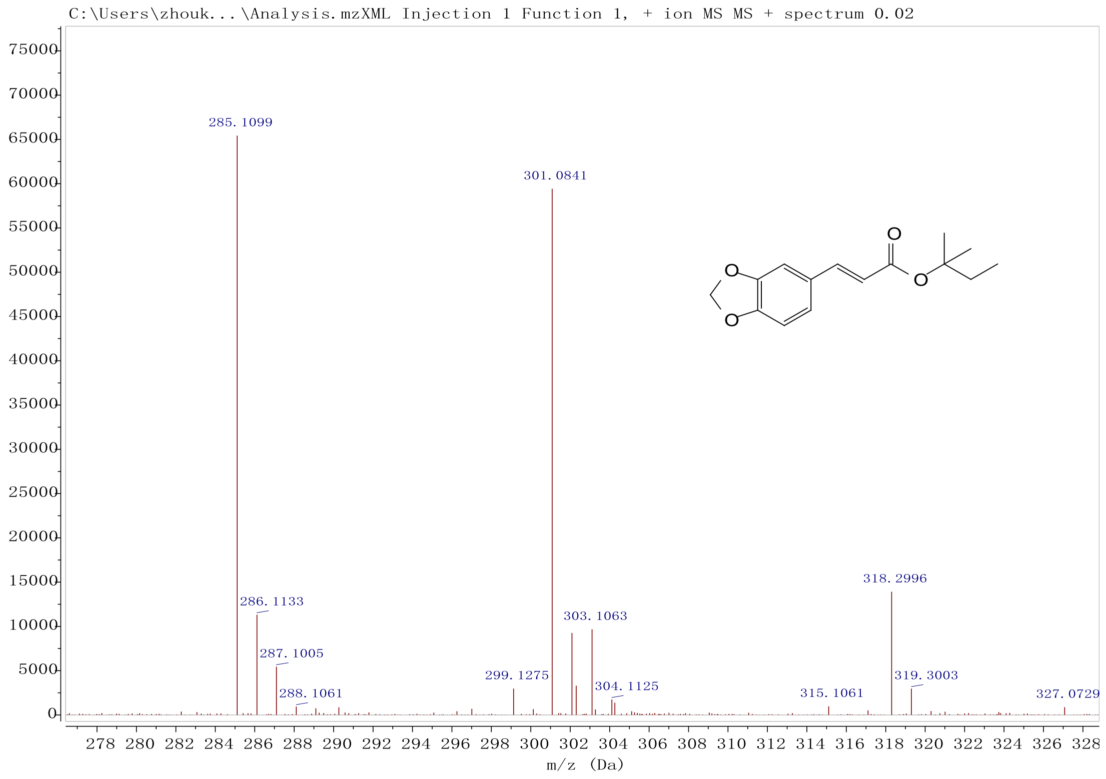

HR-ESI-MS of compound C20

References

1. Chatterjee, A. K.; Choi, T.-L.; Sanders, D. P.; Grubbs, R. H. A general model for selectivity in olefin cross metathesis. *J. Am. Chem. Soc.*, **125,** 11360-11370 (2003).
2. Fan, X.; Zhang, H.-S.; Chen, L.; Long, Y.-Q. Efficient synthesis and identification of novel propane-1,3-diamino bridged CCR5 antagonists with variation on the basic center carrier. *Eur. J. Med. Chem.*, **45,** 2827-2840 (2010).
3. Cao, P.; Li, C.-Y.; Kang, Y.-B.; Xie, Z.; Sun, X.-L.; Tang, Y. Ph_3As-catalyzed Wittig-type olefination of aldehydes with diazoacetate in the presence of $Na_2S_2O_4$. *J. Org. Chem.*, **72,** 6628-6630 (2007).
4. Huang, Z.-Z.; Tang, Y. Unexpected catalyst for Wittig-type and dehalogenation reactions. *J. Org. Chem.* **67,** 5320-5326 (2002).
5. Penafiel, I.; Pastor, I. M.; Yus, M. Heck-matsuda reactions catalyzed by a hydroxyalkyl-functionalized NHC and palladium acetate. *Eur. J. Org. Chem*., **2012,** 3151-3156 (2012).
6. Fontan, N.; Garcia-Dominguez, P.; Alvarez, R.; de Lera, A. R. Novel symmetrical ureas as modulators of protein arginine methyl transferases. *Bioorg. Med. Chem.* **21,** 2056-2067 (2013).
7. Gagey, N.; Neveu, P.; Benbrahim, C.; Goetz, B.; Aujard, I.; Baudin, J.-B.; Jullien, L. Two-photon uncaging with fluorescence reporting: evaluation of the o-hydroxycinnamic platform. *J. Am. Chem. Soc.*, **129,** 9986-9998 (2007).
8. Yang, W.; Chen, H.; Li, J.; Li, C.; Wu, W.; Jiang, H. Palladium-catalyzed aerobic oxidative double allylic C-H oxygenation of alkenes: a novel and straightforward route to α,β-unsaturated esters. *Chem. Comm.* (Cambridge, United Kingdom) **51,** 9575-9578 (2015).
9. Tyagi, V.; Fasan, R. Myoglobin-catalyzed olefination of aldehydes. *Angew. Chem. Int. Ed.* **55,** 2512-2516 (2016).
10. Liu, J.; Shao, C.; Zhang, Y.; Shi, G.; Pan, S. Copper-catalyzed highly efficient ester formation from carboxylic acids/esters and formates. *Org. Biomol. Chem.* **12,** 2637-2640 (2014).
11. Zhan, S.; Tao, X.; Cai, L.; Liu, X.; Liu, T. The carbon material functionalized with NH2+ and SO_3H groups catalyzed esterification with high activity and selectivity. *Green Chem.* **16,** 4649-4653 (2014).
12. Xin, Y.-C.; Shi, S.-H.; Xie, D.-D.; Hui, X.-P.; Xu, P.-F N-heterocyclic carbene-catalyzed oxidative esterification reaction of aldehydes with alkyl halides under aerobic conditions. *Eur. J. Org. Chem.* **2011,** 6527–6531 (2011).
13. Sinha, A. K.; Sharma, A.; Swaroop, A.; Kumar, V. Single step green process for the preparation of substituted cinnamic esters with trans-selectivity. U.S. Pat. Appl. Publ. US 20080045742 A1 20080221, 2008.
14. Sova, M.; Perdih, A.; Kotnik, M.; Kristan, K.; Rizner, T. L.; Solmajer, T.; Gobec, S. Flavonoids and cinnamic acid esters as inhibitors of fungal 17β-hydroxysteroid dehydrogenase: A synthesis, QSAR and modelling study. *Bioorg. Med. Chem.* **14,** 7404-7418 (2006).
15. Frimer, A. A.; Aljadeff, G.; Gilinsky-Sharon, P. Reaction of coumarins with superoxide anion radical (O2-.): facile entry to o-coumarinic acid systems. Israel J. Chem., **27,** 39-44 (1986).
16. Ouimet, M. A.; Stebbins, N. D.; Uhrich, K. E. Biodegradable coumaric acid-based poly(anhydride-ester) synthesis and subsequent controlled release. *Macromolecular Rapid Communications*, **34,** 1231-1236 (2013).
17. Wong, C. T. T.; Lam, H. Y.; Song, T.; Chen, G.; Li, X. Synthesis of constrained head-to-tail cyclic tetrapeptides by an imine-induced ring-closing/contraction strategy. *Angew. Chem. Int. Ed.* **52,** 10212-10215 (2013).
18. Davies, S. G.; Mulvaney, A. W.; Russell, A. J.; A. D. Smith. Synthesis of homochiral β-amino acids. *Tetrahedron: Asymmetry* **18,** 1554–1566 (2007).
19. Zhu, M.-K.; Zhao, J.-F.; Loh, T.-P. Palladium-Catalyzed C-C Bond Formation of Arylhydrazines with Olefins via Carbon-Nitrogen Bond Cleavage. *Org. Lett.*, **13,** 6308-6311 (2011).
20. El-Batta, A.; Jiang, C.; Zhao, W.; Anness, R.; Cooksy, A. L.; Bergdahl, M. Wittig reactions in water media employing stabilized ylides with aldehydes. Synthesis of α,β-unsaturated esters from mixing aldehydes, α-bromoesters, and Ph_3P in aqueous NaHCO3. *J. Org. Chem.* **72,** 5244-5259 (2007).

Table 1. Toxicity Regression Equations and EC_{50} Values of Compounds against four fungi[abc]

Fungi	Compd.	Substituent		Regression equation[a]	R^2	Linear scope (μg/mL)	*P* Value[c]
		R^1,R^2	R^3				
F. solani	**A2**	2-OH	ethyl	y = 1.6115x + 2.7172	0.987	21.4-31.6	<0.0001
	**A4**	4-OH	ethyl	y = 1.1347x + 3.5498	0.967	13.8-25.7	<0.0001
	**A8**	2-OAc	ethyl	y = 1.394x + 2.8863	0.994	28.8-37.2	<0.0001
	**A25**	2-OH-3-OMe	ethyl	y = 1.54x + 3.0729	0.988	14.8-21.4	<0.0001
	**C1**	2-OH	*t*-butyl	y = 1.3036x + 3.1989	0.991	20.9-28.2	<0.0001
	**C2**	2-OH	*t*-amyl	y = 1.5729x + 2.9619	0.991	17.0-22.9	<0.0001
	**C3**	4-OH	*t*-butyl	y = 1.3385x + 2.847	0.990	34.7-47.9	<0.0001
	**C4**	4-OH	*t*-amyl	y = 1.5562x + 2.718	0.989	24.5-35.5	<0.0001
	**C5**	2-OAc	*t*-butyl	y = 1.8122x + 2.055	0.986	33.9-51.3	<0.0001
	**C6**	2-OAc	*t*-amyl	y = 1.5479x + 2.6269	0.975	25.1-45.7	0.0017
	Kresoxim-methyl			y = 0.4424x + 4.4902	0.953	9.8-20.4	0.0002
	Carbendazim			y = 1.4973x + 4.2223	0.969	2.5-4.4	<0.0001
P. grisea	**A2**	2-OH	ethyl	y = 1.1417x + 3.5575	0.975	13.5-24.0	0.0002
	**A4**	4-OH	ethyl	y = 1.5151x + 2.4408	0.974	36.3-63.1	<0.0001
	**A8**	2-OAc	ethyl	y = 1.3729x + 2.7174	0.992	38.9-55.0	<0.0001
	**A25**	2-OH-3-OMe	ethyl	y = 1.3796x + 2.7254	0.984	36.3-55.0	<0.0001
	**C1**	2-OH	*t*-butyl	y = 1.423x + 3.3108	0.987	12.6-18.6	<0.0001
	**C2**	2-OH	*t*-amyl	y = 1.4423x + 3.2271	0.967	12.3-22.9	<0.0001
	**C3**	4-OH	*t*-butyl	y = 1.5562x + 2.8689	0.993	20.0-26.9	<0.0001
	**C4**	4-OH	*t*-amyl	y = 1.2337x + 3.3207	0.998	21.4-24.5	<0.0001
	**C5**	2-OAc	*t*-butyl	y = 1.4807x + 2.9126	0.973	19.1-33.9	<0.0001
	**C6**	2-OAc	*t*-amyl	y = 1.5108x + 2.8926	0.983	20.0-30.9	<0.0001
	Kresoxim-methyl			y = 0.224x + 4.6039	0.960	36.3-128.8	0.0006
	Carbendazim			-	-	-	-
V. mali	**A2**	2-OH	ethyl	y = 1.9536x + 1.817	0.975	31.6-57.5	0.0017
	**A4**	4-OH	ethyl	y = 1.2061x + 2.8488	0.958	42.7-97.7	0.0007
	**A8**	2-OAc	ethyl	y = 1.7987x + 1.8138	0.956	41.7-87.1	0.0001
	**A25**	2-OH-3-OMe	ethyl	y = 1.612x + 2.9617	0.967	13.5-24.5	<0.0001
	**C1**	2-OH	*t*-butyl	y = 1.4566x + 3.1516	0.981	14.8-23.4	<0.0001
	**C2**	2-OH	*t*-amyl	y = 1.5546x + 2.7645	0.989	22.9-33.1	0.0005
	**C3**	4-OH	*t*-butyl	y = 1.3158x + 3.2576	0.972	15.8-27.5	<0.0001
	**C4**	4-OH	*t*-amyl	y = 1.5982x + 2.6163	0.969	22.9-41.7	<0.0001
	**C5**	2-OAc	*t*-butyl	y = 1.6947x + 2.5177	0.976	22.4-37.2	<0.0001
	**C6**	2-OAc	*t*-amyl	y = 1.3158x + 2.9695	0.962	25.1-49.0	<0.0001
	Kresoxim-methyl			y = 0.7208x + 3.563	0.990	77.6-134.9	<0.0001
	Carbendazim			y = 1.4915x + 4.3406	0.948	1.8-4.1	0.0002
B.dothidea	**A2**	2-OH	ethyl	y = 1.6998x + 2.885	0.978	13.2-22.9	0.0014
	**A4**	4-OH	ethyl	y = 1.3806x + 2.7425	0.985	34.7-52.5	<0.0001
	**A8**	2-OAc	ethyl	y = 1.4795x + 3.0447	0.970	15.1-28.2	<0.0001
	**A25**	2-OH-3-OMe	ethyl	y = 1.5908x + 2.8282	0.992	20.4-26.9	<0.0001
	**C1**	2-OH	*t*-butyl	y = 1.5614x + 3.3489	0.987	9.1-13.8	<0.0001
	**C2**	2-OH	*t*-amyl	y = 1.3327x + 3.6727	0.953	5.9-14.5	0.0002
	**C3**	4-OH	*t*-butyl	y = 1.7567x + 2.6015	0.992	19.5-26.9	<0.0001
	**C4**	4-OH	*t*-amyl	y = 2.3243x + 1.5947	0.996	25.7-33.1	0.0001
	**C5**	2-OAc	*t*-butyl	y = 1.4832x + 3.315	0.975	9.5-18.2	<0.0001
	**C6**	2-OAc	*t*-amyl	y = 1.7367x + 2.7259	0.992	17.4-24.0	<0.0001
	Kresoxim-methyl			y = 0.5187x + 4.6707	0.976	3.0-5.8	0.0002
	Carbendazim			y = 1.2061x + 4.3281	0.997	3.3-4.0	<0.0001

[a]y: Probability of average inhibition rate; x: lg[concentration(μg/mL)]. [b]Confidence interval of EC_{50} (μg/mL) at 95% probability. [c]The difference between the data with the different lowercase letters within a column is significant ($P < 0.05$).
